# Phytochemical composition of *Lagenaria siceraria* fruits from KwaZulu-Natal and Limpopo, South Africa

**DOI:** 10.1016/j.fochx.2024.101338

**Published:** 2024-03-30

**Authors:** Lungelo Given Buthelezi, Sydney Mavengahama, Julia Sibiya, Charmaine Nontokozo Mchunu, Nontuthuko Rosemary Ntuli

**Affiliations:** a Department of Agriculture, Faculty of Science, Agriculture and Engineering, University of Zululand, KwaDlangezwa 3886, South Africa; b Food Security and Safety Area, Faculty of Natural and Agricultural Science, North-West University, Mmabatho 2745, South Africa; c School of Agriculture, Earth and Environmental Sciences, University of KwaZulu-Natal, Pietermaritzburg 3201, South Africa; d KZN Department of Agriculture & Rural Development, Soil Fertility and Analytical Services, 01 Cedara Road, Pietermaritzburg 3200, South Africa; e Department of Botany, Faculty of Science, Agriculture and Engineering, University of Zululand, KwaDlangezwa 3886, South Africa

**Keywords:** *Lagenaria siceraria*, Phytochemicals, GC–MS, PCA, OPLS-DA

## Abstract

*Lagenaria siceraria* (Molina) Standley is a food and medicinal source with anti-proliferative, anti-fertility, anti-HIV and anti-cancerous properties. The current study investigated the phytochemical constituents of L. *siceraria* fruits using gas chromatography/mass spectrometry (GC–MS). Five isoprenoids present in all investigated landraces were 1-Dodecene, 2,3-Dimethyldodecane, E-15-Heptadecenal, Eicosane, and Tridecane, 6-propyl. Lighter metabolites such as 1-Dodecene and 2,3-Dimethyldodecane were recorded at a shorter retention time range of 9.08–16.29 min over a lower relative peak area ranging from 1.09 to 6.97%. However, heavier compounds (E-15-Heptadecenal, Eicosane and Tridecane, 6-propyl) had a longer retention time range of 13.42–18.00 mins over a higher relative peak area range of 2.25–11.41%. Cluster analysis grouped landraces into 5 clusters (I -V) according to their fruit and seed attributes, and isoprenoid units significant to each cluster. Terpenoids were the prominent phytochemicals present in fruits. This is the most comprehensive study on the fruit phytochemical constituents of different L. *siceraria* landraces to date.

## Highlights

• This study identified 681 unique phytochemicals from L. *siceraria* landraces.
• Terpenoids were the most prominent compounds found in all landraces.
• Landraces from similar origins, comparable fruit and seed attributes shared similar bioactive molecules.

## 1 Introduction

*Lagenaria siceraria* (Molina) Standley of the Cucurbitaceae family is used for food and in many traditional medicine systems (Saeed et al., 2022). Due to the sessile nature of the plants, they are vulnerable to herbivory (Divekar et al., 2022). To overcome this, plants produce a defensive mechanism in the form of active biomolecules (Divekar et al., 2022). These biomolecules include terpenoids, esters, ethers, organic acids, alkaloids, amino acids, and polyphenols (Chen et al., 2023; de Alvarenga et al., 2021). These secondary metabolites are found in leaves, flowers, fruits and seeds (Zaheer et al., 2021). They are associated with various biological activities such as growth factors, attracting pollinators, defence against mechanical and pathogenic distress (Divekar et al., 2022; Nawade et al., 2019). Isoprenoids are the most diverse and abundant class of these secondary metabolites (Chen et al., 2023). They are further subclustered into hemiterpenes, monoterpenes, sesquiterpenes, diterpenes, sesterpenes, triterpenes, tetraterpenes, and polyterpenes (Abdallah & Quax, 2017; Perveen, 2018). These secondary metabolites are responsible for essential agronomic attributes such as sweet palatability and attractive aromatic fruit scents (de Alvarenga et al., 2021). Furthermore, they are also associated with significant medicinal properties such as anti-bacterial, anti-fungal, anti-diabetic, anti-cancerous, anti-inflammatory, and antioxidant (Chen et al., 2023; Zaheer et al., 2021).

Tender fruits of L. *siceraria* are traditionally used as a cardiotonic, general tonic, liver tonic for liver disorders, an aphrodisiac, pain relief, anti-inflammatory, expectorant, and diuretic agent promoting proper kidney function (Deshpande et al., 2008; Saeed et al., 2022). The above health benefits are an indication of the exceedingly high content of isoprenoids and organic acids (Sosnowska, Kajszczak, & Pods˛edek, 2022). Tender fruits remedy asthmatic and other bronchial disorders and they contain phytochemicals such as terpenoids, saponins, flavonoids, polyphenolics, tannins, cucurbitacin; B, D, G, H and 22-deoxy cucurbitacin, which are responsible for the bitterness in cucurbits (Ahmed, Ejaz, Saeed, & Dar, 2016; Roopan, Rajeswari, Kalpana, & Elango, 2016). The sterols; fucosterol and campesterol are also found in the fruit pulp along with flavone-C glycosidase as well as lagenin, a ribosome-inactivating protein with antiproliferative, antifertility, anti-HIV and anti-cancerous activities (Ahmed et al., 2016; Lleola, Omodaru, & Filua, 2019; Wang, Cao, Hao, & Wu, 2022). In addition, *L. siceraria* seeds are also high in phytochemicals, vitamins, mineral elements, amino acids, and lipids identifying them as a source of protein, micro and macronutrients (El-Rahman, Mahmoud, Sayed, & El-Latif, 2022).

The presence of secondary metabolites, medicinal properties, and uses of L. *siceraria* is well documented in the literature. However, former studies did not quantify phytochemicals in fruits apart from reporting their traces in L. *siceraria* leaves. Hence, the objective of the current study was to explore the phytochemical constituent profile of *Lagenaria siceraria* fruits from northern KwaZulu-Natal and Limpopo, South Africa using the GC–MS technique. Multiple statistical methods were (principal component analysis (PCA), orthogonal partial least squares-discriminant analysis (OPLS-DA), and Agglomerative hierarchical cluster analysis (AHC)) carried out to explore the variation of phytochemical profiles of different landraces.

## 2 Materials and methods

### 2.1 Germplasm sourcing and field layout

**Fig. 1:**
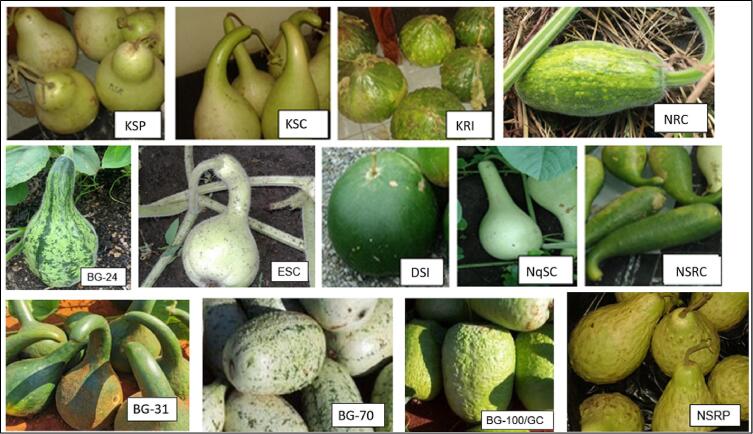
*Lagenaria siceraria* landraces mentioned in Table 1.

Thirteen *Lagenaria siceraria* landraces from different agro-climatic regions in northern KwaZulu-Natal and Limpopo, South Africa were investigated (Table 1). Landraces from KwaZulu-Natal were named according to their area of origin represented by the first letter, fruit texture represented by the second letter and fruit shape represented by the third letter (Table 1). Landraces from Limpopo were named by previous investigators based on their entry number and distinguished by their fruit and seed traits (Mashilo et al., 2017; Mashilo, Shimelis, & Ngwepe, 2021; Mashilo, Shimelis, & Odindo, 2016). Seeds of the landraces were collected from Ga-Phasa (23.4057° S, 29.1557° E), Kgohloane (23.4739° S, 29.2213° E), Khangelani (29.0106° S, 31.2211° E), Ndumo (26.9342° S, 32.2824° E), Emkhandlwini (28.508° E, 31.7002° E), Nquthu (28.2195° S, 30.6746° E), and Dundee (28.1650° S, 30.2343° E). The field experiment was conducted over two summer seasons, September 2020–January 2021 and September 2021–January 2022. The experiment was conducted in the vegetable field unit of the Department of Botany, Faculty of Science, Agriculture and Engineering, University of Zululand, KwaDlangezwa campus (28.51° S, 31.50° E) with a sub-tropical climate (Ntuli & Zobolo, 2008). The KwaDlangezwa area has a daily mean temperature of 28.4 °C in summer and 14.5 °C in winter (Nelson, Khama, & Cedric, 2014). The study area receives an annual rainfall ranging from 299.95 to 350.02 mm (Naidoo et al., 2016). (See Fig. 1.)

**Table 1:** Descriptive characteristics of samples investigated (Buthelezi, Mavengahama, Sibiya, & Ntuli, 2023).

Prov	LR	Area	Fruit colour	Fruit texture	Fruit shape	Seed type	Seed colour	Seed texture	Seed size	Seed line	Seed shape
KZN	KSP	Khangelani	Pale green	Smooth	Pear	Asiatica	Brown	Leathery	Large	Present	Slightly oblong to rectangular
KZN	KSC	Khangelani	Pale green	Smooth	Curvilinear	Asiatica	Brown	Leathery	Large	Present	Slightly oblong to rectangular
KZN	KRI	Khangelani	Green	Rough	Isodiametric	Siceraria	Dark brown	Leathery	Large	Present	Slightly oblong to rectangular
KZN	NRC	Ndumo	Dark green	Rough	Cylindrical	Siceraria	Creamy brown	Smooth	Small	Absent	Oblong
KZN	NSRC	Nquthu	Green	Semi-rough	Curvilinear	Intermediate	Brown	Leathery	Medium	Present	Slightly oblong
KZN	NSRP	Nquthu	Pale green	Semi-rough	Pear	Intermediate	Brown	Leathery	Medium	Present	Slightly oblong
KZN	NqSC	Nquthu	Pale green	Smooth	Semi-Curvilinear	Asiatica	Light brown	Leathery	Medium	Present	Slightly oblong
KZN	DSI	Dundee	Dark green	Smooth	Isodiametric	Siceraria	Dark brown	Smooth	Large	Present	Oblong
KZN	ESC	Emkhandlwini	Pale green	Smooth	Curvilinear	Asiatica	Light brown	Leathery	Medium	Present	Slightly oblong
LP	BG-24	Go-Phasa	Pale green	Corrugate	Cavate	Siceraria	Dark brown	Smooth	Small	Absent	Oblong
LP	BG-31	Kgohloane	Dark green	Smooth	Cavate	Intermediate	Brown	Leathery	Large	Present	Slightly oblong to rectangular
LP	BG-70	Go-Phasa	Pale green	Smooth	Pyriform	Asiatica	Light brown	Leathery	Large	Present	Slightly oblong to rectangular
LP	BG-100/GC	Kgohloane	Pale green	Semi-rough	Cylindrical	Asiatica	Light brown	Leathery	Medium	Present	Slightly oblong

The experiment adopted the randomized block design generated by R 4.2.1 software in RStudio platform (Abishkar & Pragya, 2020). Seeds were directly sown onto a 10 cm deep pit with fertilizer NPK 2:3:4(30) applied at planting. Experimental plots were 3 m × 4 m in size and seeds were spaced at an intra-row spacing of 1 m and an inter-row spacing of 2 m. Each plot had 20 plants with a net plot of 6 m^2^ having 6 plants. Each of the thirteen landraces had three replicate plots, which resulted in 39 plots in total bearing an overall of 780 plants. Weeding and insecticide applications were performed when necessary. The field was irrigated to field capacity for the duration of the experiment using a sprinkler system.

### 2.2 Sample preparation

Ten fruits per plot were harvested seven days after anthesis for each landrace. Samples harvested from each plot constituted a replicate (*n* = 3). The harvested fruits were rinsed with tap water and cut into small pieces using a clean stainless-steel knife, sun-dried for 24 h, and then transferred into an oven (Labcon incubator, Model 5016LC) at 65 °C until a constant dry mass was obtained. Dried fruits were ground into powder through a 0.84 mm sieve, using a laboratory grinder (Hammer mill SMC).

### 2.3 GC–MS identification and quantification of phytochemical constituents

Extraction of biologically active compounds for gas chromatography–mass spectrometry (GC–MS) (PerkinElmer GCMS 2400 System) analysis was conducted using the method by Nwiloh, Uwakwe, and Akaninwor (2016), with modifications. One gram of ground sample was weighed and transferred into a 50 ml test tube. Approximately 15 ml of 70% ethanol and 10 ml of 50% *w*/*v* potassium hydroxide were added to the ground sample in the test tube. The content in the test tube was heated in a water bath at 60 °C for 60 min. The reaction product was filtered by Whatman's No. 1 filter paper using the pressure pump (Merck diaphragm vacuum pump, ME 2) at 1.9 m^3^/h. A 20 ml of 70% ethanol, 3 ml of hexane, and 10 ml of hot and cold water respectively passed through the same filter paper to the resulting filtrate (extract) in the collecting tube. This extract was then mixed and transferred into an Erlenmeyer flask, and the extract-collecting flask was rinsed with 10 ml of 10% *v*/v ethanol aqueous solution three times, into the extract. An anhydrous sodium sulphate was used to dry the solution, by its gradual addition and swirling, and separation of the pellets from the extract. The solvent was evaporated by placing the extract in an oven (Labcon incubator, Model 5016LC) at 50 °C. A 100 μl of n-hexane was used to solubilize the sample of which 20 μl was transferred to a vial on the gas chromatography (GC) machine for phytochemical analysis. However, prior to transference into the GC analyser, samples were filtered through short (∼4 mm) silica plugs before injection to remove any highly polar compounds that would stick on and clog the column, as well as any compounds that would combust (as opposed to volatilise into the gas phase) in the injection port such as sugars.

Phytochemical analysis of the sample was conducted using an auto system buck 530 chromatographer in gas phase equipped with an on-column automatic injector, flame ionization detector, and with a Hp88 capillary column (100 m × 0.25 mm internal diameter and 0.25 μm film thickness) from Restek (Bellefonte, PA, USA). Chromatographic conditions were calibrated to: injector temperature 220 °C, detector temperature of 250 °C, oven temperature to 180 °C, injection volume = 1 ml of the sample, hydrogen was used as a carrier gas (165.474 kPa). Samples produced numerous peaks in the gas chromatogram with specific spectrum used to identify compounds. Chemical compounds (analytes) were identified by relating their retention times with those of the polychlorinated biphenyl (PCB) standards found in the National Institute of Standards and Technology (NIST) library. This analysis was conducted at the Department of Chemistry at the University of KwaZulu-Natal, Scottsville campus.

### 2.4 Multivariate analysis

The experiment adopted the randomized block design generated by R 4.2.1 software in RStudio platform with triplicate samples (*n* = 3) for each landrace. The effects of geographic origin were not considered in this study; hence, this may not affect the true reflection of variance. Therefore, to fully understand variation, data was imported to SIMCA 18 software package (Umetrics, Umea, Sweden) for unsupervised principal component analysis (PCA) and supervised orthogonal partial least squares-discriminant analysis (OPLS-DA) modelling. PCA was conducted to identify the main contributors to variability between investigated landraces with regard to their phytochemical profile. The assessment of the OPLS-DA model involved the utilization of R^2^ and Q^2^ values. R^2^ reflects the model's goodness of fit, while Q^2^ signifies its predictive performance. Agglomerative hierarchical cluster analysis was also conducted to study the variations among landraces based on their phytochemical profile using XLSTATS 2023.

## 3 Results

### 3.1 Variation in the presence of secondary metabolites

Some phytochemical compounds detected in L. *siceraria* fruits were unique to each landrace (Supplementary Data 1), whereas others were shared among landraces (Supplementary Data 2a–l). Landraces BG-31, DSI, KSC, NRC, ESC, NqSC, and BG-100/GC, in their descending order, had many (> 50) unique phytochemical compounds whereas others had fewer (< 50) (Supplementary Data 1). Based on the GC–MS analysis, from the 13 landraces, the greatest number of shared phytochemical compounds were observed between two landraces amounting to 114 compounds, whereas 13 landraces only shared five phytochemical compounds (Supplementary Data 2a–l). Phytochemicals identified on investigated L. *siceraria* tender fruits were in various forms of terpenoids, esters, ethers, organic acids, alkaloids, and organic metalloids.

Some of the unique compounds that occur in studied landraces further showed differences in their chemical groups (Supplementary Data 1). Only landrace BG-31 included the chemical groups aliphatic alcohol, chlorinated diterpene, isocyanate, and phenyl pyrimidine (Supplementary Data 1). The Pyridine carboxylic acid, diethyl ester, and iodinated hemiterpene chemical classes were peculiar to landrace BG-70. Furthermore, the presence of the chemical groups Alkadiene, anthracycline, thienopyridine, and diisocyanate further demonstrated landrace DSI's distinctiveness. The chemical classes cyclic ketone, iodinated monoterpene, opioid analgesic, anticonvulsant, and oxime were unique to landrace ESC, and the isoxazole group was exclusive to KRI. Additionally, the unique chemical groups, acyclic nucleoside, Azocane, sugar alcohol, non-essential amino acid, and macrolide supported the distinctiveness of landrace KSC, where chemical groups macrocyclic polyketide, semicarbazone, and amino ketone showed the same for NRC. Moreover, the presence of the chemical classes Uronic acid, Myristoyl, and isocyanide verifies the uniqueness of NqSC, whereas the Xylose chemical class was found exclusively in landrace NSRC. (Supplementary Data 1).

The fruit extract of L. *siceraria* using GC–MS analysis resulted in the identification of the following phytochemical compounds: 1-Dodecene, 2,3-Dimethyldodecane, E-15-Heptadecenal, Eicosane and Tridecane, 6-propyl- present in fruits of all landraces (Supplementary Data 2a). Phytochemical compounds with a lower carbon number on their chemical structure such as 1-Dodecene and 2,3-Dimethyldodecane with 12 and 14 carbon structures were recorded at a shorter retention rate range of 9.08–16.29 min over a lower relative peak area ranging from 1.09 to 6.97%. However, compounds with longer carbon chains ranging from 16 to 20 carbons (E-15-Heptadecenal, Eicosane and Tridecane, 6-propyl) had a longer retention rate range of 13.42–18.00 mins over a higher relative peak area range of 2.25–11.41% (Supplementary Data 2a).

A sum of three compounds was identified among 12 landraces, where Octadecane and Tetradecane, 4-methyl- were absent in KSP, and Octadecane, 5-methyl- in BG-70 (Supplementary Data 2b). Eicosane, 10-methyl-; Pentadecane, 2-methyl-; and Tridecane, 7-propyl- that were detected in 11 landraces each and were absent in BG-31 and NRC; BG-24 and DSI; and BG-31 and ESC, respectively (Supplementary Data 2c). In four compounds that were found in 10 landraces each, 1-Heptadecane was not identified in DSI, ESC and NSRP; Hexadecane, 4-methyl- in BG-24, DSI, and NSRC; Tetradecane, 5-methyl- in ESC, KRI and NSRC; and Trichloroacetic acid, hexadecyl ester in ESC, NSRC and NSRP (Supplementary Data 2d).

Five compounds were identified in nine landraces each, where 1-Undecene, 7-methyl- was absent in BG-70, DSI, KSC, and NSRC; Decane, 3,7-dimethyl- in BG-24, NRC, NSRC, and NSRP; Dodecane, 2,6,11-trimethyl- in BG-31, DSI, KSP, and NRC; Heneicosane, 11-(1-ethylpropyl)- in BG-24, BG-31, BG-100/GC and NSRP; and Undecane, 2-methyl- in BG-31, BG-100/GC, KSC and NSRC (Supplementary Data 2e). A total number of seven compounds were discovered in eight landraces each. The compounds were 11-Methyldodecanol, Heptadecane, 2,3- dimethyl-, Heptadecyl heptafluorobutyrate, Hexadecane, 5-butyl-, Nonane, 5-(2-methylpropyl)-, Pentadecane, 3-methyl- and Tetradecane, 2-methyl-. All compounds in all 8 landraces each had a similar retention rate and relative peak area, except for landrace NqSC which had the longest retention rate in compounds, 11-Methyldodecanol at 18.78 mins and Heptadecyl heptafluorobutyrate at 25.21 mins. Furthermore, the landrace DSI recorded the highest relative peak area for the compound Tetradecane, 2-methyl- at 7.14% (Supplementary Data 2f).

1-Hexanol, 5-methyl-2-(1-methylethyl)-, 1-Pentadecane, 5,5-Diethyltridecane and Hexadecen-1-ol, trans-9- were detected in seven landraces each. However, they were not detected in BG-24, BG-70, BG-100/GC, DSI, KRI, and KSP; in BG-31, DSI, KSP, NRC, NSRC, and NSRP; in BG-31, BG-100/GC, ESC, KSP, NRC and NqSC; and BG-100/GC, DSI, KRI, KSP, NRC, and NSRP, respectively (Supplementary Data 2 g). A total of seven compounds were discovered in six landraces each. Among the seven compounds that were shared by six landraces (Supplementary Data 2 h), 1-Octadecanesulphonyl chloride found in landraces BG-31, BG-100/GC, ESC, KSC, NqSC, and NSRP had the highest retention rate range of 14.60–17.55 mins. Moreover, Tetradecane, 4-ethyl-, present in landraces ESC, KRI, KSC, KSP, NRC and NqSC, recorded the highest contribution to the relative peak area ranging from 0.96 to 7.98% (Supplementary Data 2 h).

Twenty-four compounds were identified in five landraces (Supplementary Data 2i). Out of the 24 compounds, E-14-Hexadecenal, found in landraces; BG-24, BG-31, ESC, KSC and KSP had the highest relative peak area range of 1.50–10.83% in comparison to other shared compounds shared by five landraces.

A varying combination of four landraces shared 19 compounds (Supplementary Data 2j). Of the 19 compounds, 1-Octadecene found in landraces BG-31, BG-70, DSI and KSP had a larger relative peak area ranging from 0.58 to 4.40%. Further, 47 and 114 compounds were shared among three and two landraces, respectively (Supplementary Data 2 k and 2 l). In a total of 47 compounds, KRI and KSC landraces each shared the majority (18 compounds) with others and landrace NSRP shared the least (5 compounds) (Supplementary Data 2 k). However, landraces KRI and KSC only shared the following seven compounds: 1-Oxaspiro[4.4]nonan-4-one, 2-isopropyl-; 2-Bromotetradecane; Borane, 2,3-dimethyl-2-butyl- (dimer); Cyclohexane, (1-butylhexadecyl)-; Nonane, 2-methyl-; Pentadecane, 5-methyl-; and Undecane, 6-cyclohexyl-. In a sum of 114 compounds, landraces KSC and ESC shared compounds in pairs with 11 other landraces apart from BG-31 and BG-100/GC, respectively. Landrace KSC shared a maximum of 27 compounds with a pairing landrace where it shared the most compounds (6) with BG-100/GC. Landrace KSC and BG-100/GC shared the following compounds: 1-Undecene, 8-methyl; Cyclotetradecane,1,7,11-trimethyl-4-(1-methylethyl)-; Octadecane, 1-bromo-; Oxalic acid, hexyl tetradecyl ester; Tridecane, 2-methyl-; and Tridecanenitrile. Whereas the landrace ESC shared 22 compounds in pairing where it shared the most compounds (4) with BG-31. The four compounds were: 1-Tetradecene, 2-decyl-; 2,2,4,5,5- Pentamethyl-3-imidazoline-1-oxyl; 3-Ethyl-6-triflouroacetoxyoctane; and Decane, 1-iodo-.

### 3.2 Multivariate PCA and OPLS-DA analyses

**Fig. 2:**
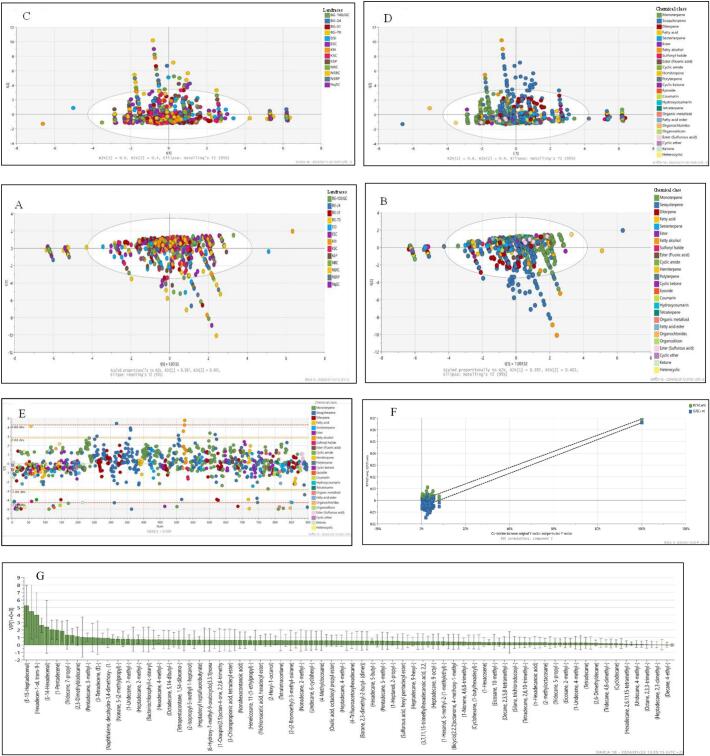
Orthogonality partial least squares discrimination analysis (OPLS-DA) score plot coloured according to landraces (A) and chemical class (B). Principal component analysis (PCA) score plot according to landraces (C) and chemical class (D). Majority of the samples in the OPLS-DA model were within ±2 standard deviation (SD) limit according to Hotelling's T^2^ (95%) (E). 100 cross validation of OPLS-DA model (F) and variable importance plot (VIP) with 95% jack-knifed confidence intervals (G).

An unsupervised clustering method, principal component analysis (PCA) was conducted with the aid of over 600 variables detected in samples (Fig. 2A & B) (Supplementary data 1 & 2). The first two principal components (PC 1 & PC 2) explained 100% of the total variation among the investigated samples. To obtain accurate diversity supervised OPLS-DA score loading plots based on landraces (Fig. 2C) and phytochemicals (Fig. 2D) were also used to show that L. *siceraria* landraces cluster together based on phytochemicals of the same or similar chemical group. The classification was based on the availability of various phytochemicals of different classes and their contribution to variability among thirteen landraces of L. *siceraria* according to the loading plot of OPLS-DA and variable influence in projection (VIP) value. The OPLS-DA score plot (Fig. 2C & D) shows that majority of phytochemicals from all 13 landraces clustered together resembling the results obtained by the PCA (Fig. 2A & B). The model's PCA 1 and 2 cross-validation parameters, R^2^, Q^2^, PCA 1 and 2 eigenvalues, and *p*-value of OPLS-DA were 60 and 40%, 1.00%, 0.98%, 2.99 and 2.01 as well as *p* < 0.01, respectively. What's more, the OPLS-DA model fitted majority of the samples within the acceptable ±2 standard deviation limit (Fig. 2E) and was also validated by 100 iterations of the permutations test (Fig. 2F). To assess the overall impact a metabolite has on variation a projected variable importance plot (VIP) with 95% jack-knifed confidence intervals was also generated (Fig. 2G).

**Fig. 3:**
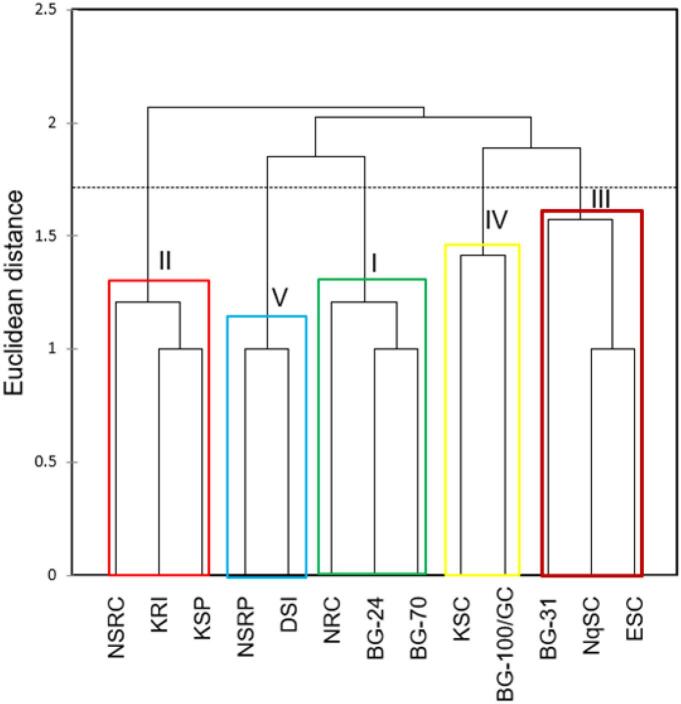
Agglomerative hierarchical cluster showing dissimilarities among L. *siceraria* landraces using unweighted pair group method with arithmetic mean method (UPGMA). Landraces are described in Table 1.

The dendrogram grouped landraces into five clusters (I -V) (Fig. 3). Cluster I consisted of landraces BG-24, BG-70 and NRC and cluster II was comprised of BG-31, ESC and NqSC. Landraces KRI, KSP and NSRC were in cluster III whereas BG-100/GC and KSC were in cluster IV. Landraces DSI and NSRP were grouped in cluster IV.

## 4 Discussion

### 4.1 Phytochemicals unique to various L. *siceraria* landraces

Phytochemicals identified on investigated L. *siceraria* tender fruits were in various forms of terpenoids, esters, ethers, organic acids, alkaloids, amino acids, heterocyclic and polyphenols (Supplementary Data 1 and 2). The landraces of L. *siceraria* contained several phytochemicals that were unique (Supplementary Data 1) and also shared (Supplementary Data 2a-l) among all thirteen landraces. To highlight a few, the landrace BG-24 contained the compound, Molybdenum, (acetato-*O*,*O*′)tris(.eta.3–2-propenyl)- (Supplementary Data 1). Molybdenum-containing compounds are found in protein-rich crops such as legumes, cereals, cereal by-products, and nuts (Sabatino et al., 2019). It is also responsible for optimum vegetative and fruit growth; yield and overall freshness as well as vibrant fruit colours among other berry bearing crops such as *Solanum lycopersicum* L. (Sabatino et al., 2019). The landrace BG-31 contained an isocyanate compound, Dodecane, 1-isocyanato- unique to it (Supplementary Data 1). This compound is largely concentrated in the flowers and growing buds exuding a distinctive repelling unpleasant scent (Grimalt et al., 2021). Moreover, isocyanates are derived from glucosinolates, which are primary constituents of an antiherbivore defensive mechanism plants employ which is stimulated through mechanical degradation (Grimalt et al., 2021). This agrees with a previous study on BG-31 where it produced vigorously growing plants with a significant availability of fruits (Buthelezi et al., 2023).

Landrace BG-70 contained unique compounds such as the aldehyde, Nonanal (Supplementary Data 1). This compound is responsible for the oily fresh green and sweet aroma exuded by fruits and vegetables such as *Prunus domestica* L., *Malus domestica* L. Borkh and *Prunus dulcis* Mill. D. A. Webb (Grimalt et al., 2021). Similarly, landrace DSI boasted unique phenolic compounds; 1,3-Benzenediol, 4,6-dichloro- and 3-cis-Methoxy-5-*cis*-methyl-1R-cyclohexanol which are also responsible for aromatic scents in fruits and tend to vary across different varieties of the same species originating from different origins (Taati, Pilehvar, & Mirazadi, 2022). Furthermore, they are identified as scavengers and inhibitors due to their impressive anti-tumour, anti-bacterial, anti-inflammatory, and antiallergic properties (Taati et al., 2022). The landrace ESC contained the compound Dimenoxadol, which is an opioid analgesic (Supplementary Data 1). These compounds are well-known for treating symptoms of inflammation, swelling, fever, and pain (Sovia & Anggraeny, 2019). However, in some plants such as *Arenga pinnata* Wurmb Merr. (sugar palm fruit) they are responsible for the sweet palatability sap known as sauger which is consumed as a drink or utilized as a raw material for sugar production (Sovia & Anggraeny, 2019). As a result, the landrace ESC can be the sweetest tasting landrace due to the presence of Dimenoxadol.

Further, the landrace ESC contained an anticonvulsant compound, Pregabalin (Supplementary Data 1). In developing countries, rural communities rely heavily on folk medicine to treat various illnesses and disorders such as epilepsy (Drafor, Duah, Ankamah, Kpene, & Mante, 2021). The compound Pregabalin also found in fruits of *Jatropha gossypifolia* L. that are utilized as newer antiepileptic drugs (Drafor et al., 2021). Rural communities are always excluded due to the cost and effects of these drugs (Asghar et al., 2022). However, the landrace ESC has the potential to mitigate this issue due to the financial advantage and agronomic practice required to propagate L. *siceraria* (Sithole & Modi, 2015). Moreover, the use of these drugs has severe consequences such as weight-gain, somnolence, dizziness, and peripheral oedema (Asghar et al., 2022). However, obtaining these compounds from plants and fruits is a more natural way of maintaining good health with fewer side-effects (Asghar et al., 2022). In addition, one of the primary causes of losses in agriculture is the deterioration of fruits post-harvest which is identified by the softening of fruits (Zheng et al., 2022).

Landrace NqSC contains the unique Uronic acid compound 2-Acetamido-2-deoxymannosonic acid, hence, suggestive of a prolonged shelf-life of this landrace (Supplementary Data 1). The presence of Uronic acid in fruits reverses the destruction of fruit wall's structural polysaccharides thus maintaining the intercellular cell wall adhesion of berry crops similar to L. *siceraria* such as *Lycopersicon esculentum* L. preserving the berry for a notably longer period than in the absence of a Uronic acid (Zheng et al., 2022).

### 4.2 Phytochemicals shared among L. *siceraria* landraces

The five compounds, namely, 1-Dodecane, 2,3-Dimethyldodecane, E-15-Heptadecanal, Eicosane, and Tridecane, 6-propyl, which were present in all landraces (Supplementary Data 2a) belonged to the terpenoid class. Terpenoids are the most diverse class of plant secondary metabolites responsible for attracting pollinators, defence against pathogens and herbivores, and protection against environmental stress (Nawade et al., 2019). Terpenes further act as the plants signal centre to various abiotic stress such as heat where they are morphed into thermoprotectants by stabilizing the cellular membranes and stopping proton efflux during high temperatures (Qaderi, Martel, & Strugnell, 2023). The climatic conditions in KwaZulu-Natal (KZN) and Limpopo (LP) are subtropical, featuring extremely hot summers (Ferreira et al., 2023; Ndlovu et al., 2021). This study, conducted during the summer months in KZN, supports the dominant presence of these terpenes in all investigated L. *siceraria* landraces. Moreover, this further supports the absence of flavonoids since their biosynthesis is triggered under cold climatic conditions (Qaderi et al., 2023). Additionally, terpenes tend to be stored and concentrated in trichomes due to their vapor-dispersion nature under high temperatures (Qaderi et al., 2023). Given that cucurbits, including L. *siceraria*, are known to display numerous trichomes over juvenile fruits (Xue et al., 2019), elucidating the prevalence of terpenes in the investigated landraces.

Terpenoids are further subclassified into; hemiterpenes (5 carbon units), monoterpenes (10 carbon units), sesquiterpene (15 carbon units), diterpenes (20 carbon units), sesterpene (25 carbon units), triterpenes (30 carbon units), tetraterpenes (40 carbon units) and polyterpenes (>40 carbon units) (Abdallah & Quax, 2017; Perveen, 2018). These subclasses are regulated by the number of carbon units present as the backbone of a molecule (Perveen, 2018). The monoterpenes, 1-Dodecene and 2,3-Dimethyldodecane and sesquiterpenes, E-15-Hptadecanal and Tridecane, 6-propyl are good indicators of the presence of fatty acid derived compounds such as alcohol, ketones and esters (Hodgkison et al., 2013). These compounds are solely responsible for fruit scent, palatability and preservation due to their ability to readily vaporize at room temperature (de Alvarenga et al., 2021). These terpenoids are linked with diabetic, antibacterial, antifungal, anti-cancerous, anti-inflammatory, and antioxidant properties (Zaheer et al., 2021).

Various bioactive molecules were isolated in selective landraces such as 2-Piperidinone, N-[4-bromo-n-butyl]- (PNbb) which was only found in landraces KRI, ESC, KSC and NqSC (Supplementary Data 2). The bioactive molecule PNbb is a delta-lactam also present in *Punica granatum* L. peels and possesses antimicrobial activities (Al-Bahadily, Shari, Najm, & Al-Salman, 2019). It is also an effective bactericidal inhibitor, making it a good candidate to treat bladder spasms, shrinkage, peptic ulcer inflammation and pancreatitis (Al-Bahadily et al., 2019). However, due to its high toxicity, it is an ideal alternative to synthetic pesticides (Al-Bahadily et al., 2019). Landraces KRI, ESC, and NqSC also contained the phytochemical bacteriochlorophyll-c-stearyl (Supplementary Data 2), which is a lipoxygenase inhibitor (Madhavan, Priyadharshini, & Sripriya, 2021). Lipoxygenases are present in the human body which aids in the stimulation of inflammatory reactions associated with diseases such as cancer, stroke, cardiovascular and neurodegenerative diseases (Lonˇcari'c et al., 2021). However, these can also be supplemented through the consumption of cereal crops (*Triticum aestivum* L., *Zea mays* L., *Avena sativa* L., *Secale cereale* L., and *Hordeum vulgare* L.), legumes (*Vigna radiata* (L.) R. Wilczek., *Phaseolus vulgaris* L., *Pisum sativum* L., *Phaseolus coccineus* L., and *Glycine* max (L.) Merr.) and tubers (*Solanum tuberosum* L.) (Lonˇcari'c et al., 2021). Lipoxygenases are also linked with the synthesis of lipid mediators, prostaglandins and leukotrienes which are activated by mechanical trauma, growth factor and other stimuli acting upon the plant (Funk, 2001). Therefore, the presence of bacteriochlorophyll-c-stearyl is mandatory in the inhibition of these lipid mediators as a crucial disease-preventative mechanism (Funk, 2001).

Phytane is another bioactive molecule that is a plant chlorophyll derivative and another lipid mediator found in landraces ESC, KSP, and NqSC (Supplementary Data 2). It is responsible for a wide range of biological activities, such as, anxiolytic, metabolism-modulating, cytotoxic, antioxidant, autophagy and apoptosis-inducing, antinociceptive, anti-inflammatory, immune-modulating, and anti-microbial effects (Chen et al., 2023; Tapfuma et al., 2020). Other related Cucurbitaceae species such as *Citrullus lanatus* (Thunb.) Matsum. & Nakai and *Cucurbita maxima* Duchesne. also contain phytane in their fruit extracts (Tapfuma et al., 2020). Geranyl isovalerate (GIV) found in landraces BG-70 and NSRP (Supplementary Data 2) is among the least investigated ethnopharmacological compounds commercially utilized as a food flavouring agent and a constituent of essential oils (Iftikhar, Ahmed, & Qamar, 2019; Kumar, Sharma, Thukral, & Bhardwaj, 2015). GIV also possesses antitumor, antihyperglycemic, immunomodulatory, anti-inflammatory, and analgesic properties, thus making it an ideal food supplement and an alternative chemotherapeutic (Rasool, Sharma, Anand, Magani, & Tantravahi, 2021).

Amino acids are building blocks of proteins and precursors of bioactive molecules which are further subclustered into essential, non-essential, and conditionally essential amino acids (Kumar et al., 2015). N-Guanyl proline acid found in landraces BG-70 and NqSC (Supplementary Data 2), is a conditionally essential amino (Hasan & Rima, 2021). The presence of this amino acid indicates the abundance of the protein, proximate content, and medicinal properties in landraces BG-70 and NqSC (Hasan & Rima, 2021; Kumar et al., 2015). Conditionally essential amino acids are non-essential amino acids that become vital as a response to illnesses such as mental and neurotransmission illness, cardiovascular and gastrointestinal health, liver diseases, fatigue, cancer prevention, sepsis, and diabetes (Hasan & Rima, 2021; Odia & Esezobor, 2017).

### 4.3 Multivariate PCA and OPLS-DA analyses

The internal cross-validated PCA and OPLS-DA model resulted in the first two principal components with a good quality of the fit of R^2^ = 1.00 (Fig. 2 A–D) along with the acceptable predictive abilities of Q^2^ = 0.978 due to the close clustering of secondary metabolites, indicating the similarities in phytochemical profiles present in all landraces (An et al., 2019; Plazas, Casoti, Murillo, Da Costa, & Cuca, 2019). The closer the parameters are to 1.0 with a significant value of *p* < 0.01 the better the stability and predictability of the models (An et al., 2019). The possible reason for similar metabolites profiles and overlapping clustering could be the planting environment such as the latitude, temperature, and soil profile which was the same for all landraces though they originate from various origins (Long et al., 2023). About 95% of the samples of the OPLS-DA model are within the limit agreement of two standard deviations suggesting a high probability (Carkeet, 2015). There were some outliers observed possibly due to the degree variance (Carkeet, 2015) in chemical classes which included organic metalloid, organosilicon, epoxide, coumarins, terpenoids, ester and heterocyclic compounds (Fig. 2E). The intercepts of R^2^ (0.0, 0.0003) and Q^2^ (0.0, −0.006) with the vertical coordinates were less than one, the intercepts of Q^2^ in vertical coordinates was less than zero indicating that the established OPLS-DA model had acceptable robustness (Fig. 2F) (Fu et al., 2021). The projected variable importance plot (VIP) further identified phytochemicals with a VIP value >1 as significant contributors to the importance index (Fu et al., 2021). This included the phytochemicals; E-15 Heptadecanal (Sesquiterpene), Hexadecen-1-ol, trans-9- (Fatty alcohol), E-14 Hexadecenal (Fatty alcohol), 1-Pentadecene (Sesquiterpene), Tridecane, 7-propyl- (Sesquiterpene), 2,3-Dimethyldodecane (Monoterpene), and Pentadecane, 3-methyl- (Sesquiterpene) (Fig. 2G) (Supplementary Data 1 & 2). This further supports the dominant presence of terpenoids (monoterpenes, diterpenes, sesquiterpenes, and sesterterpenes) (Fig. 2A–D) which are triggered under hot climatic conditions (Qaderi et al., 2023).

### 4.4 Agglomerative hierarchical cluster analysis

The clustering of landraces into five groups (Fig. 3) could be facilitated by the area of origin, fruit, and seed characteristics as well as the similar bioactive molecules they share (Supplementary Data 1 and 2a-l). Landraces BG-24 and BG-70 both from Go-Phasa with pale, green-coloured fruits grouped with NRC from Ndumo which shared similar fruit (rough texture) and seed characteristics (seed type, colour, texture, and size) with BG-24 in the first cluster. All three landraces (Cluster I) were associated with a total of eighteen phytochemicals of which 33.3% were monoterpenes, 11.1% were diterpenes, 50% were sesquiterpenes and fatty acids made up the remaining 5.6%. The abundance of sesquiterpenes over other isoprenoids suggests that these landraces possess antibacterial properties with a profound bitter taste (Chadwick, Trewin, Gawthrop, & Wagstaff, 2013). Furthermore, from a previous study conducted on these landraces, cluster I grouped landraces with relatively less vigorously growing vegetative modules while producing notably smaller fruits (Buthelezi et al., 2023).

Cluster II was associated with landraces NSRC, KRI and KSP from northern KwaZulu-Natal with an even distribution of isoprenoid units. Of the nineteen phytochemicals they shared, 26.3% were monoterpenes, 21% were diterpenes, 36% were sesquiterpenes, and 5.3% were sesterterpenes, fatty alcohol and ester, each. The different types of isoprenoid units were distributed evenly though sesquiterpenes were dominant among these landraces (Supplementary Data 1 and 2a-l). The presence of monoterpenes and diterpenes at similar proportions suggests that these landraces are more palatable and fragrant since monoterpenes and diterpenes are responsible for the aromatic sweet flavouring and scent in other fruiting crops such as *Rubus ideas* L. commonly known as raspberries (Hampel, Swatski, Mosandl, & Wüst, 2007). Furthermore, from a previous study, landraces NSRC, KRI and KSP produced the longest and widest fruits among all other landraces (Buthelezi et al., 2023). Therefore, their impressively large fruits can be associated with the presence of the phytochemical oxalic acid, propyl-tridecyl ester (Supplementary Data 2i). Oxalic acid promotes fruit growth while increasing shelf-life which is well observed in *Citrus limon* (Linn.) Burm.f. (Serna-Escolano et al., 2021). Such desirable agronomic attributes advocate the selection of landraces NSRC, KRI and KSP for possible purification and mass production.

Landraces BG-31, ESC and NqSC formed cluster III which had an equal proportion of monoterpenes and diterpenes at 45% combined and sesquiterpenes at 45%, as well as fatty alcohols and sulfonyl halides, contributing 5% each (Supplementary Data 2a-l). These landraces contain 1-octadecanesulphonyl chloride, which is an active sulfonyl halide molecule that can be utilized as a pesticide (Suzuki, Ootaka, Onoue, & Onoue, 2021). The presence of this phytochemical can assist plants against pests such as spider mites which are responsible for leaf discoloration, defoliation, bud and fruit shedding, reduced fruit yield and quality (Suzuki et al., 2021). From previous investigations cluster IV grouped landraces BG-100/GC and KSC based on similar fruit (colour, shape, and mass) and seed (type, texture, line and shape) attributes (Buthelezi et al., 2023). Cluster V had a combination of landraces with the longest fruits (NSRP) and with wide and heaviest fruits (DSI) (Buthelezi et al., 2023). Landraces DSI and NSRP both possess the aliphatic hydrocarbon hexane,2,4,4-trimethyl which was unique to these two landraces (Supplementary Data 2 l). Isoprenoids are a structurally diverse class of bioactive molecules with the slightest isomerism specific to function such as fruit growth and development as well as the palatability of fruits (Chen et al., 2023; Maamoun, El-akkad, & Farag, 2021). The activation of these bioactive molecules is triggered through mechanical rupture such as chewing of the fruit which is realized in a sweet and aromatic palatable taste sensation (Maamoun et al., 2021). In addition, this corresponds with a previous investigation on landraces DSI and NSRP which were clustered with landraces displaying the longest, widest, and heaviest fruits (Buthelezi et al., 2023).

## 5 Conclusion

*Lagenaria siceraria* is a significant food and medicinal plant with various applications like anti-bacterial, anti-fungal, diabetic, anti-cancerous, anti-inflammatory, and antioxidant. GC–MS was executed to examine the phytochemical composition profile of L. *siceraria* landraces from northern KwaZulu-Natal and Limpopo, South Africa. The L. *siceraria* fruits contained terpenoids, delta-lactam, chlorophyll derivatives, alkaloids, organic acids, and amino acids. These molecules are associated with essential biological activities and medicinal properties. Very few compounds were present in all landraces. Bioactive molecules with larger carbon-to‑carbon structures have a longer retention over a higher relative peak area. Landraces were grouped according to area of origin, fruits and seed traits as well as the presence of unique biomolecules. Landraces DSI, KRI, KSP, NSRC and NSRP can be recommended for possible purification and mass production based on their desirable fruit sizes and possible shelf-life attributes. Large-scale cultivation of these landraces would result in the extraction of compounds such as Aclarubicin, Isopropyl myristate, o-Acetyl-l-serine, and oxalic acid from DSI; Muscimol, Octanal, and Vinyl lauryl ether from KRI; and many other compounds given in the supporting material. In addition, 1-Pentacontanol and Streptovitacin A can be isolated from KSP; alpha-D-Xylofuranose, Cyclic 1,2:3,5-bis (ethylboronate), Arginine and Bisnorallocholanic acid gathered from NSRC; 11-Tricosene, 3-Decenoic acid and Cyclodecanone, oxime from NSRP. The geographic origin was not considered in the current study as the landraces of different origins were grown in the same field. Sampling at their geographic origin could be implemented to correctly profile the metabolites that may be suppressed or over expressed in foreign growing conditions.

## Institutional review board statement

Not applicable.

## Funding

This research was funded by the National Research Foundation (NRF) Thuthuka Funding Instrument, Reference–TTK210428597773, Grant number—138362.

## CRediT authorship contribution statement

**Lungelo Given Buthelezi:** Writing – review & editing, Writing – original draft, Methodology, Investigation, Formal analysis, Data curation, Conceptualization. **Sydney Mavengahama:** Writing – review & editing, Validation, Supervision, Resources, Project administration, Formal analysis, Conceptualization. **Julia Sibiya:** Writing – review & editing, Conceptualization, Formal analysis, Project administration, Supervision. **Charmaine Nontokozo Mchunu:** Writing – review & editing, Software, Resources. **Nontuthuko Rosemary Ntuli:** Resources, Project administration, Funding acquisition, Formal analysis, Conceptualization, Supervision, Writing – original draft, Writing – review & editing.

## Declaration of competing interest

All authors declare no conflicts of interest pertaining to this manuscript.

Everyone contributed in a positive manner and agreed to publish the work.

## Data Availability

The research data can be requested from the authors.

## Appendix A Supplementary data

Supplementary Data 1: Phytochemical compounds unique to each of the thirteen *L. siceraria* landraces identified using accurate mass LC-MS^n^ in positive ESI mode. Image 1
