# Supplementary material for: Phytochemical composition of Lagenaria siceraria fruits from KwaZulu-Natal and Limpopo, South Africa
Source: Food Chem X. 2024 Mar 30;22:101338. doi: 10.1016/j.fochx.2024.101338 (PMC11016956; doi:10.1016/j.fochx.2024.101338)
Supplement: Supplementary file 1 — Supplementary Data 1: Phytochemical compounds unique to each of the thirteen L. siceraria landraces identified using accurate mass LC-MSn in positive ESI mode. [file mmc1.docx]

Supplementary Data 1: Phytochemical compounds unique to each of the thirteen *L. siceraria* landraces identified using accurate mass LC-MS^n^ in positive ESI mode.

| LR | Phytochemical Compound | Rt (mins) | Accurate Mass | Molecular Ion [M+H]^+^ | Chemical formula | Fragment Ions [M+H]^+^ |
| --- | --- | --- | --- | --- | --- | --- |
| BG-24 | 1,1'-Bicyclohexyl, 2-methyl-, trans- | 14.68 | 180.1879 | 180 | C13H24 | 97.10/55/41/67/81 |
|  | 1,3,5-Triazine, 2-amino-4-methyl-6-dimethylamino- | 15.38 | 153.1016 | 153 | C6H11N5 | 153/42/69/138/124 |
|  | 1,7-Octadiene, 2,3,3-trimethyl- | 15.54 | 152.1566 | 152 | C11H20 | 83/55/41/84/69 |
|  | 1-Hydroxy-4-(1-hydrazonoethyl)-2,2,5,5-tetramethyl-3-imidazoline | 15.13 | 198.1482 | 198 | C9H18N4O | 57/42/168/152/41 |
|  | 1-Hydroxymethyl-7,7-dimethyl-bicyclo[2.2.1]heptane-2,3-dione | 15.37 | 182.0943 | 182 | C10H14O3 | 85/55/41/81/69 |
|  | 1-Nonen-3-one, 2-methyl- | 13.70 | 154.1358 | 154 | C10H18O | 41/69/84/43/27 |
|  | 2-(2-Methyl-propenyl)-cyclohexylamine | 14.95 | 153.1518 | 153 | C10H19N | 56/82/97/43/110 |
|  | 2,5-Heptadecadione | 23.70 | 268.2403 | 268 | C17H32O2 | 128/57/85/113/71 |
|  | 2-Hexyldodecyl butyrate | 15.76 | 340.3343 | 340 | C22H44O2 | 71/43/57/111/97 |
|  | 2-Methylenecyclohexanol | 15.75 | 112.0888 | 112 | C7H12O | 41/55/83/97/69 |
|  | 4-Heptafluorobutyroxytetradecane | 14.15 | 410.2056 | 410 | C18H29F7O2 | 41/55/69/83/97 |
|  | Butanal, 3-methyl-2-methylene-, diethylhydrazone | 25.25 | 168.1628 | 168 | C10H20N2 | 97/139/168/41/58 |
|  | cisZ-11,12-Epoxytetradecan-1-ol | 23.69 | 228.209 | 228 | C14H28O2 | 41/55/67/82/95 |
|  | Cyclohexane, undecyl- | 15.32 | 238.2662 | 238 | C17H34 | 83/55/41/67/84 |
|  | Dodecane, 1,1'-oxybis- | 10.53 | 354.3864 | 354 | C24H50O | 57/71/43/169/85 |
|  | Dodecane, 6-cyclohexyl- | 15.33 | 252.2818 | 252 | C18H36 | 55/83/41/97/111 |
|  | Heptafluorobutyric acid, hexadecyl ester | 15.76 | 438.2369 | 438 | C20H33F7O2 | 57/43/83/97/41 |
|  | Molybdenum, (acetato-O,O')tris(.eta.3-2-propenyl)- | 21.05 | 278.213 | 280 | C11H18MoO2 | 43/195/39/235/152 |
|  | N-[4-Cyclododecylaminobutyl]aziridine | 14.94 | 280.288 | 280 | C18H36N2 | 70/56/98/41/55 |
|  | Nonadecane, 3-methyl- | 15.06 | 282.3288 | 282 | C20H42 | 57/43/41/71/85 |
|  | Pentadec-7-ene, 7-bromomethyl- | 25.26 | 302.161 | 302 | C16H31Br | 41/55/69/83/97 |
|  | Propanedinitrile, (1,2,2-trimethylpropyl)- | 14.53 | 150.1158 | 150 | C9H14N2 | 57/41/135/95/43 |
|  | Tetracosane, 11-decyl- | 23.71 | 478.5481 | 478 | C34H70 | 57/43/71/85/41 |
|  | Trichloroacetic acid, tridecyl ester | 10.44 | 344.1079 | 344 | C15H27Cl3O2 | 57/41/83/97/111 |
| BG-31 | (3-tert-Butyl-5-hydroxymethyl-cyclohex-2-enyl)-methanol | 16.30 | 198.162 | 198 | C12H22O2 | 57/41/93/43/79 |
|  | 1-(1-Methoxycyclopropyl)-3-methylbut-2-en-1-ol | 16.16 | 156.115 | 156 | C9H16O2 | 41/85/67/55/27 |
|  | 1,1'-Bicyclohexyl, 4-propoxy-4'-propyl- | 15.37 | 266.2611 | 266 | C18H34O | 57/41/81/55/206 |
|  | 1,2,3,6,9-Pentaazaspiro[4.4]non-2-ene, 1,4,4,6,9-pentamethyl- | 10.72 | 197.1642 | 197 | C9H19N5 | 127/98/42/70/56 |
|  | 1,2-Cyclohexanediamine, N,N,N',N'-tetramethyl | 11.57 | 170.1784 | 170 | C10H22N2 | 71/84/42/58/170 |
|  | 10-Octadecenal | 15.91 | 266.2611 | 266 | C18H34O | 57/43/82/41/96 |
|  | 1-Chloroeicosane | 13.15 | 316.2899 | 316 | C20H41Cl | 57/55/41/71/127 |
|  | 1-Dodecanol, heptafluorobutyrate | 13.01 | 382.1743 | 382 | C16H25F7O2 | 55/69/41/83/97 |
|  | 1-Glyceryl ricinoleate | 16.38 | 372.2876 | 372 | C21H40O5 | 55/61/166/99/97 |
|  | 1-Heptanal, 3,5,5-triethyl- | 14.41 | 198.1984 | 198 | C13H26O | 57/41/55/95/151 |
|  | 1-Methyl-4-piperidinyl 3-ethylphenylcarbamate | 15.87 | 262.1682 | 262 | C15H22N2O2 | 97/98/82/55/262 |
|  | 1-Oxa-3-azaspiro[4.5]decane-3-acetamide, N-(4-fluorophenyl)-4-hydroxy-4-methyl-2-oxo- | 12.65 | 336.1486 | 336 | C17H21FN2O4 | 43/111/41/137/95 |
|  | 1S,3R,4S,5R,6S-1-Hydroxy-2,2,3,4,5,6-hexamethyl-8-oxo-7,9-dioxatricyclo[4.2.1.0(3,5)]nonane | 25.25 | 240.1362 | 240 | C13H20O4 | 43/153/125/70/41 |
|  | 2(3H)-Benzofuranone, hexahydro-4,4,7a-trimethyl- | 15.96 | 182.1307 | 182 | C11H18O2 | 43/69/139/41/167 |
|  | 2,3-Dibromobutyric acid | 14.51 | 243.8733 | 244 | C4H6Br2O2 | 41/85/165/45/27 |
|  | 2,4-Decadien-1-ol, (E,E)- | 15.88 | 154.1358 | 154 | C10H18O | 55/83/41/67/98 |
|  | 2,6S-Diethyl-3,5S-dimethyl-3,4-dihydro-2H-pyran | 14.30 | 168.1515 | 168 | C11H20O | 99/168/86/69/55 |
|  | 2-Acetyl- 1,5-dimethyl-8-oxabicyclo [3.2.1] octane | 13.16 | 182.1307 | 182 | C11H18O2 | 43/81/182/111/124 |
|  | 2-Butyloxycarbonyloxy-1,1,10-trimethyl-6,9-epidioxydecalin | 19.05 | 326.2094 | 326 | C18H30O5 | 41/81/57/43/123 |
|  | 2-Hexenoic acid, 6-cyclohexyl- | 14.42 | 196.1464 | 196 | C12H20O2 | 81/96/67/196/109 |
|  | 2H-Octahydropyrido[1,2-a]pyrazin-1-one | 15.27 | 154.1107 | 154 | C8H14N2O | 125/97/154/126/83 |
|  | 2H-Pyran-2-carboxylic acid, 6-butoxy-3,6-dihydro-, butyl ester | 16.24 | 256.1675 | 256 | C14H24O4 | 155/41/57/183/99 |
|  | 2-Methyl-6-(1-propenyl)piperidine, [2R-[2.alpha., 6.alpha.(E)]- | 15.39 | 139.1362 | 139 | C9H17N | 124/96/68/82/41 |
|  | 2-Oxepanone, 7-hexyl- | 15.10 | 198.162 | 198 | C12H22O2 | 85/55/56/113/41 |
|  | 2-Trifluoroacetoxytetradecane | 10.34 | 310.212 | 310 | C16H29F3O2 | 41/69/83/97/111 |
|  | 3(2H)-Furanone, dihydro-5-isopropyl- | 23.70 | 128.0837 | 128 | C7H12O2 | 57/85/55/41/128 |
|  | 3-Amino-1-azabicyclo[2.2.2]octane | 11.55 | 126.1158 | 126 | C7H14N2 | 70/56/42/126/96 |
|  | 3-Aziridinopropionaldehyde carbethoxyhydrazone | 15.50 | 185.1165 | 185 | C8H15N3O2 | 43/99/56/113/70 |
|  | 3-Hepten-2-one, O-methyloxime | 14.53 | 141.1154 | 141 | C8H15NO | 41/141/82/95/39 |
|  | 4-(2,6-Dimethylcyclohexyl)butan-2-one | 15.40 | 182.1671 | 182 | C12H22O | 69/109/149/82/95 |
|  | 4-Decenoic acid, methyl ester | 16.23 | 184.1464 | 184 | C11H20O2 | 74/41/69/110/69 |
|  | 5-Ethyl-2-decen-4-one | 12.83 | 182.1671 | 182 | C12H22O | 69/97/112/41/43 |
|  | 6-Hydroxyhexahydrocyclopenta[b]furan-2-one | 14.90 | 142.0629 | 142 | C7H10O3 | 41/57/82/43/85 |
|  | 6-Methyl-5-oxo-11-propenyl-12,13-dioxa-tricyclo[7.3.1.0(1,6)]tridecane-8-carboxylic acid | 16.29 | 294.1467 | 294 | C16H22O5 | 93/41/138/55/69 |
|  | 7-Hydroxy-6,9a-dimethyl-3-methylene-decahydro-azuleno[4,5-b]furan-2,9-dione | 15.82 | 264.1361 | 264 | C15H20O4 | 41/55/123/95/67 |
|  | 7-Octadecanone | 15.33 | 268.2767 | 268 | C18H36O | 43/41/58/71/113 |
|  | 7-Oxabicyclo[4.1.0]heptane, 1,5-dimethyl- | 15.27 | 126.1045 | 126 | C8H14O | 43/71/41/68/95 |
|  | 8-Amino-1,3,6-triazahomoadamantane | 23.73 | 168.1376 | 168 | C8H16N4 | 168/85/57/42/83 |
|  | 8-Azabicyclo[3.2.1]octane, 8-acetyl- | 13.19 | 153.1154 | 153 | C9H15NO | 83/110/153/43/68 |
|  | 8-Hydroxy-2,2,8-trimethyldeca-5,9-dien-3-one | 15.37 | 210.162 | 210 | C13H22O2 | 57/71/85/41/107 |
|  | 9-Oxabicyclo[3.3.1]nonan-2-ol, acetate | 16.15 | 184.1099 | 184 | C10H16O3 | 43/124/81/41/95 |
|  | 9-Undecen-2-one, 6,10-dimethyl- | 19.09 | 196.1828 | 196 | C13H24O | 43/69/41/71/95 |
|  | Androst-5-en-17-one, 3-methoxy-16,16-dimethyl-, (3.alpha.)- | 19.02 | 330.256 | 330 | C22H34O2 | 41/55/81/91/105 |
|  | Bicyclo[2.1.1]hexane-1-carboxylic acid, 5,5-dimethyl- | 15.52 | 154.0994 | 154 | C9H14O2 | 41/69/43/95/109 |
|  | Bicyclo[3.2.0]heptane-2,6-diol, 5-(2-hydroxyethyl)-3,3-dimethyl-6-vinyl-, (Z)- | 16.36 | 226.1569 | 226 | C13H22O3 | 139/95/111/55/82 |
|  | Bromoacetic acid, pentadecyl ester | 15.53 | 348.1664 | 348 | C17H33BrO2 | 43/57/83/41/97 |
|  | Crotonic acid, menthyl ester | 13.66 | 224.1777 | 224 | C14H24O2 | 83/41/55/69/95 |
|  | Cyclohexane, (1-propylheptadecyl)- | 15.33 | 364.4071 | 364 | C26H52 | 55/83/41/57/97 |
|  | Cyclohexanecarboxylic acid, 2-ethylcyclohexyl ester | 15.85 | 238.1933 | 238 | C15H26O2 | 83/110/55/41/69 |
|  | Cyclohexanemethanol, 5-t-butyl-2-hydroxy- | 14.90 | 186.162 | 186 | C11H22O2 | 57/94/111/81/41 |
|  | Cyclohexanol, 1-(1-hexenyl)-, (E)- | 14.36 | 182.1671 | 182 | C12H22O | 55/139/41/83/125 |
|  | Cyclohexanone, 2-ethyl-, oxime | 14.79 | 141.1154 | 141 | C8H15NO | 113/41/55/82/27 |
|  | Cyclohexanone, 3-(hydroxymethyl)-, p-toluenesulfonate | 15.48 | 282.0926 | 282 | C14H18O4S | 110/91/82/67/41 |
|  | Cyclononane, 1,1,4,4,7,7-hexamethyl- | 14.63 | 210.2349 | 210 | C15H30 | 71/85.69/140/55 |
|  | Cyclopentane-trans-1,2-dicarboxamide | 12.64 | 156.0899 | 156 | C7H12N2O2 | 44/85/112/69/139 |
|  | Decane, 3,6-dimethyl- | 13.06 | 170.2035 | 170 | C12H26 | 57/43/71/85/41 |
|  | Decane, 4-cyclohexyl- | 15.99 | 224.2505 | 224 | C16H32 | 83/55/57/41/97 |
|  | Decanoic acid, 5,5-dimethyl-9-oxo-, methyl ester | 18.62 | 228.1726 | 228 | C13H24O3 | 43/69/109/171/41 |
|  | Dodecane, 1-isocyanato- | 16.14 | 211.1937 | 211 | C13H25NO | 99/41/43/56/112 |
|  | Eicosane, 7-hexyl- | 17.12 | 366.4228 | 366 | C26H54 | 57/43/71/41/85 |
|  | Eicosyl pentafluoropropionate | 14.67 | 444.3028 | 444 | C23H41F5O2 | 57/97/55/83/111 |
|  | Ethanone, 1-[1,2,5,6-tetrahydro-5,6-di(4-chlorophenyl)-2-methyl-1,2,4,5-tetrazin-3-yl]- | 14.42 | 362.0703 | 362 | C17H16Cl2N4O | 43/111/140/126/75 |
|  | Ethanone, 1-[2-ethyl-4-methyl-5-(1-methylethyl)-1,3,2-dioxaborolan-4-yl]- | 15.24 | 197.9444 | 198 | C10H19BO3 | 43/155/113/126/111 |
|  | Ethyl decyne carbonate | 17.12 | 210.162 | 210 | C13H22O2 | 55/81/41/67/165 |
|  | Fumaric acid, cycloheptyl hexadecyl ester | 15.54 | 436.3554 | 436 | C27H48O4 | 97/55/100/69/83 |
|  | Fumaric acid, dodecyl hex-4-yn-3-yl ester | 16.21 | 364.2614 | 364 | C22H36O4 | 80/69/97/55/41 |
|  | Isopulegol | 14.39 | 154.1358 | 154 | C10H18O | 41/69/55/84/121 |
|  | Methanesulfonic acid, 2,7-dioxatricyclo[4.3.1.0(3,8)]dec-5-yl ester | 16.25 | 234.0562 | 234 | C9H14O5S | 55/83/137/111/67 |
|  | N-(5-Methyl-isoxazol-3-yl)-3-(4-methyl-piperazin-1-yl)-propionamide | 15.92 | 252.1588 | 252 | C12H20N4O2 | 42/70/113/58/111 |
|  | N-Methyl-3-piperidinecarboxamide | 11.54 | 142.1107 | 142 | C7H14N2O | 84/57/55/83/110 |
|  | Nonadecyl heptafluorobutyrate | 17.14 | 480.2839 | 480 | C23H39F7O2 | 57/97/83/55/111 |
|  | Nonane, 3-methyl-5-propyl- | 10.73 | 184.2192 | 184 | C13H28 | 57/71/43/85/41 |
|  | Nonyl heptafluorobutyrate | 13.32 | 340.1273 | 340 | C13H19F7O2 | 55/69/70/41/97 |
|  | Octane, 3-ethyl-2,7-dimethyl- | 10.38 | 170.2035 | 170 | C12H26 | 43/57/71/41/85 |
|  | Octatriacontyl pentafluoropropionate | 15.26 | 696.5847 | 696 | C41H77F5O2 | 57/71/43/97/111 |
|  | Oxalic acid, 2-ethylhexyl octadecyl ester | 15.17 | 454.4024 | 454 | C28H54O4 | 57/71/43/112/41 |
|  | Oxalic acid, dodecyl propyl ester | 14.21 | 300.2301 | 300 | C17H32O4 | 43/57/41/71/85 |
|  | Oxalic acid, heptadecyl hexyl ester | 10.45 | 412.3554 | 412 | C25H48O4 | 43/57/85/41/71 |
|  | Oxane-4-carboxamide, 2-propyl- | 13.85 | 171.126 | 171 | C9H17NO2 | 85/128/55/72/98 |
|  | Pentanal, 2-methylene-, 2-(1-methylethyl)hydrazone | 14.68 | 154.1471 | 154 | C9H18N2 | 111/55/28/69/154 |
|  | Pentylamine, N-acetyl-1-cyano- | 13.06 | 154.1107 | 154 | C8H14N2O | 43/98/56/86/41 |
|  | Phenol, 3-fluoro-4-nitro- | 14.66 | 157.0175 | 157 | C6H4FNO3 | 127/57/83/157/99 |
|  | Piperazine, 1,4-diethyl- | 13.65 | 142.1471 | 142 | C8H18N2 | 57/142/42/28/86 |
|  | Piperazine, 1-[5-fluoropentyl]-4-[(3,4-dichlorophenyl)acetyl]- | 15.46 | 360.1173 | 360 | C17H23Cl2FN2O | 43/57/85/71/44 |
|  | p-Menthan-3-one, semicarbazone, (1R,4R)- | 11.56 | 211.1686 | 211 | C11H21N3O | 169/41/81/95/125 |
|  | Propanamide, 3-chloro-N-(4-chlorophenyl)-2-ethylsulfonyl-2-methyl- | 14.51 | 323.0151 | 323 | C12H15Cl2NO3S | 41/69/153/77/42 |
|  | Pyrimidin-2-one, 4-[N-methylureido]-1-[4-methylaminocarbonyloxymethyl | 16.11 | 325.1387 | 325 | C13H19N5O5 | 57/44/83/158/42 |
|  | Pyrrolidine, 1-(1-oxobutyl)- | 14.70 | 141.1154 | 141 | C8H15NO | 43/70/113/41/141 |
|  | Succinic acid, dodec-9-yn-1-yl dodecyl ester | 16.61 | 450.3711 | 450 | C28H50O4 | 101/68/95/55/82 |
|  | Pyrimidine-2,4,6(1H,3H,5H)-trione, 5-(4-methylpentylaminomethylene)- | 15.28 | 239.1271 | 239 | C11H17N3O3 | 168/125/196/239/41 |
|  | Sulfurous acid, 2-ethylhexyl octadecyl ester | 14.28 | 446.3796 | 446 | C26H54O3S | 57/71/43/113/41 |
|  | Sulfurous acid, hexyl undecyl ester | 12.42 | 320.2386 | 320 | C17H36O3S | 85/43/57/71/41 |
|  | Sulfurous acid, pentyl tridecyl ester | 15.75 | 334.2543 | 334 | C18H38O3S | 71/43/57/85/41 |
|  | Trichloroacetic acid, tetradecyl ester | 16.63 | 358.1235 | 358 | C16H29Cl3O2 | 43/57/83/41/97 |
|  | Tricosyl pentafluoropropionate | 14.85 | 486.3498 | 486 | C26H47F5O2 | 57/43/97/83/111 |
|  | Z-(13,14-Epoxy)tetradec-11-en-1-ol acetate | 16.17 | 268.2039 | 268 | C16H28O3 | 43/97/69/41/82 |
|  | Z-6,17-Octadecadien-1-ol acetate | 14.42 | 308.2716 | 308 | C20H36O2 | 43/67/41/82/95 |
| BG-70 | 1-(6-Methyl-2-piperidyl)propan-2-one | 9.09 | 155.1311 | 155 | C9H17NO | 69/43/98/97/82 |
|  | 1,2,4-Oxadiazole, 5-methyl-3-(1-piperidylmethyl)- | 21.51 | 181.1216 | 181 | C9H15N3O | 98/84/41/69/83 |
|  | 2-Cyclohexyl-1-tetrazol-2-yl-ethanone | 13.82 | 194.1169 | 194 | C9H14N4O | 55/113/41/97/82 |
|  | 2-Propanamine, N-(2,2-dimethylpropylidene)-2-methyl- | 11.65 | 141.1518 | 141 | C9H19N | 57/126/41/141/70 |
|  | 2-Tetradecene, (E)- | 9.10 | 196.2192 | 196 | C14H28 | 55/41/56/70/97 |
|  | 3-Chloropropionic acid, 2-pentadecyl ester | 15.16 | 318.2327 | 318 | C18H35ClO2 | 41/91/57/27/83 |
|  | 3-Pyridinecarboxylic acid, 1,4,5,6-tetrahydro-1,2-dimethyl-6-oxo-, ethyl ester | 15.05 | 197.1052 | 197 | C10H15NO3 | 56/124/152/44/82 |
|  | 4,9-Decadienoic acid, 2-nitro-, ethyl ester | 14.90 | 241.1314 | 241 | C12H19NO4 | 29/67/55/79/27 |
|  | 5,9(1H,6H)-Benzocyclooctenedione, octahydro-7,7-dimethyl- | 25.21 | 218.1784 | 222 | C14H22O2 | 83/141/112/222/41 |
|  | Benzoic acid, 4-chloro-, (1-methyl-4-piperidinylidenamino) ester | 13.65 | 266.0823 | 266 | C13H15ClN2O2 | 139/110/141/57/70 |
|  | Cyclododecene | 23.55 | 166.1722 | 166 | C12H22 | 67/55/82/41/96 |
|  | Ethanone, 1-(5-ethenyl-2-ethyl-4-methyl-1,3,2-dioxaborolan-4-yl)- | 25.28 | 181.9131 | 182 | C9H15BO3 | 43/139/83/27/111 |
|  | Ether, methyl 1-tetradecenyl | 15.25 | 226.2298 | 226 | C15H30O | 71/41/43/68/96 |
|  | Malonic acid, 2,4-dimethylpent-3-yl heptadecyl ester | 15.88 | 440.3867 | 440 | C27H52O4 | 57/105/83/325/43 |
|  | Muscone | 15.26 | 238.2298 | 238 | C16H30O | 41/55/85/69/71 |
|  | Nonanal | 24.05 | 142.1358 | 142 | C9H18O | 57/41/70/98/27 |
|  | Octane, 2-iodo- | 10.52 | 240.0376 | 240 | C8H17I | 57/71/41/113/27 |
|  | Sulfurous acid, hexyl tetradecyl ester | 15.10 | 362.2856 | 362 | C20H42O3S | 85/43/57/71/41 |
| BG-100GC | (R-(R*,R*))-4-(1,5-Dimethylhexyl)-1-cyclohexenecarboxylic acid | 16.58 | 238.1933 | 238 | C15H26O2 | 154/57/126/43/125 |
|  | 1-(4-Fluorophenyl)-2-methyl-2-propanol | 17.78 | 168.095 | 168 | C10H13FO | 59/110/109/89 |
|  | 1-(Dodecyloxy)-2-nitrobenzene | 18.06 | 326.2132 | 307 | C18H29NO3 | 57/43/71/41/85 |
|  | 1,2-Cyclohexanedicarboxaldehyde | 15.41 | 140.0837 | 140 | C8H12O2 | 81/41/55/112/67 |
|  | 1,3-Cyclopentanedione, 4-hydroxy-5-(3-methylbutyl)- | 25.27 | 184.1099 | 184 | C10H16O3 | 57/96/81/43/41 |
|  | 1,3-Dimethyl-4-(1-phenylethylamino)piperidine | 15.49 | 232.1941 | 232 | C15H24N2 | 96/111/127/70/113 |
|  | 1-Pentanol, 3-methyl-2-propyl- | 7.84 | 144.1515 | 144 | C9H20O | 57/71/43/41/113 |
|  | 2- Chloropropionic acid, decyl ester | 14.84 | 248.1544 | 248 | C13H25ClO2 | 43/57/41/70/85 |
|  | 2-(6-Isopropyl-3-methylcyclohex-1-enyloxymethyl)pent-4-en-1-ol | 16.20 | 252.209 | 252 | C16H28O2 | 41/139/81/43/69 |
|  | 2,2,3,3-Tetramethylcyclopropanecarboxylic acid, 1-(2-propyl)butyl ester | 25.25 | 240.209 | 240 | C15H28O2 | 57/97/125/127/55 |
|  | 2,3:5,6-Di-O-1-Cyclohexylieden-1,4-cyclohexa | 15.46 | 420.2512 | 420 | C24H36O6 | 41/55/81/113/69 |
|  | 2,6-Dioxatricyclo[3.3.2.0(3,7)]decan-9-ol | 15.43 | 156.0786 | 156 | C8H12O3 | 55/68/156/83/84 |
|  | 2-Carboxymethyl-3-methyl-cyclopentanecarboxylic acid | 23.68 | 186.0892 | 186 | C9H14O4 | 81/140/95/67/27 |
|  | 2-Exo-hydroxy-5-ketobornane | 14.72 | 168.115 | 168 | C10H16O2 | 70/41/109/55/69 |
|  | 2-Methyl-cis-7,8-epoxynonadecane | 25.26 | 296.308 | 296 | C20H40O | 43/57/82/41/97 |
|  | 2-Octyldecyl acetate | 25.21 | 312.303 | 312 | C20H40O2 | 43/57/83/97/41 |
|  | 2-Oxepanone, 7-butyl- | 16.91 | 170.1307 | 170 | C10H18O2 | 55/85/56/41/113 |
|  | 2R,3s-1-[[1,3-Dihydroxy-2-butoxy]methyl]-1,2,4-triazole-3-carboxamide | 17.40 | 230.1016 | 230 | C8H14N4O4 | 125/45/41/82/140 |
|  | 2-Undecanethiol, 2-methyl- | 10.44 | 202.1756 | 202 | C12H26S | 41/55/69/83/84 |
|  | 3-Acetonylcycloheptanone | 25.18 | 168.115 | 168 | C10H16O2 | 43/111/83/41/58 |
|  | 3-Chloropropionic acid, 2-tetradecyl ester | 16.74 | 304.217 | 304 | C17H33ClO2 | 41/91/57/27/70 |
|  | 3-Hexen-2-one, 3,4-dimethyl- | 11.57 | 126.1045 | 126 | C8H14O | 43/41/126/83/111 |
|  | 5-Aminovaleramide, N-methyl-N-[4-(1-pyrrolidinyl]-2-butynyl]- | 16.90 | 251.1999 | 251 | C14H25N3O | 42/121/70/44/56 |
|  | 6-Bromohexanoic acid, 2-tetradecyl ester | 13.05 | 390.2134 | 390 | C20H39BrO2 | 41/43/69/177/97 |
|  | 8-Heptadecene, 9-octyl- | 16.28 | 350.3915 | 350 | C25H50 | 55/83/41/97/154 |
|  | 9-Borabicyclo[3.3.1]nonan-9-ol, 9-(2,2-dimethyl-3-pentyl)- ether | 16.48 | 236.0329 | 236 | C15H29BO | 138/57/98/41/43 |
|  | 9-Undecenal, 2,10-dimethyl- | 25.24 | 196.1828 | 196 | C13H24O | 43/69/41/82/95 |
|  | Acetamide, N-(2-piperidin-4-ylethyl)- | 13.15 | 170.142 | 170 | C9H18N2O | 85/43/98/56/82 |
|  | Acetic acid, chloro-, hexadecyl ester | 14.84 | 318.2327 | 318 | C18H35ClO2 | 69/83/55/97/41 |
|  | Acetic acid, trifluoro-, dodecyl ester | 10.44 | 282.1807 | 282 | C14H25F3O2 | 55/69/41/83/97 |
|  | Bicyclo[6.3.0]undecane-2,6-dione | 16.15 | 180.115 | 180 | C11H16O2 | 95/124/67/83/41 |
|  | Bromoacetic acid, dodecyl ester | 14.71 | 306.1195 | 306 | C14H27BrO2 | 55/41/69/83/97 |
|  | Chloromethyl 7-chlorodecanoate | 18.98 | 254.0842 | 254 | C11H20Cl2O2 | 55/69/41/84/110 |
|  | cis-1-Chloro-9-octadecene | 18.52 | 286.2429 | 286 | C18H35Cl | 83/69/55/97/41 |
|  | Cycloheptanone, 4-acetyl-7,7-dimethyl-2-(2-oxopropyl)- | 16.00 | 238.1569 | 238 | C14H22O3 | 43/71/111/181/85 |
|  | Cyclooctacosane | 12.73 | 392.4384 | 392 | C28H56 | 57/43/83/97/41 |
|  | Cyclooctyl alcohol | 14.65 | 128.1201 | 128 | C8H16O | 57/41/82/55/27 |
|  | Ditetradecyl ether | 14.14 | 410.449 | 410 | C28H58O | 57/71/43/197/85 |
|  | Formic acid, undecyl ester | 7.94 | 200.1777 | 200 | C12H24O2 | 83/55/69/72 |
|  | Hexadecane, 5-butyl- | 17.12 | 282.3288 | 282 | C20H42 | 43/57/71/41/85 |
|  | Hexanoic acid, 5,5-dimethyl-2,4-dioxo-, ethyl ester | 15.11 | 200.1048 | 200 | C10H16O4 | 127/43/57/29/115 |
|  | Myristic acid vinyl ester | 14.28 | 254.2247 | 254 | C16H30O2 | 57/43/71/211/41 |
|  | Oxalic acid, 6-ethyloct-3-yl propyl ester | 15.04 | 272.1988 | 272 | C15H28O4 | 43/85/57/71/41 |
|  | Phosphorous acid, tris(decyl) ester | 16.40 | 502.4517 | 502 | C30H63O3P | 83/141/57/85/43 |
|  | Piperidine, 1-(1-oxo-2-butenyl)- | 16.10 | 153.1154 | 153 | C9H15NO | 138/69/41/84/42 |
|  | Propanoic acid, 2-methyl-, dodecyl ester | 16.33 | 256.2403 | 256 | C16H32O2 | 43/89/71/57/41 |
|  | Pyrrolidine, 1-(1,6-dioxooctadecyl)- | 14.21 | 351.3139 | 351 | C22H41NO2 | 43/57/41/83/113 |
|  | Tetradecane, 5-methyl- | 13.31 | 212.2505 | 212 | C15H32 | 43/57/85/71/41 |
|  | trans-2-Ethyl-2-hexen-1-ol | 9.08 | 128.1201 | 128 | C8H16O | 41/27/55/95/81 |
|  | Trichloroacetic acid, undecyl ester | 14.41 | 316.0765 | 316 | C13H23Cl3O2 | 57/83/43/97/41 |
|  | Tritetracontane | 15.74 | 604.689 | 604 | C43H88 | 57/71/43/85/41 |
| DSI | (E)-4-Chloro-3-methyl-1,3-hexadiene | 12.09 | 130.055 | 130 | C7H11Cl | 95/130/67/79/115 |
|  | 1,1'-Bicyclohexyl, 2-propyl-, cis- | 15.39 | 208.2192 | 208 | C15H28 | 69/41/83/55/125 |
|  | 1,1-Ethanediol, diacetate | 7.15 | 146.0579 | 146 | C6H10O4 | 43/87/15/29/103 |
|  | 1,2-Epoxyundecane | 8.05 | 170.1671 | 170 | C11H22O | 71/41/56/27/96 |
|  | 1,2-Ethanediamine, N,N'-bis[1-(tetrahydrofuran-2,4-dion-3-ylidene)ethyl]- | 15.07 | 308.1008 | 308 | C14H16N2O6 | 30/67/167/42/109 |
|  | 1,3-Benzenediol, 4,6-dichloro- | 6.69 | 177.9589 | 178 | C6H4Cl2O2 | 178/51/86/115/69 |
|  | 11-Butyldocosane | 12.85 | 366.4228 | 366 | C26H54 | 57/43/71/85/41 |
|  | 11-Tridecen-1-ol | 10.34 | 198.1984 | 198 | C13H26O | 43/41/57/82/96 |
|  | 1-Cyclohexene-1-acetaldehyde, 2,6,6-trimethyl | 12.82 | 166.1358 | 166 | C11H18O | 151/107/81/95/123 |
|  | 1-Dodecanol, 2-methyl-, (S)- | 8.26 | 200.2141 | 200 | C13H28O | 57/41/55/71/97 |
|  | 1-Heptadecen-7,10-dione | 15.38 | 266.2247 | 266 | C17H30O2 | 55/127/114/83/41 |
|  | 1-Propene, 2-methyl-3-(1-methylethoxy)- | 7.35 | 114.1045 | 114 | C7H14O | 55/72/27/41/114 |
|  | 1-Propene, 3,3,3-trichloro- | 7.32 | 143.9301 | 144 | C3H3Cl3 | 109/38/83/49/113 |
|  | 2-(Diethylamino)acetonitrile | 5.56 | 112.1001 | 112 | C6H12N2 | 42/97/69/27/112 |
|  | 2,2-Difluoroheptacosanoic acid | 14.34 | 264.1901 | 264 | C14H26F2O2 | 43/57/41/71/151 |
|  | 2-Decanol, pentafluoropropionate | 14.67 | 304.1462 | 304 | C13H21F5O2 | 69/41/70/119/97 |
|  | 2-Heptanone, O-methyloxime | 9.21 | 143.1311 | 143 | C8H17NO | 42/87/41/100/57 |
|  | 2-methyltetracosane | 18.93 | 352.4071 | 352 | C25H52 | 57/43/71/85/41 |
|  | 2-Nonenal | 10.91 | 140.1201 | 140 | C9H16O | 41/70/27/57/84 |
|  | 2-Propanol, 1,1,1-trichloro- | 5.83 | 161.9407 | 162 | C3H5Cl3O | 45/27/63/83/111 |
|  | 2-Propyl-1-heptanol | 13.09 | 158.1671 | 158 | C10H22O | 43/57/71/41/85 |
|  | 3-(Dimethylamino)-1-(2-furyl)prop-2-en-1-one | 13.88 | 165.079 | 165 | C9H11NO2 | 42/136/165/95/148 |
|  | 3,3-Dimethylbutan-2-yl propyl carbonate | 14.28 | 188.1413 | 188 | C10H20O3 | 43/85/57/132/41 |
|  | 3,5-Cyclohexadiene-1,2-dione, 3,5-bis(1,1-dimethylethyl)- | 16.69 | 220.1464 | 220 | C14H20O2 | 57/205/41/149/108 |
|  | 3,7-Dimethyldecane | 10.57 | 170.2035 | 170 | C12H26 | 43/57/71/41/85 |
|  | 3-[p-Chlorophenyl]-5-[trichloromethyl]-1,2,4-oxadiazole | 13.81 | 295.908 | 296 | C9H4Cl4N2O | 75/50/111/153/261 |
|  | 3-cis-Methoxy-5-cis-methyl-1R-cyclohexanol | 7.92 | 144.115 | 144 | C8H16O2 | 87/41/58/43/39 |
|  | 3-Trifluoroacetoxydodecane | 6.31 | 282.1807 | 282 | C14H25F3O2 | 41/57/70/97/98 |
|  | 4-Hydroxy-4-methylhex-5-enoic acid | 12.41 | 200.1413 | 200 | C11H20O3 | 57/41/71/27/111 |
|  | 4-Trifluoroacetoxytetradecane | 11.44 | 310.212 | 310 | C16H29F3O2 | 55/44/69/83/97 |
|  | 5-Hexyn-3-ol | 7.00 | 98.0732 | 98 | C6H10O | 59/31/27/43/14 |
|  | 5-Methyl-4,5-dihydroisoxazole-5-carboxylic acid, methyl ester | 7.82 | 143.0582 | 143 | C6H9NO3 | 43/84/41/83/59 |
|  | 6-propyltridecane | 10.50 | 226.2662 | 226 | C16H34 | 57/43/71/41/85 |
|  | 7,9-Di-tert-butyl-1-oxaspiro(4,5)deca-6,9-diene-2,8-dione | 17.41 | 276.1726 | 276 | C17H24O3 | 57/205/55/175/217 |
|  | 7-Hexyltridecan-1-ol | 15.41 | 284.308 | 284 | C19H40O | 69/83/111/97/55 |
|  | 7-Propyltridecane | 11.49 | 226.2662 | 226 | C16H34 | 43/57/71/85/41 |
|  | 8-Heptadecene | 9.04 | 350.3915 | 350 | C25H50 | 55/83/41/97/154 |
|  | 9-Bromononaldehyde | 19.59 | 220.0463 | 220 | C9H17BrO | 55/41/57/97/73 |
|  | 9-Octadecenal, (Z)- | 15.92 | 266.2611 | 266 | C18H34O | 55/41/69/83/95 |
|  | 9-Octadecenoic acid (Z)-, tetradecyl ester | 13.76 | 478.4752 | 478 | C32H62O2 | 55/264/83/97/41 |
|  | Acetic acid, 5-acetoxy-4-nitrotetrahydropyran-3-yl ester | 8.93 | 247.0691 | 247 | C9H13NO7 | 43/81/69/159/41 |
|  | Acetic acid, trichloro-, anhydride | 5.16 | 305.7981 | 306 | C4Cl6O3 | 117/44/82/84/63 |
|  | Aclarubicin | 8.08 | 811.3415 | 811 | C42H53NO15 | 113/100/71/43/57 |
|  | Allyl nonanoate | 15.48 | 198.162 | 198 | C12H22O2 | 41/43/57/71/141 |
|  | Bis[3,4-dichlorophenyl]sulfone | 14.06 | 353.8844 | 354 | C12H6Cl4O2S | 193/356/145/109/75 |
|  | Butyrylthioacetic acid, ethyl ester | 9.45 | 190.0664 | 190 | C8H14O3S | 71/43/27/117/88 |
|  | Carbonic acid, decyl 2,2,2-trichloroethyl ester | 9.98 | 332.0714 | 332 | C13H23Cl3O3 | 55/69/97/41/112 |
|  | Cetene | 13.53 | 224.2505 | 224 | C16H32 | 41/57/83/97/111 |
|  | Cyclohexanol, 2-(2-propynyloxy)-, trans- | 13.80 | 154.0994 | 154 | C9H14O2 | 41/82/27/67/55 |
|  | Cyclohexanol, 2,4-dimethyl- | 8.13 | 128.1201 | 128 | C8H16O | 95/57/41/71/27 |
|  | Cyclopentanecarboxamide, N-allyl- | 14.35 | 153.1154 | 153 | C9H15NO | 69/112/41/97/153 |
|  | Cyclopropaneundecanal, 2-nonyl- | 12.15 | 336.3394 | 336 | C23H44O | 83/97/69/98/223 |
|  | Dichloroacetic acid | 9.16 | 254.0842 | 254 | C11H20Cl2O2 | 43/56/70/41/85 |
|  | Docosane, 2,4-dimethyl- | 14.08 | 338.3915 | 338 | C24H50 | 43/85/57/71/41 |
|  | Dodecane, 5-cyclohexyl- | 15.32 | 252.2818 | 252 | C18H36 | 55/83/41/97/111 |
|  | Dodecanoic acid, 1-methylethyl ester | 13.86 | 242.2247 | 242 | C15H30O2 | 43/60/41/102/73 |
|  | Ethanone, 1-(4-amino-6-methyl-thieno[2,3-b]pyridin5-yl)- | 12.83 | 206.0515 | 206 | C10H10N2OS | 191/206/163/136/43 |
|  | Ethyl 9-hexadecenoate | 10.99 | 282.256 | 282 | C18H34O2 | 55/88/69/41/83/101 |
|  | Ethyl dodecyl ether | 9.25 | 214.2298 | 214 | C14H30O | 59/41/83/97/168 |
|  | Furazan-3,4-diamine, N,N'-dimethyl-N,N'-dinitro- | 13.63 | 214.2298 | 218 | C4H6N6O5 | 42/96/67/71/172 |
|  | Hept-5-one-1-lic acid, 4,4-dimethyl-, ethyl ester | 10.96 | 200.1413 | 200 | C11H20O3 | 57/70/41/97/115 |
|  | Heptane, 1-bromo-6-methyl- | 18.27 | 192.0514 | 192 | C8H17Br | 41/69/149/27/97 |
|  | Hexadecanoic acid, methyl ester | 17.60 | 270.256 | 270 | C17H34O2 | 74/87/43/41/143 |
|  | Hexane, 1,6-diisocyanato- | 15.80 | 168.0899 | 168 | C8H12N2O2 | 41/56/43/85/99 |
|  | Hexanoic acid, 2-ethyl-, tetradecyl ester | 10.90 | 340.3343 | 340 | C22H44O2 | 57/43/145/41/88 |
|  | Isopropyl myristate | 16.01 | 270.256 | 270 | C17H34O2 | 43/102/60/41/228 |
|  | Methyl 12-oxo-9-dodecenoate | 12.16 | 226.1569 | 226 | C13H22O3 | 55/41/74/98/69 |
|  | Methyl 4-chloro-2,2-dimethyl-4-pentenoate | 5.29 | 176.0604 | 176 | C8H13ClO2 | 141/41/73/15/117 |
|  | N-(5-Chloro-2-hydroxyphenyl)dodecanamide | 13.71 | 325.181 | 325 | C18H28ClNO2 | 41/57/143/71/85 |
|  | o-Acetyl-L-serine | 15.04 | 147.0531 | 147 | C5H9NO4 | 43/60/102/74/87 |
|  | Octadecane, 1,1'-[(1-methyl-1,2-ethanediyl)bis(oxy)]bis- | 14.14 | 580.6161 | 580 | C39H80O2 | 57/43/71 |
|  | Octadecane, 3-ethyl-5-(2-ethylbutyl) | 13.22 | 366.4228 | 366 | C26H54 | 43/57/71/85/99 |
|  | Oxalic acid | 15.00 | 314.2458 | 314 | C18H34O4 | 43/85/57/71/41 |
|  | Pentanesulfonic acid, 5,5,5-trichloro-, 4-nitrophenyl ester | 7.86 | 374.9503 | 375 | C11H12Cl3NO5S | 139/41/109/65/75 |
|  | Phosphirane, 1-menthyl-2-vinyl- | 10.94 | 224.1695 | 224 | C14H25P | 41/87/55/81/69 |
|  | Phthalic acid, 4,4-dimethylpent-2-yl isobutyl ester | 18.30 | 320.1988 | 320 | C19H28O4 | 149/57/83/223/41 |
|  | Propane-1,1-diol diacetate | 8.21 | 160.0735 | 160 | C7H12O4 | 43/101/57/29/15 |
|  | Propanoic acid, 2,2,2-trichloro-1-methylethyl ester | 8.31 | 217.9669 | 218 | C6H9Cl3O2 | 57/109/75/145/35 |
|  | Propanoic acid, 3-(perhydro-2,5-dioxo-imidazo[4,5-d]imidazol-1-yl)- | 19.03 | 214.0703 | 214 | C7H10N4O4 | 43/84/99/40/112 |
|  | Pyridine, 2-chloro-, 1-oxide | 6.46 | 128.9982 | 129 | C5H4ClNO | 129/39/78/113/51 |
|  | Sulfurous acid, hexyl octyl ester | 23.68 | 278.1917 | 278 | C14H30O3S | 57/85/43/71/41 |
|  | trans-4,cis-6-Dimethyl-7-oxo-8-oxabicyclo(2.2.2)octane-2-carboxylic acid | 19.04 | 198.0892 | 198 | C10H14O4 | 43/108/138/113/82 |
|  | Tridecanedial | 13.75 | 212.1777 | 212 | C13H24O2 | 55/95/67/41/81 |
| ESC | 1,1-Dichloronon-1-ene | 17.03 | 194.063 | 194 | C9H16Cl2 | 43/56/41/70/109 |
|  | 1,3,5-Triazin-2(1H)-one, 4,6-bis(ethylamino)- | 14.09 | 183.1121 | 183 | C7H13N5O | 44/183/69/155/140 |
|  | 1,3,5-Triazine-2,4-diamine, N,N'-diethyl-6-methoxy- | 15.13 | 197.1278 | 197 | C8H15N5O | 197/139/154/44/169 |
|  | 1,4-Naphthalenediol, decahydro-, (1.alpha.,4.beta.,4a.alpha.,8a.alpha.)- | 17.33 | 170.1307 | 170 | C10H18O2 | 152/134/108/67/95 |
|  | 10-Methyldodecan-4-olide | 15.91 | 212.1777 | 212 | C13H24O2 | 85/70/41/55/57 |
|  | 1-Azacyclononan-2-one | 16.67 | 141.1154 | 141 | C8H15NO | 30/55/124/98/84 |
|  | 1-Bromo-3,7-dimethyloctane | 16.05 | 220.0827 | 220 | C10H21Br | 71/55/41/149/113 |
|  | 1-Butanone, 3-methyl-1-[2,3,5-trihydroxy-4-(3-methyl-2-butenyl)-1,4-cyclopentadien-1-yl]- | 13.64 | 266.1518 | 266 | C15H22O4 |  |
|  | 1-Cyclohexene-1-methanol, .alpha.,2,6,6-tetramethyl- | 25.28 | 168.1515 | 168 | C11H20O | 43/135/41/123/107 |
|  | 1-Heptadec-1-ynyl-cyclopentanol | 16.15 | 320.308 | 320 | C22H40O | 41/98/67/85/124 |
|  | 1-Iodo-2-methylundecane | 12.18 | 296.1002 | 296 | C12H25I | 57/43/71/41/85 |
|  | 1R,4s,7s,8R,11R-2,2,4,8-Tetramethyltricyclo[5.3.1.0(4,11)]undecan-7-ol | 16.29 | 222.1984 | 222 | C15H26O | 166/123/151/222/97 |
|  | 2,4-Hexanedione, 5-methyl-3-(2-methyl-1-propenyl)- | 15.81 | 182.1307 | 182 | C11H18O2 | 43/139/112/97/182 |
|  | 2-Amino-6-(tert-butylamino)-1,3,5-triazine | 15.39 | 167.1172 | 167 | C7H13N5 | 152/43/111/70/41 |
|  | 2-Cyclopenten-1-one, 2-butyl-3-methoxy- | 17.24 | 168.115 | 168 | C10H16O2 | 126/97/67/39/125 |
|  | 2-Decanone, 5,9-dimethyl- | 10.38 | 184.1828 | 184 | C12H24O | 43/71/58/41/126 |
|  | 2-Ethyl-2-hydroxy-1,3-dimethylcyclopentanecarboxylic acid, ethyl ester | 15.40 | 214.1569 | 214 | C12H22O3 | 57/140/112/29/158 |
|  | 2-Isopropyl-4-methylhex-2-enal | 16.61 | 154.1358 | 154 | C10H18O | 41/69/139/154/83 |
|  | 2-Octen-1-ol, 3,7-dimethyl- | 15.41 | 156.1515 | 156 | C10H20O | 69/41/55/81/95 |
|  | 2-Pentanone, 4-methyl-1-(2,3,4,5-tetrahydro-5-methyl[2,3'-bifuran]-5-yl)- | 17.40 | 250.1569 | 250 | C15H22O3 | 43/85/57/151/41 |
|  | 3,6,12-Trimethyl-1,4,7,10,13,16-hexaaza-cyclooctadecane-2,5,8,11,14,17-hexaone | 13.16 | 384.1759 | 384 | C15H24N6O6 | 42/56/27/128/85 |
|  | 3-Octen-2-one, 4-(methylamino)- | 25.22 | 155.1311 | 155 | C9H17NO | 71/113/43/98/140 |
|  | 4.Xi.-Germacr-9-en-12-oic acid, 6.alpha.-hydroxy-1-oxo-, .gamma.-lactone, (11S)- | 16.96 | 250.1569 | 250 | C15H22O3 | 95/55/41/82/122 |
|  | 4-Amino-4-cyano-1,2.5-trimethylpiperidine | 25.27 | 167.1424 | 167 | C9H17N3 | 70/42/58/152/125 |
|  | 4-Aminobutyramide, N-methyl-N-[4-(1-pyrrolidinyl)-2-butynyl]-N',N'-bis(trifluoroacetyl)- | 14.52 | 429.1488 | 429 | C17H21F6N3O3 | 44/69/121/70/41 |
|  | 4-Hydroxy-2,2,7,7-tetramethyl-octahydro-2H-dibenzofuran-1,8-dione | 15.82 | 280.1675 | 280 | C16H24O4 | 41/70/193/85/43 |
|  | 4-t-Butyl-1-(1-methylallyl)cyclohexanol | 13.06 | 210.1984 | 210 | C14H26O | 155/57/81/55/41 |
|  | 5,5,7,7-Tetraethylundecane | 10.73 | 268.3132 | 268 | C19H40 | 57/71/43/85/41 |
|  | 6-Hydroxy-9-oxa-bicyclo[3.3.1]nonan-3-one | 14.33 | 156.0786 | 156 | C8H12O3 | 69/43/41/97/113 |
|  | 7,8-Dioxabicyclo[3.2.1]oct-2-ene | 14.35 | 112.0524 | 112 | C6H8O2 | 81/112/39/53/84 |
|  | Bicyclo[3.1.1]heptane, 2,6,6-trimethyl-, [1R-(1.alpha.,2.beta.,5.alpha.)]- | 9.11 | 138.1409 | 138 | C10H18 | 41/55/67/95/81 |
|  | Cyclohexane, 1-(cyclohexylmethyl)-2-ethyl-, trans- | 25.24 | 208.2192 | 208 | C15H28 | 55/41/111/69/83 |
|  | Cyclohexane, 1,1'-(oxydi-2,1-ethanediyl)bis[4-methyl- | 15.47 | 266.2611 | 266 | C18H34O | 124/95/55/81/69 |
|  | Cyclohexanol, 5-methyl-2-(1-methylethenyl)- | 16.48 | 154.1358 | 154 | C10H18O | 41/67/81/55/93 |
|  | Cyclohexanone, 2-(3-oxobutyl)- | 15.41 | 168.115 | 168 | C10H16O2 | 43/111/83/97/168 |
|  | Cyclohexanone, 2,3-dimethyl-2-(3-oxobutyl)- | 13.75 | 196.1464 | 196 | C12H20O2 | 43/126/41/111/69 |
|  | Decane, 5-propyl- | 10.72 | 184.2192 | 184 | C13H28 | 57/71/43/85/41 |
|  | Dimenoxadol | 14.68 | 327.1835 | 327 | C20H25NO3 | 58/105/43/71/167 |
|  | Dodecane, 2-cyclohexyl- | 15.32 | 252.2818 | 252 | C18H36 | 82/55/57/41/111 |
|  | Dodecane, 4-cyclohexyl- | 16.91 | 252.2818 | 252 | C18H36 | 83/55/41/97/111 |
|  | E,E-3,13-Octadecadien-1-ol | 15.84 | 266.2611 | 266 | C18H34O | 55/41/81/96/67 |
|  | E-6-Octadecen-1-ol acetate | 15.48 | 310.2873 | 310 | C20H38O2 | 82/43/67/96/41 |
|  | Ergost-25-ene-3,5,6,12-tetrol, (3.beta.,5.alpha.,6.beta.,12.beta.)- | 15.86 | 448.3554 | 448 | C28H48O4 | 55/69/41/81/95 |
|  | Heptanoic acid, 5-hexen-1-yl ester | 16.80 | 212.1777 | 212 | C13H24O2 | 41/67/82/113/27 |
|  | Hexatriacontyl trifluoroacetate | 15.39 | 618.5565 | 618 | C38H73F3O2 | 57/71/97/43/111 |
|  | Histidine-2-carboxylic acid, N-t-butyloxycarbonyl, methyl(ester) | 16.13 | 313.1274 | 313 | C13H19N3O6 | 44/82/140/96/42 |
|  | N-Ethyl-hexahydro-1H-azepine | 14.67 | 127.1362 | 127 | C8H17N | 127/41/112/56/70 |
|  | N-methylene-n-decylimine | 16.71 | 169.1831 | 169 | C11H23N | 43/84/41/57/70 |
|  | N-Methylene-n-tetradecylamine | 15.49 | 225.2458 | 225 | C15H31N | 84/43/57/41/70 |
|  | Nonadecane | 17.11 | 296.3445 | 296 | C21H44 | 43/57/71/41/85 |
|  | Octadecane, 1-(ethenyloxy)- | 15.16 | 296.308 | 296 | C20H40O | 43/57/83/41/97 |
|  | Octadecyl trifluoroacetate | 12.73 | 366.2747 | 366 | C20H37F3O2 | 57/43/97/83/41 |
|  | Octatriacontyl trifluoroacetate | 15.54 | 646.5879 | 646 | C40H77F3O2 | 57/71/97/43/111 |
|  | Oxalic acid, hexadecyl isohexyl ester | 15.10 | 398.3397 | 398 | C24H46O4 | 43/85/57/41/71 |
|  | Pentane-2,4-dione, 3-amino(1-piperazino)methylene- | 14.72 | 211.1322 | 211 | C10H17N3O2 | 43/143/56/84/126 |
|  | Pregabalin | 14.73 | 159.126 | 159 | C8H17NO2 | 41/56/84/70/141 |
|  | Testosterone, oxime | 14.70 | 303.2199 | 303 | C19H29NO2 | 57/55/41/91/303 |
|  | Tricosyl heptafluorobutyrate | 14.85 | 536.3466 | 536 | C27H47F7O2 | 57/43/97/83/111 |
|  | Trivinyl(hydroxymethyl)silane | 14.12 | 140.1748 | 140 | C7H12OSi | 55/41/109/83/40 |
|  | Undecane, 3,8-dimethyl- | 12.86 | 184.2192 | 184 | C13H28 | 57/43/71/85/41 |
|  | Undecane, 4,7-dimethyl- | 11.49 | 184.2192 | 184 | C13H28 | 43/57/71/85/41 |
|  | Z,Z-6,28-Heptatriactontadien-2-one | 10.58 | 530.543 | 530 | C37H70O | 69/55/83/95/109 |
| KRI | 1,1,3,6-tetramethyl-2-(3,6,10,13,14-pentamethyl-3-ethyl-pentadecyl)cyclohexane | 15.42 | 448.5011 | 448 | C32H64 | 57/71/55/85/127 |
|  | 1-.beta.-d-Ribofuranosyl-1,2,4-triazole-3-carboxylic acid | 15.91 | 245.0648 | 245 | C8H11N3O6 | 44/69/42/70/112 |
|  | 1-Ethene,2-(2,2,5a-trimethyl-3-oxo-1a,2,3,5a-tetrahydro-1-benzoxiren-1-yl)-1-(acetoxy) | 13.78 | 236.1048 | 236 | C13H16O4 | 43/97/98/139/41 |
|  | 1-Iodoundecane | 10.50 | 282.0845 | 282 | C11H23I | 57/43/41/71/85 |
|  | 1-Methyl-2-pyrrolidone-4-carboxamide | 10.33 | 142.0743 | 142 | C6H10N2O2 | 41/98/70/97/68 |
|  | 1-Methyl-4-nitro-5-[(1,2-dimethyl-3-hydroxybutyl)amino]-(1H)-imidazole | 15.19 | 242.138 | 242 | C10H18N4O3 | 98/57/125/169/142 |
|  | 2-(1,5-Dimethyl-hexyl)-cyclobutanone | 12.82 | 182.1671 | 182 | C12H22O | 69/41/55/70/112 |
|  | 3,3-Dimethyl-1,5-dioxa-10,17-diazacycloheneic | 14.67 | 384.2261 | 384 | C19H32N2O6 | 97/69/168/55/113 |
|  | 3-Cyclopentylpropionic acid, 6-ethyl-3-octyl ester | 15.52 | 282.256 | 282 | C18H34O2 | 125/55/84/69/41 |
|  | 4,4'-(Methylenedi-4,1-phenylene)bis[1-(2,2,6,6-tetramethyl-4-piperidylidene)semicarbazide] | 15.03 | 588.3904 | 588 | C33H48N8O2 | 58/98/42/41/153 |
|  | 4-Azepan-1-yl-oxazolidin-2-one | 10.53 | 184.1212 | 184 | C9H16N2O2 | 70/42/57/41/15 |
|  | 4-Methyl-2-oxopentanenitrile | 13.16 | 111.0684 | 111 | C6H9NO | 41/70/57/85/27 |
|  | 5,5-Dimethyl-3-(3-methyl-oxiran-2-yl)-cyclohex-2-enone | 13.79 | 180.115 | 180 | C11H16O2 | 96/83/52/67/152 |
|  | 5,7-Dimethyloctahydrocoumarin | 25.20 | 182.1307 | 182 | C11H18O2 | 95/55/41/110/81 |
|  | 5-Ethoxy-cyclooctene | 23.67 | 154.1358 | 154 | C10H18O | 67/41/80/99/43 |
|  | 5H-1,2,4-Triazolo[4,3-b][1,2,4]triazepine, 6,7,8,9-tetrahydro-6-methyl- | 15.84 | 153.1016 | 153 | C6H11N5 | 55/153/70/57/41 |
|  | 6,10,13-Trimethyltetradecanol | 25.21 | 256.2767 | 256 | C17H36O | 55/97/41/71/111 |
|  | 9-Hexacosene | 12.82 | 364.4071 | 364 | C26H52 | 43/97/57/83/41 |
|  | Acetic acid, 2-propyltetrahydropyran-3-yl ester | 14.71 | 186.1256 | 186 | C10H18O3 | 43/71/98/41/57 |
|  | Aziridine, 2-(1,1-dimethylethyl)-3-methyl-1-(2-propenyl)-, trans- | 10.53 | 153.1518 | 153 | C10H19N | 41/44/112/69/27 |
|  | Boron, (2-aminoethanolato-N,O)(1,5-cyclooctanediyl)-, Tt-4)- | 15.87 | 180.9655 | 181 | C10H20BNO | 44/85/124/181/98 |
|  | cis-5-Methyl-2-isopropyl-2-hexen-1-al | 9.09 | 154.1358 | 154 | C10H18O | 41/69/111/97/29 |
|  | Cyclohexanone, 2-methyl-5-(1-methylethyl)- | 25.20 | 154.1358 | 154 | C10H18O | 55/111/41/69/83 |
|  | Cyclopentanecarboxylic acid, 2-tetradecyl ester | 14.93 | 310.2873 | 310 | C20H38O2 | 69/97/41/43/115 |
|  | Cyclopropane, 1-(2-chloro-1-hexen-1-ylideno)-2,2,3,3-tetramethyl- | 14.49 | 212.1333 | 212 | C13H21Cl | 41/141/105/91/119 |
|  | Decane, 1-bromo-2-methyl- | 16.28 | 234.0983 | 234 | C11H23Br | 57/43/71/41/85 |
|  | Heneicosane, 5-methyl- | 15.08 | 310.3601 | 310 | C22H46 | 43/57/85/41/71 |
|  | Heptane, 2-(hexyloxy)- | 10.72 | 200.2141 | 200 | C13H28O | 43/85/57/41/129 |
|  | Heptane, 3-(bromomethyl)- | 14.73 | 208.0463 | 192 | C8H17Br | 57/55/41/71/27 |
|  | Methyl-2-methoxyoct-2-enoate | 10.70 | 186.1256 | 186 | C10H18O3 | 43/41/115/85/69 |
|  | Muscimol | 12.81 | 114.0429 | 114 | C4H6N2O2 | 44/83/69/111/97 |
|  | N-Ethoxyisobuten-3-imine | 12.63 | 113.0841 | 113 | C6H11NO | 55/83/41/58/98 |
|  | Nonane, 4-ethyl-5-methyl- | 10.72 | 170.2035 | 170 | C12H26 | 43/41/84 |
|  | n-Propyl heptyl ether | 13.04 | 158.1671 | 158 | C10H22O | 43/57/70/41/97 |
|  | Octadecanoic acid, 2,3-bis[(1-oxotetradecyl)oxy]propyl ester | 15.46 | 778.7054 | 778 | C49H94O6 | 55/41/71/98/85 |
|  | Octanal | 16.08 | 128.1201 | 128 | C8H16O | 43/41/56/84/27 |
|  | Octane, 1,2-dibromo- | 14.94 | 269.9618 | 270 | C8H16Br2 | 69/41/55/29/111 |
|  | Spiro[2.4]heptane-5-methanol, 5-hydroxy- | 15.47 | 142.0994 | 142 | C8H14O2 | 93/111/55/41/67 |
|  | Spiro[3,5-dioxatricyclo[6.3.0.0(2,7)]undecan-6-one-4,2'cyclohexane], 1'-isopropyl-2,4'-dimethyl-, E- | 14.62 | 306.2195 | 306 | C19H30O3 | 155/41/124/81/43 |
|  | trans-2,3-Epoxyoctane | 10.64 | 128.1201 | 128 | C8H16O | 56/27/45/82/99 |
|  | Vinyl lauryl ether | 14.76 | 212.2141 | 212 | C14H28O | 43/57/41/71/85 |
| KSC | (E)-Dodec-2-enyl isobutyl carbonate | 13.94 | 284.2352 | 284 | C17H32O3 | 57/96/82/41/55 |
|  | 1(2H)-Naphthalenone, octahydro-8a-methyl-, cis- | 16.58 | 166.1358 | 166 | C11H18O | 111/95/41/81/124 |
|  | 1-(6-Aza-uracil-1-yl)-3-(5-methyl-uracil-1-yl)-propane | 15.35 | 279.0968 | 279 | C11H13N5O4 | 55/153/40/110/126 |
|  | 1,2-Cyclohexanediol, 3-methyl-6-(1-methylethyl)-, (1.alpha.,2.beta.,3.beta.,6.alpha.)- | 25.18 | 172.1464 | 172 | C10H20O2 | 43/41/111/84/139 |
|  | 1,3,2-Dioxaborinane, 2,4-diethyl-5-methyl-6-propyl- | 13.44 | 197.9808 | 198 | C11H23BO2 | 155/70/57/41/55 |
|  | 1,3-Cyclohexanedione, 2-methyl-2-(3-oxobutyl)- | 14.70 | 196.1099 | 196 | C11H16O3 | 43/111/196/97/41 |
|  | 1,5-Diacetyl-3,7-bis(3-chloropropionyl)-octahydro-1,3,5,7-tetraazocine | 15.48 | 380.102 | 380 | C14H22Cl2N4O4 | 43/85/63/36/113 |
|  | 10-Oxocyclodec-2-enecarboxylic acid, methyl ester | 18.57 | 210.1256 | 210 | C12H18O3 | 44/98/41/67/81 |
|  | 10-Undecenoic acid, 2-hydroxy-, methyl ester | 15.39 | 214.1569 | 214 | C12H22O3 | 95/41/55/81/69 |
|  | 11-Dodecen-1-ol, 2,4,6-trimethyl-, (R,R,R)- | 16.17 | 226.2298 | 226 | C15H30O | 55/69/83/41/97 |
|  | 17-Pentatriacontene | 16.78 | 490.5481 | 490 | C35H70 | 57/43/97/83/41 |
|  | 1-Allyl-4-dimethylamino-6-methoxy-1,3,5-triazin-2(1H)-one | 18.55 | 210.1118 | 210 | C9H14N4O2 | 184/44/83/210/56 |
|  | 1-Bromo-11-iodoundecane | 14.40 | 359.995 | 360 | C11H22BrI | 69/41/97/71/111 |
|  | 1-Bromo-4-bromomethyldecane | 15.38 | 312.0088 | 312 | C11H22Br2 | 41/57/71/97/163 |
|  | 1-Hentetracontanol | 12.63 | 592.6526 | 592 | C41H84O | 57/43/83/97/111 |
|  | 1H-Imidazole, 1-[(3-ethoxy-4-fluorophenyl)sulfonyl]-4,5-dihydro-2-methyl- | 16.67 | 286.0788 | 286 | C12H15FN2O3S | 55/83/56/203/139 |
|  | 1-Oxaspiro[2.5]octan-4-one, 2,2-dimethyl- | 17.22 | 154.0994 | 154 | C9H14O2 | 139/41/67/111/86 |
|  | 2,2,6,8,12-Pentamethyl-7,9,10-trioxa-tricyclo[6.2.2.0(1,6)]dodec-11-ene | 15.41 | 238.1569 | 238 | C14H22O3 | 43/191/69/109/41 |
|  | 2,2-Dimethyl-10-imino-3-oxa-5,8,11,14-tetraazatetradecane-4,7,13-trione | 14.50 | 287.1595 | 287 | C11H21N5O4 | 29/57/140/214/43 |
|  | 2,3-Dioxabicyclo[2.2.2]octane | 16.60 | 114.0681 | 114 | C6H10O2 | 29/27/81/57/114 |
|  | 2,4,6(1H,3H,5H)-Pyrimidinetrione, 5-propionyl- | 16.20 | 184.0484 | 184 | C7H8N2O4 | 155/184/69/44/112 |
|  | 2-Aziridinone, 1-tert-butyl-3-(1-methylcyclopentyl)- | 14.36 | 195.1624 | 195 | C12H21NO | 41/57/83/96/195 |
|  | 2-Hexyldodecyl isobutyrate | 14.83 | 340.3343 | 340 | C22H44O2 | 43/71/57/111/41 |
|  | 2H-Quinolizin-2-ol, octahydro- | 14.11 | 155.1311 | 155 | C9H17NO | 97/154/138/83/55 |
|  | 2-Isopropyl-5-methylhex-2-enal | 15.45 | 154.1358 | 154 | C10H18O | 41/69/27/97/111 |
|  | 2-Pentenal, 2,4,4-trimethyl- | 10.33 | 126.1045 | 126 | C8H14O | 41/55/111/27/97 |
|  | 2-Trifluoroacetoxydodecane | 13.70 | 282.1807 | 282 | C14H25F3O2 | 41/57/70/84/98 |
|  | 3-Pyrrolidinecarboxylic acid, 2,4-dioxo-, methyl ester | 10.37 | 157.0375 | 157 | C6H7NO4 | 31/44/99/69/157 |
|  | 4-Piperidinamine, N,1-dimethyl- | 13.80 | 128.1314 | 128 | C7H16N2 | 97/43/70/98/56 |
|  | 4-Undecanone, 7-ethyl-2-methyl- | 15.54 | 212.2141 | 212 | C14H28O | 69/41/83/27/155 |
|  | 5H-Cyclohepta-1,4-dioxin, 2,3,4a,6,7,9a-hexahydro-,cis- | 14.88 | 154.0994 | 154 | C9H14O2 | 27/41/73/55/67 |
|  | 6-Acetyl-.beta.-d-mannose | 18.54 | 222.0739 | 222 | C8H14O7 | 43/41/97/126/60 |
|  | 6-Bromohexanoic acid, 4-methoxy-2-methylbutyl ester | 15.36 | 294.083 | 294 | C12H23BrO3 | 58/45/85/70/177 |
|  | 9,10-Secochola-5,7,10(19)-trien-24-al, 3-hydroxy-, (3.beta.,5Z,7E)- | 16.04 | 356.2716 | 356 | C24H36O2 | 118/136/55/91/81 |
|  | 9-Methyl-Z-10-pentadecen-1-ol | 17.10 | 240.2454 | 240 | C16H32O | 43/57/41/99/83 |
|  | 9-Oxabicyclo[3.3.1]nonane, 3-acetoxy-2-(tetrahydropyran-2-yloxy)- | 23.67 | 284.1624 | 284 | C15H24O5 | 85/43/41/57/88 |
|  | Adenosine, 4'-de(hydroxymethyl)-4'-[N-ethylaminoformyl]- | 18.57 | 442.1602 | 442 | C20H22N6O6 | 43/60/41/95/124 |
|  | Benzenepropanoic acid, 6-ethyl-3-octyl ester | 15.86 | 290.2247 | 290 | C19H30O2 | 150/91/105/133/71 |
|  | Butan-1-one, 1-[4-(2-fluoro-5-methyl-4-nitrophenyl)-1-piperazinyl]- | 14.89 | 309.149 | 309 | C15H20FN3O3 | 43/56/209/41/71 |
|  | Butanoic acid, 2,7-dimethyloct-7-en-5-yn-4-yl ester | 16.58 | 222.162 | 222 | C14H22O2 | 179/71/43/109/91 |
|  | Butanoic acid, 2-[(trifluoroacetyl)amino]-, 1-methylpentyl ester | 13.78 | 283.1395 | 283 | C12H20F3NO3 | 154/41/57/140/126 |
|  | Card-20(22)-enolide, 2,3,14-trihydroxy-, (2.alpha.,3.beta.,5.alpha.)- | 14.93 | 390.2407 | 390 | C23H34O5 | 85/111/201/55/41 |
|  | Cholest-8-ene-3,6-diol, 14-methyl-, (3.beta.,5.alpha.,6.alpha.)- | 15.91 | 416.3656 | 416 | C28H48O2 | 44/41/95/57/383 |
|  | Cyclobutane, 1-butyl-2-ethyl- | 10.43 | 140.1566 | 140 | C10H20 | 69/55/41/27/84 |
|  | Cycloheptanone, 2-(2-methylpropylidene)- | 15.85 | 166.1358 | 166 | C11H18O | 67/41/95/166/81 |
|  | Cyclohexane, 1,1'-(1-methyl-1,3-propanediyl)bis- | 13.18 | 222.2349 | 222 | C16H30 | 83/55/41/69/111 |
|  | Cyclohexane, 1,1'-[methylenebis(oxy)]bis- | 15.04 | 212.1777 | 212 | C13H24O2 | 55/57/41/83/100 |
|  | Cyclohexane, 1,1'-tetradecylidenebis- | 14.67 | 362.3915 | 362 | C26H50 | 55/83/41/97/278 |
|  | Cyclohexanol, 2,2,6,6-tetramethyl- | 25.22 | 156.1515 | 156 | C10H20O | 109/82/69/41/43 |
|  | Cyclohexanone, dimethylhydrazone | 13.32 | 140.1314 | 140 | C8H16N2 | 44/140/41/69/96 |
|  | Cyclopentadecane | 14.30 | 210.2349 | 210 | C15H30 | 83/55/69/97/210 |
|  | Dichloroacetic acid, 3-tridecyl ester | 13.15 | 310.1468 | 310 | C15H28Cl2O2 | 57/41/83/97/111 |
|  | Dichloroacetic acid, undecyl ester | 9.11 | 282.1155 | 282 | C13H24Cl2O2 | 69/97/55/41/111 |
|  | Dimethylmalonic acid, monochloride, 2-octyl ester | 14.93 | 286.1336 | 262 | C13H23ClO3 | 133/57/71/41/43 |
|  | d-Mannitol, 1,1'-O-1,16-hexadecanediylbis- | 16.43 | 586.3929 | 586 | C28H58O12 | 69/55/129/97/111 |
|  | Docosane, 7-hexyl- | 15.73 | 394.4541 | 394 | C28H58 | 57/43/71/85/41 |
|  | Glycine, N-methyl-N-(trifluoroacetyl)-, 1-methylpropyl ester | 16.22 | 241.0926 | 241 | C9H14F3NO3 | 140/57/41/42/168 |
|  | Glycyl-D-asparagine | 10.36 | 189.075 | 189 | C6H11N3O4 | 44/18/28/56/100 |
|  | Heneicosane, 11-pentyl- | 14.40 | 366.4228 | 366 | C26H54 | 43/57/71/41/85 |
|  | Heptanal | 9.08 | 114.1045 | 114 | C7H14O | 70/41/57/27/86 |
|  | Heptane, 2,3-epoxy- | 15.09 | 114.1045 | 114 | C7H14O | 45/85/41/57/27 |
|  | Hexadecane, 1-(ethenyloxy)- | 17.10 | 268.2767 | 268 | C18H36O | 57/43/41/83/97 |
|  | Hexanoic acid, 2-propenyl ester | 11.60 | 156.115 | 156 | C9H16O2 | 99/41/71/69/27 |
|  | Hexatriacontyl pentafluoropropionate | 15.36 | 668.5533 | 668 | C39H73F5O2 | 57/71/43/97/111 |
|  | Homogentisic acid, dimethyl ether, methyl ester | 14.74 | 210.0892 | 210 | C11H14O4 | 210/151/121/163/91 |
|  | Isophytol, acetate | 15.46 | 338.3186 | 338 | C22H42O2 | 43/71/57/113/95 |
|  | Isopinocarveol | 16.15 | 152.1201 | 152 | C10H16O | 41/55/70/92/69 |
|  | Milbemycin b, 13-chloro-5-demethoxy-28-deoxy-6,28-epoxy-5-(hydroxyimino)-25-(1-methylethyl)-, (6R,13R,25R)- | 15.86 | 603.2964 | 603 | C33H46ClNO7 | 55/181/95/69/41 |
|  | N-(2-Methylbutyl)(2E,4E,8Z,10E)-dodecatetra | 15.81 | 261.2094 | 261 | C17H27NO | 81/181/41/57/95 |
|  | N-[3-[N-Aziridyl]propyl]cyclohexylamine | 16.10 | 182.1784 | 182 | C11H22N2 | 56/41/70/125/96 |
|  | N-carbobenzyloxy-.gamma.-aminobutyryl-dl-alanine | 16.32 | 308.1373 | 308 | C15H20N2O5 | 91/79/108/44/65 |
|  | Nonyl chloroformate | 9.10 | 206.1074 | 206 | C10H19ClO2 | 56/43/70/41/84 |
|  | Octahydro-2H-quinolizine | 16.00 | 139.1362 | 139 | C9H17N | 138/97/83/110/55 |
|  | Octane, 1-bromo- | 18.50 | 192.0514 | 192 | C8H17Br | 57/41/135/71/27 |
|  | Octane, 2-cyclohexyl- | 14.21 | 196.2192 | 196 | C14H28 | 82/41/55/29/69 |
|  | Oxalic acid, allyl octadecyl ester | 13.84 | 382.3084 | 382 | C23H42O4 | 41/43/57/71/97 |
|  | Phosphonic acid, (3-methylene-2-oxopentyl)-,diethyl ester | 14.88 | 234.1021 | 234 | C10H19O4P | 155/99/81/29/127 |
|  | Phosphonic acid, pentyl-, diethyl ester | 16.24 | 208.1229 | 208 | C9H21O3P | 152/125/29/111/138 |
|  | Piperazine, 1-hex-2-ynyl-4-methyl- | 15.46 | 180.1628 | 180 | C11H20N2 | 42/56/41/97/180 |
|  | Tricosyl trifluoroacetate | 25.20 | 436.353 | 436 | C25H47F3O2 | 57/43/97/83/41 |
|  | Tricyclo[3.3.1.1(3,7)]decane-2,6-dione, 4-hydroxy- | 15.34 | 180.0786 | 180 | C10H12O3 | 180/55/95/107/79 |
|  | Z-7-Pentadecenol | 14.11 | 226.2298 | 226 | C15H30O | 97/55/69/83/98 |
|  | Bicyclo[4.1.0]heptan-2-ol, 3,7,7-trimethyl-, (1.alpha.,2.alpha.,3.beta.,6.alpha.)- | 13.74 | 154.1358 | 154 | C10H18O | 41/111/82/67/97 |
| KSP | 1,7-Octadiene, 3-methoxy- | 10.52 | 140.1201 | 140 | C9H16O | 71/41/27/67/54 |
|  | 1H-Imidazole-4,5-dicarboxylic acid, 1-(2-oxopropyl)- | 15.38 | 212.0433 | 212 | C8H8N2O5 | 152/43/170/108/124 |
|  | 1-Pentacontanol | 14.83 | 718.7935 | 718 | C50H102O | 57/43/71/97/41 |
|  | 1-Trifluorosilyltridecane | 14.27 | 268.2926 | 268 | C13H27F3Si | 43/57/71/41/85 |
|  | 2-Methyl-Z-4-tetradecene | 15.25 | 210.2349 | 210 | C15H30 | 43/57/41/83/97 |
|  | 3-Octenoic acid, butyl ester, (Z)- | 14.67 | 198.162 | 198 | C12H22O2 | 41/55/81/95/123 |
|  | 4-Quinolinamine, decahydro-1-methyl- | 15.82 | 168.1628 | 168 | C10H20N2 | 44/150/111/41/125 |
|  | 6-Octadecenoic acid | 14.67 | 282.256 | 282 | C18H34O2 | 55/97/83/69/264 |
|  | 9-Octadecenoic acid (Z)-, hexyl ester | 10.44 | 366.3499 | 366 | C24H46O2 | 43/41/56/27/88 |
|  | Cyclohexanecarboxylic acid, 3,5-dimethylcyclohexyl ester | 12.82 | 238.1933 | 238 | C15H26O2 | 110/69/55/95/83 |
|  | Cyclohexanecarboxylic acid, 4-(1,5-dimethyl-3-oxohexyl)-, methyl ester, cis- | 13.71 | 268.2039 | 268 | C16H28O3 | 57/108/85/41/81 |
|  | Cyclotridecane | 11.43 | 182.2035 | 182 | C13H26 | 41/55/69/83/97 |
|  | Decane, 2,9-dimethyl- | 15.68 | 170.2035 | 170 | C12H26 | 43/57/71/41/85 |
|  | Dichloroacetic acid, tridecyl ester | 13.01 | 310.1468 | 310 | C15H28Cl2O2 | 43/57/83/41/97 |
|  | E-8-Methyl-7-dodecen-1-ol acetate | 15.76 | 240.209 | 240 | C15H28O2 | 43/41/97/69/81 |
|  | Heptadecane, 2,6,10,15-tetramethyl- | 14.02 | 296.3445 | 296 | C21H44 | 57/71/43/85/41 |
|  | Isoxazole, 5-amino-3-butyl-4-propyl- | 14.65 | 182.142 | 182 | C10H18N2O | 125/153/82/56/42 |
|  | Nonadecane, 9-methyl- | 11.49 | 282.3288 | 282 | C20H42 | 57/71/43/85/41 |
|  | Oxalic acid, isohexyl undecylester | 23.66 | 328.2614 | 328 | C19H36O4 | 43/85/57/41/71 |
|  | Pyrimidine, 5-ethoxy-4-methoxy-2-methyl- | 15.02 | 168 .0899 | 168 | C8H12N2O2 | 168/139/111/82/55 |
|  | Streptovitacin A | 23.69 | 297.1576 | 297 | C15H23NO5 | 43/41/113/84/69 |
|  | Thiophene-2-carboxaldehyde, 3-methoxy- | 15.03 | 142.0088 | 142 | C6H6O2S | 142/45/124/96/111 |
|  | Triacontyl trifluoroacetate | 14.84 | 534.4626 | 534 | C32H61F3O2 | 57/43/97/71/111 |
|  | Tridecanol, 2-ethyl-2-methyl- | 12.85 | 242.2611 | 242 | C16H34O | 57/71/43/85/41 |
|  | Trifluoroacetic acid,n-tridecyl ester | 18.90 | 296.1964 | 296 | C15H27F3O2 | 69/55/83/97/41 |
|  | Undecane, 4,8-dimethyl- | 12.85 | 184.2192 | 184 | C13H28 | 43/71/57/41/85 |
| NRC | (1R,2R,3S,5R)-(-)-2,3-Pinanediol | 14.50 | 170.1307 | 170 | C10H18O2 | 43/83/69/99/41 |
|  | Ethyl 2-acetamido-2-(sec-butylamino)-3,3,3-trifluoropropionate | 14.65 | 284.1348 | 284 | C11H19F3N2O3 | 113/44/169/211/57 |
|  | Tridecane, 6-propyl- | 14.94 | 226.2662 | 226 | C16H34 | 43/57/71/41/85 |
|  | 1-(.beta.-d-Ribofuranosyl)-4-difluormethoxy-uracil | 16.22 | 294.0663 | 294 | C10H12F2N2O6 | 133/31/69/43/96 |
|  | 1,2,5-Triazole, 1-octyl-3-nitro-4-formamido-, 2-oxide | 13.15 | 285.1438 | 285 | C11H19N5O4 | 43/71/57/41/99 |
|  | 1,3,2-Dioxaborolane, 2-ethyl-4-(3-oxiranylpropyl)- | 11.55 | 183.9288 | 184 | C9H17BO3 | 99/43/27/57/70 |
|  | 1,3,5-Triazine-2,4-diamine, N-ethyl-6-methoxy- | 13.71 | 169.0965 | 169 | C6H11N5O | 169/43/141/154/69 |
|  | 1,3-Cyclohexanedione, 2-butyl-2-methyl- | 15.40 | 182.1307 | 182 | C11H18O2 | 111/41/69/55/39 |
|  | 1-Dodecanol, 3,7,11-trimethyl- | 15.16 | 228.2454 | 228 | C15H32O | 55/69/41/71/97 |
|  | 1-Methyl-4-nitro-5-[(3-chloropropyl)amino]-(1H)-imidazole | 15.03 | 218.0572 | 218 | C7H11ClN4O2 | 155/57/137/96/218 |
|  | 1-Octene, 2,6-dimethyl- | 9.12 | 140.1566 | 140 | C10H20 | 56/55/70/41/84 |
|  | 1-Pentacosanol | 25.20 | 368.402 | 368 | C25H52O | 97/83/57/41/111 |
|  | 2,3-Dimethyldodecane | 12.89 | 198.2349 | 198 | C14H30 | 43/57/71/41/85 |
|  | 2,5-Octadecadienoic acid, methyl ester | 16.80 | 294.256 | 294 | C19H34O2 | 41/79/67/111/139 |
|  | 2H-1,2-Oxaborin, 3,6-dihydro-2,3,3-tripropyl- | 17.68 | 208.0016 | 208 | C13H25BO | 95/138/44/41/81 |
|  | 2-Octyldecyl acetate | 15.46 | 312.303 | 312 | C20H40O2 | 43/57/83/97/41 |
|  | 2-Thiopheneacetic acid, 3-tetradecyl ester | 16.20 | 338.2281 | 338 | C20H34O2S | 57/97/43/71/41 |
|  | 2-Trifluoroacetoxytridecane | 14.08 | 296.1964 | 296 | C15H27F3O2 | 41/69/70/97/111 |
|  | 3-(E)-Hepten-2-one, (5S)-5-[(t-butoxycarbonyl)amino]-6-methyl- | 11.56 | 241.1678 | 241 | C13H23NO3 | 57/98/142/41/124 |
|  | 3,5-Dinitro-benzoic acid, 2-hydroxy-4-[2-(4-hydroxybutyl)[1,3]dithian-2-yl]butyl ester | 16.27 | 474.1131 | 474 | C19H26N2O8S2 | 71/41/141/55/212 |
|  | 3,7-Dimethyl-6-nonen-1-ol acetate | 14.93 | 212.1777 | 212 | C13H24O2 | 43/123/81/41/95 |
|  | 3-Ethyl-3-methylheptane | 15.09 | 142.1722 | 142 | C10H22 | 57/43/71/85/113 |
|  | 3-Phenylpropionic acid, oct-3-en-2-yl ester | 15.85 | 260.1777 | 260 | C17H24O2 | 69/91/105/55/133 |
|  | 3-Tetradecene, (E)- | 9.08 | 196.2192 | 196 | C14H28 | 41/55/69/70/97 |
|  | 3-Trifluoroacetoxytridecane | 10.53 | 296.1964 | 296 | C15H27F3O2 | 41/43/69/70/97 |
|  | 4-Dimethylamino-4-cyano-1,2,5-trimethylpiperidine | 20.35 | 195.1737 | 195 | C11H21N3 | 70/42/135/153/57 |
|  | 4H-1,3,4-Triazol-3-amine, N-dimethylaminome | 14.93 | 139.0859 | 139 | C5H9N5 | 44/139/83/57/40 |
|  | 4-Pentylcyclohexanone | 15.37 | 168.1515 | 168 | C11H20O | 97/41/55/83/126 |
|  | 4-Trifluoroacetoxypentadecane | 12.82 | 324.2277 | 324 | C17H31F3O2 | 55/41/69/83/97 |
|  | 6-Hexadecanone | 14.66 | 240.2454 | 240 | C16H32O | 43/71/58/41/99 |
|  | 3-Tetradecene, (E)- | 11.42 | 196.2192 | 196 | C14H28 | 41/55/69/70/97 |
|  | 8,9,9,10,10,11-Hexafluoro-4,4-dimethyl-3,5-dioxatetracyclo[5.4.1.0(2,6).0(8,11)]dodecane | 18.14 | 302.0741 | 302 | C12H12F6O2 | 43/40/57/81/287 |
|  | 9-Oxa-bicyclo[3.3.1]nonane-2,7-diol | 14.91 | 158.0943 | 158 | C8H14O3 | 97/45/41/73/101 |
|  | 9-Oxabicyclo[4.2.1]nonan-2-ol | 25.26 | 142.0994 | 142 | C8H14O2 | 81/57/41/43/97 |
|  | Acetamide, N-cyclopentyl-2-(2-methyl-5-nitroimidazol-1-yl)- | 15.84 | 252.1223 | 252 | C11H16N4O3 | 43/41/69/141/185 |
|  | Acetic acid, cyano-, 2-ethylhexyl ester | 13.84 | 197.1416 | 197 | C11H19NO2 | 57/41/55/70/27 |
|  | Bromoacetic acid, hexadecyl ester | 15.25 | 362.1821 | 362 | C18H35BrO2 | 41/69/83/97/111 |
|  | Butanamide, 3-cyclohexylamino-4-hydroxy-N- | 10.36 | 282.2309 | 282 | C16H30N2O2 | 41/55/56/83/98 |
|  | Butanamide, N-(5-methyl-3-isoxazolyl)-2-[[4-methyl-5-(2-methylpropyl)-1H-imidazol-2-yl]thio]- | 15.04 | 336.1622 | 336 | C16H24N4O2S | 41/127/179/68/184 |
|  | Cyclododecanol, 1-aminomethyl- | 15.90 | 213.2094 | 213 | C13H27NO | 30/55/183/83/67 |
|  | Cycloheptanone, 2-ethyl- | 15.53 | 140.1201 | 140 | C9H16O | 55/41/112/98/56 |
|  | Cyclohexane, 1,1'-(2-propyl-1,3-propanediyl)bis- | 15.79 | 250.2662 | 250 | C18H34 | 55/83/41/97/69 |
|  | Cyclopentane, (3-methylbutyl)- | 10.34 | 140.1566 | 140 | C10H20 | 69/41/55/70/84 |
|  | Cyclopropanecarboxylic acid, nonyl ester | 15.90 | 212.1777 | 212 | C13H24O2 | 69/87/41/70/126 |
|  | Cycloundecane, (1-methylethyl)- | 14.71 | 196.2192 | 196 | C14H28 | 55/41/83/84/27 |
|  | Decanoic acid, 2-propenyl ester | 15.09 | 212.1777 | 212 | C13H24O2 | 41/57/100/155/71 |
|  | Dodeca-1,6-dien-12-ol, 6,10-dimethyl- | 16.15 | 210.1984 | 210 | C14H26O | 81/55/41/67/95 |
|  | Dodecane, 3-cyclohexyl- | 15.32 | 252.2818 | 252 | C18H36 | 55/83/41/97/125 |
|  | Ethanal, 2-(3-ethyl-2,2-dimethylcyclobutyl)-, semicarbazone | 13.65 | 211.1686 | 211 | C11H21N3O | 44/69/41/128/86 |
|  | Geldaramycin | 23.73 | 560.2735 | 560 | C29H40N2O9 | 43/57/138/95/41 |
|  | Heptadecyl trifluoroacetate | 12.72 | 352.259 | 352 | C19H35F3O2 | 57/43/97/83/41 |
|  | Heptane, 3-[(ethenyloxy)methyl]- | 14.77 | 156.1515 | 156 | C10H20O | 57/43/71/41/27 |
|  | Isobutyl nonyl carbonate | 13.04 | 244.2039 | 244 | C14H28O3 | 57/71/41/85/98 |
|  | Isodecyl methacrylate | 12.82 | 226.1933 | 226 | C14H26O2 | 69/70/41/55/111 |
|  | Octane, 2,3,3-trimethyl- | 15.68 | 156.1879 | 156 | C11H24 | 43/57/71/41/85 |
|  | Oxalic acid, allyl tetradecyl ester | 10.34 | 326.2458 | 326 | C19H34O4 | 41/43/57/71/85 |
|  | Oxalic acid, bis(6-ethyloct-3-yl) ester | 15.37 | 370.3084 | 370 | C22H42O4 | 57/85/43/71/41 |
|  | Pentadecane, 3-methyl- | 13.28 | 226.2662 | 226 | C16H34 | 57/41/71/85/197 |
|  | Pentafluoropropionic acid, hexadecyl ester | 14.84 | 388.2401 | 388 | C19H33F5O2 | 55/97/83/41/111 |
|  | Piperazine, 2-methyl- | 16.52 | 100.1001 | 100 | C5H12N2 | 44/85/57/30/100 |
|  | Piperidine N-ethyl-4-[1-aminoethyl]- | 23.60 | 156.1628 | 156 | C9H20N2 | 44/58/84/98/124 |
|  | Piperidine, 3-isopropyl- | 23.37 | 127.1362 | 127 | C8H17N | 44/84/127/70/58 |
|  | Spiro[2.3]hexan-4-one, 5,5-diethyl- | 14.67 | 152.1201 | 152 | C10H16O | 41/27/55/67/123 |
|  | Sulfurous acid, decyl hexyl ester | 15.09 | 306.223 | 306 | C16H34O3S | 85/43/57/71/41 |
|  | Tetradecane, 1-chloro- | 14.95 | 232.1959 | 232 | C14H29Cl | 57/43/91/71/41 |
|  | Tricyclo[5.2.1.0(2,6)]decane, 2-acetoxy- | 14.92 | 194.1307 | 194 | C12H18O2 | 84/43/67/106/134 |
|  | Tridecane, 4-methyl- | 19.01 | 198.2349 | 198 | C14H30 | 43/57/71/85/41 |
|  | Undecane, 5-cyclohexyl- | 14.66 | 238.2662 | 238 | C17H34 | 55/83/41/97/111 |
|  | Z-12-Tetradecenal | 18.59 | 210.1984 | 210 | C14H26O | 55/69/98/41/81 |
| NqSC | (2S,3S)-(-)-3-Propyloxiranemethanol | 18.99 | 116.0837 | 116 | C6H12O2 | 55/29/27/57/73 |
|  | .beta.-Alanine, N-methyl-N-(1-methyl-4-nitro-1H-imidazol-5-yl)-, methyl ester | 15.85 | 242.1016 | 242 | C9H14N4O4 | 141/165/82/123/169 |
|  | 1,3-Cyclohexanedione, 5-isopropyl- | 14.94 | 154.0994 | 154 | C9H14O2 | 97/41/69/55/83 |
|  | 1,5,9-Cyclododecanetriol | 14.51 | 216.1726 | 216 | C12H24O3 | 55/41/81/27/95 |
|  | 1,5-Dinitro-3,7-diazabicyclo[3.3.1]nonane | 13.74 | 216.0859 | 216 | C7H12N4O4 | 69/41/216/71/96 |
|  | 1-.beta.-d-3'-Anhydro-uridine | 14.94 | 226.0589 | 226 | C9H10N2O5 | 113/31/96/69/226 |
|  | 1-Hexadecanesulfonyl chloride | 12.72 | 324.1891 | 324 | C16H33ClO2S | 57/43/83/97/41 |
|  | 1H-Pyrazole, 1,5-dimethyl-4-nitro- | 15.06 | 141.0539 | 141 | C5H7N3O2 | 56/42/124/141/111 |
|  | 1-Octyl trifluoroacetate | 9.08 | 226.1181 | 226 | C10H17F3O2 | 69/55/41/70/84 |
|  | 2-(Dimethylamino)-3-methyl-1-butene | 9.17 | 113.1205 | 113 | C7H15N | 43/98/113/56/41 |
|  | 2,3-Dimethylcyclohexylamine | 9.10 | 127.1362 | 127 | C8H17N | 56/84/110/43/127 |
|  | 2-Acetamido-2-deoxymannosonic acid | 15.38 | 235.0691 | 235 | C8H13NO7 | 43/99/141/123/72 |
|  | 2-Hexadecene, 2,6,10,14-tetramethyl- | 13.15 | 280.3132 | 280 | C20H40 | 43/57/41/70/97 |
|  | 2-Hexanone, 5-methyl-3-methylene- | 15.79 | 126.1045 | 126 | C8H14O | 43/69/41/27/111 |
|  | 2H-Oxecin-2-one, 3,4,7,8,9,10-hexahydro-4-hydroxy-10-methyl-, [4S-(4R*,5E,10S*)]- | 14.52 | 184.1099 | 184 | C10H16O3 | 70/55/41/69/95 |
|  | 3,7-Diazabicyclo[3.3.1]nonane, 9,9-dimethyl- | 14.50 | 154.1471 | 154 | C9H18N2 | 41/69/154/70/96 |
|  | 3,7-Dimethyloct-6-enyl ethyl carbonate | 16.16 | 228.1726 | 228 | C13H24O3 | 95/123/81/69/41 |
|  | 3-Cyclohexen-1-ol, 4-methyl-1-(1-methylethyl)-, (R)- | 15.89 | 154.1358 | 154 | C10H18O | 71/111/43/93/69 |
|  | 4-[(2-Oxoazepan-3-yl)amino]pent-3-en-2-one | 14.30 | 210.1369 | 210 | C11H18N2O2 | 43/84/28/152/110 |
|  | 4-[3-(4-Methylbenzyloxy)propyl]-1H-imidazole | 15.87 | 230.142 | 230 | C14H18N2O | 105/82/125/95/54 |
|  | 4-Cyclohexylidene-n-butanol | 25.24 | 154.1358 | 154 | C10H18O | 55/70/69/111/85 |
|  | 5-Nonanone, 2,2,8,8-tetramethyl- | 13.29 | 198.1984 | 198 | C13H26O | 57/113/43/41/85 |
|  | 7,7-Dimethyl-2-oxo-1,8-dioxaspiro[4.5]decane-4-carboxamide | 16.24 | 227.1158 | 227 | C11H17NO4 | 43/125/212/41/99 |
|  | 9-methylheptadecane | 10.72 | 254.2975 | 254 | C18H38 | 57/71/43/85/41 |
|  | 9-Octadecene, 1,1'-[1,2-ethanediylbis(oxy)]bis- | 14.68 | 562.5692 | 562 | C38H74O2 | 55/69/83/97/41 |
|  | Bicyclo[2.2.1]heptan-2-amine, N,N,1,7,7-pentamethyl- | 14.70 | 181.1831 | 181 | C12H23N | 95/110/41/72/42 |
|  | Chloroacetic acid, 4-hexadecyl ester | 14.37 | 318.2327 | 318 | C18H35ClO2 | 55/57/97/83/41 |
|  | Cyano(5'-ethoxy-1,2,3,4,3',4'-hexahydro-2'H-[2,2']bipyrrolyl-5-ylidene)acetic acid, t-butyl ester | 16.01 | 319.1897 | 319 | C17H25N3O3 | 84/41/57/55/112 |
|  | Cyclohexanone, 2,6-diethyl- | 15.04 | 154.1358 | 154 | C10H18O | 55/41/83/126/56 |
|  | Cyclopropane, 1-(1,2-dimethylpropyl)-1-methyl-2-nonyl- | 12.82 | 252.2818 | 252 | C18H36 | 69/83/43/41/97 |
|  | Cyclopropanemethanol, 2,2,3,3-tetramethyl- | 15.46 | 128.1201 | 128 | C8H16O | 55/97/41/67/27 |
|  | Diisodecyl 4-cyclohexene-1,2-dicarboxylate | 15.37 | 450.3711 | 450 | C28H50O4 | 141/57/71/43/85 |
|  | Dodecyl cis-9,10-epoxyoctadecanoate | 15.48 | 466.4388 | 466 | C30H58O3 | 155/55/71/97/41 |
|  | Heneicosane, 11-cyclopentyl- | 13.00 | 364.4071 | 364 | C26H52 | 57/43/41/83/97 |
|  | Heptadecane, 2,6,10,14-tetramethyl- | 11.20 | 296.3445 | 296 | C21H44 | 57/43/71/41/85 |
|  | Heptadecanoic acid, heptadecyl ester | 18.53 | 508.5222 | 508 | C34H68O2 | 57/71/97/111/125 |
|  | Heptafluorobutyric acid, undecyl ester | 13.01 | 368.1586 | 368 | C15H23F7O2 | 69/55/83/41/97 |
|  | Hexane, 2,2,5,5-tetramethyl- | 10.70 | 142.1722 | 142 | C10H22 | 57/71/41/43/27 |
|  | Methoxyacetic acid, 4-hexadecyl ester | 15.95 | 314.2822 | 314 | C19H38O3 | 57/71/45/85/41 |
|  | Myristoyl chloride | 13.04 | 246.1752 | 246 | C14H27ClO | 41/98/84/57/27 |
|  | N-methylene-n-octadecylamine | 14.08 | 281.3084 | 281 | C19H39N | 84/57/43/70/41 |
|  | Oxalic acid, hexadecyl hexyl ester | 15.24 | 398.3397 | 398 | C24H46O4 | 43/57/85/41/71 |
|  | Pentadecane, 1-methoxy-13-methyl- | 14.14 | 256.2767 | 256 | C17H36O | 70/45/57/97/41 |
|  | Pentafluoropropionic acid, tetradecyl ester | 13.67 | 360.2088 | 360 | C17H29F5O2 | 55/83/97/41/111 |
|  | Pentane, 2-isocyano-2,4,4-trimethyl- | 15.91 | 139.1362 | 139 | C9H17N | 57/56/97/41/27 |
|  | Piperidine, 3-methyl- | 10.40 | 99.1048 | 99 | C6H13N | 44/99/57/84/30 |
|  | Propane, 2-(ethenyloxy)- | 12.63 | 86.0732 | 86 | C5H10O | 43/41/86/27/58 |
|  | Pyrrolidine, 1-(1-oxo-10,12-octadecadienyl)-, (E,E)- | 25.18 | 333.3033 | 333 | C22H39NO | 43/70/113/41/85 |
|  | Sulfurous acid, 2-propyl undecyl ester | 25.27 | 278.1917 | 278 | C14H30O3S | 43/57/71/85/41 |
|  | Sulfurous acid, isohexyl 2-pentyl ester | 15.16 | 236.1447 | 236 | C11H24O3S | 85/43/71/41/69 |
|  | trans-Z-.alpha.-Bisabolene epoxide | 14.68 | 220.1828 | 220 | C15H24O | 41/109/93/57/79 |
|  | Triacontane, 1,30-dibromo- | 15.25 | 578.3064 | 578 | C30H60Br2 | 57/43/71/41/85 |
| NSRC | (2E)-Dodec-2-en-1-yl methyl ether | 10.38 | 198.1984 | 198 | C13H26O | 71/41/85/55/67 |
|  | (7-Methoxy-tetrazolo[1,5-a][1,3,5]triazin-5-yl)-hydrazine | 13.69 | 182.0666 | 182 | C4H6N8O | 83/69/125/42/182 |
|  | .alpha.-D-Xylofuranose, cyclic 1,2:3,5-bis(ethylboronate) | 14.72 | 225.7217 | 226 | C9H16B2O5 | 111/124/29/57/70 |
|  | 1,2-Oxathiane, 6-dodecyl-, 2,2-dioxide | 14.63 | 304.2073 | 304 | C16H32O3S | 55/69/41/83/98 |
|  | 1,3,2-Dioxaphosphorinane, 2-(2-fluoroethoxy)-5,5-dimethyl- | 15.09 | 196.0665 | 196 | C7H14FO3P | 41/56/47/129/83 |
|  | 1,8-Diazabicyclo[5,4,0]undecan-7-ol | 15.14 | 170.142 | 170 | C9H18N2O | 44/152/41/98/127 |
|  | 10-Methylundec-3-en-4-olide | 25.22 | 196.1464 | 196 | C12H20O2 | 55/41/98/39/70 |
|  | 1-Cyclohexanol, 2-(2-ethylbutyl)- | 25.19 | 184.1828 | 184 | C12H24O | 84/137/81/55/41 |
|  | 2-Bromononane | 7.85 | 206.067 | 206 | C9H19Br | 41/71/57/85/27 |
|  | 2-Dodecen-1-ol | 13.68 | 184.1828 | 184 | C12H24O | 57/41/82/96/138 |
|  | 2-Heptenoic acid | 13.16 | 128.0837 | 128 | C7H12O2 | 41/73/56/43/27 |
|  | 2H-Quinolizine-1-methanol, octahydro-, (1S-cis)- | 14.62 | 169.1467 | 169 | C10H19NO | 41/83/55/152/27 |
|  | 2-Undecene, 7-methyl- | 12.83 | 168.1879 | 168 | C12H24 | 43/69/41/70/111 |
|  | 3,4-Diethyl hexane | 12.13 | 142.1722 | 142 | C10H22 | 70/43/57/41/113 |
|  | 3-Heptafluorobutyroxypentadecane | 13.11 | 424.2213 | 424 | C19H31F7O2 | 41/69/83/97/111 |
|  | 4-Cyclohexylnonadecane | 15.32 | 350.3915 | 350 | C25H50 | 83/55/57/97/41 |
|  | 5-Methyl-Z-5-docosene | 15.10 | 322.3601 | 322 | C23H46 | 69/97/55/111/41 |
|  | 6-Nonenal, 3,7-dimethyl- | 14.94 | 168.1515 | 168 | C11H20O | 55/81/41/99/95 |
|  | Acetic acid, 4-morpholin-4-yl-cyclohex-3-enyl ester | 15.24 | 225.1365 | 225 | C12H19NO3 | 43/165/96/68/29 |
|  | Arginine | 15.25 | 174.1118 | 174 | C6H14N4O2 | 69/43/30/70/138 |
|  | Bicyclo[2.2.1]heptan-2-one, 5-(acetyloxy)-4,6,6-trimethyl-, endo- | 16.17 | 210.1256 | 210 | C12H18O3 | 43/168/69/109/96 |
|  | Bisnorallocholanic acid | 14.43 | 332.2716 | 332 | C22H36O2 | 41/81/67/107/95 |
|  | Cholestan-7-one, cyclic 1,2-ethanediyl acetal, (5.alpha.)- | 13.82 | 430.3813 | 430 | C29H50O2 | 43/41/125/99/57 |
|  | Cycloheptanone, 3-(3,3-dimethylbutyl)-5-methyl- | 12.69 | 210.1984 | 210 | C14H26O | 57/125/83/55/210 |
|  | Dihexylamine, N-nitro- | 10.37 | 230.1995 | 230 | C12H26N2O2 | 43/41/56/112/184 |
|  | Heneicosyl heptafluorobutyrate | 25.21 | 508.3152 | 508 | C25H43F7O2 | 57/43/97/83/41 |
|  | Heptacosyl heptafluorobutyrate | 13.72 | 592.4092 | 592 | C31H55F7O2 | 57/97/83/55/111 |
|  | Hexadecane | 13.59 | 254.2975 | 254 | C18H38 | 57/71/43/85/41 |
|  | Octadecane, 4-methyl- | 15.69 | 268.3132 | 268 | C19H40 | 43/57/71/41/85 |
|  | Oxalic acid, 6-ethyloct-3-yl hexyl ester | 13.11 | 314.2458 | 314 | C18H34O4 | 43/85/57/71/41 |
|  | Piperidine, 3-(bromomethyl)- | 25.25 | 177.0153 | 177 | C6H12BrN | 41/98/80/56/93 |
|  | Tetracosyl pentafluoropropionate | 14.84 | 500.3654 | 500 | C27H49F5O2 | 57/97/55/83/111 |
|  | Tetradecyl trifluoroacetate | 12.72 | 310.212 | 310 | C16H29F3O2 | 55/83/97/41/111 |
| NSRP | 1,1'-(1-Methyl-1,2-ethanediylidene)bis(cyanoacetohydrazide) | 16.35 | 234.0867 | 234 | C9H10N6O2 | 42/68/40/166/138 |
|  | 11-Tricosene | 25.21 | 322.3601 | 322 | C23H46 | 43/41/57/83/97 |
|  | 1-Acetoxy-p-menth-3-one | 25.21 | 212.1413 | 212 | C12H20O3 | 82/110/95/41/69 |
|  | 1-Butanone, 1-cyclohexyl-4,4-diethoxy- | 15.94 | 242.1882 | 242 | C14H26O3 | 85/103/83/55/41 |
|  | 1-Cyclohexanecarboxylic acid, 1-(4-methyl-2-oxopentyl)-2-oxo-, methyl ester | 14.51 | 254.1518 | 254 | C14H22O4 | 137/85/57/81/109 |
|  | 1-Decyloxy-2-nitrobenzene | 15.92 | 279.1835 | 279 | C16H25NO3 | 57/43/140/41/85 |
|  | 1-Ethynyl-3,trans(1,1-dimethylethyl)-4,cis-methoxycyclohexan-1-ol | 16.02 | 210.162 | 210 | C13H22O2 | 57/70/104/121/55 |
|  | 1-Heptanol, 3-methyl- | 10.35 | 130.1358 | 130 | C8H18O | 70/55/84/56/41 |
|  | 1R-Ethoxy-3-trans-methoxy-2-cis-methylcyclohexane | 16.21 | 172.1464 | 172 | C10H20O2 | 68/126/41/72/94 |
|  | 2,3,6-trimethylhept-5-en-1-ol | 13.06 | 156.1515 | 156 | C10H20O | 69/41/70/55/85 |
|  | 2,6,10,10-Tetramethyl-1-oxaspiro[4.5]decan-6-ol | 21.69 | 212.1777 | 212 | C13H24O2 | 85/126/43/41/69 |
|  | 2-Octenoic acid, 7-hydroxy-, ethyl ester | 19.00 | 186.1256 | 186 | C10H18O3 | 95/45/114/81/29 |
|  | 3-Decenoic acid, (E)- | 15.82 | 170.1307 | 170 | C10H18O2 | 41/69/27/110/81 |
|  | 3-tert-Butyl-5-chloro-2-hydroxybenzophenone | 14.68 | 288.0918 | 288 | C17H17ClO2 | 105/40/44/57/77 |
|  | 3-Tridecene, (E)- | 9.08 | 182.2035 | 182 | C13H26 | 41/55/69/70/27 |
|  | 3-Undecene, 8-methyl- | 14.85 | 168.1879 | 168 | C12H24 | 55/41/84/70/27 |
|  | 5-Octadecenal | 16.65 | 266.2611 | 266 | C18H34O | 57/43/83/84/41 |
|  | Androstane-6,17-dione, 3-hydroxy-, (3.beta.,5.alpha.)- | 14.48 | 304.2039 | 304 | C19H28O3 | 41/55/67/95/139 |
|  | Azacyclodecan-5-ol | 14.14 | 157.1467 | 157 | C9H19NO | 44/41/57/70/86 |
|  | Bicyclo[3.1.0]hexane-6-carboxylic acid, 1,4,4-trimethyl-2-oxo-, (1.alpha.,5.alpha.,6.alpha.)-(.+-.)- | 13.69 | 182.0943 | 182 | C10H14O3 | 113/137/70/98/182 |
|  | Butanoic acid, 2-bromo-, pentyl ester | 16.37 | 236.0412 | 236 | C9H17BrO2 | 42/70/41/79/87 |
|  | Cyclodecanone, oxime | 14.45 | 169.1467 | 169 | C10H19NO | 41/73/110/95/137 |
|  | E-10,13,13-Trimethyl-11-tetradecen-1-ol acetate | 14.94 | 296.2716 | 296 | C19H36O2 | 57/83/43/95/41 |
|  | E-8-Methyl-9-tetradecen-1-ol acetate | 25.19 | 268.2403 | 268 | C17H32O2 | 43/69/97/41/109 |
|  | Heptane, 2,2,3,3,5,6,6-heptamethyl- | 13.29 | 198.2349 | 198 | C14H30 | 57/85/41/71/99 |
|  | Ketone, 1-cyclohexen-1-yl methyl, semicarbazone | 13.72 | 181.1216 | 181 | C9H15N3O | 137/123/79/109/96 |
|  | Methyldicyanophosphine | 15.41 | 98.0034 | 98 | C3H3N2P | 27/28/71/57/98 |
|  | N-Hexadecylpyridinium bromide | 25.27 | 383.2189 | 383 | C21H38BrN | 43/57/41/71/135 |
|  | Nonane, 2,8-dimethyl-4-methylene- | 10.54 | 168.1879 | 168 | C12H24 | 56/43/83/41/27 |
|  | Octahydro-pyrano[3,2-b]pyran | 13.00 | 142.0994 | 142 | C8H14O2 | 71/43/142/41/100 |
|  | o-Toluic acid, 2-tetrahydrofurylmethyl ester | 15.37 | 220.1099 | 220 | C13H16O3 | 71/84/119/43/65 |
|  | Oxetane, 2-(1,1-dimethylethyl)-3-methyl- | 14.77 | 128.1201 | 128 | C8H16O | 41/55/70/57/113 |
|  | Sulfurous acid, octadecyl pentyl ester | 13.01 | 404.3326 | 404 | C23H48O3S | 71/43/57/41/85 |
|  | Tetracosa-2,6,10,14,18-pentaen-22-one, 2,6,10,15,19,23-hexamethyl-, all (E)- | 16.23 | 426.3864 | 426 | C30H50O | 69/71/43/153/41 |
|  | Tetrazolo[1,5-b]pyridazin-6-amine, N-tetrahydrofurfuryl- | 16.52 | 220.1074 | 220 | C9H12N6O | 43/67/41/84/137 |
|  | Tridecane, 6-cyclohexyl- | 14.42 | 266.2975 | 266 | C19H38 | 57/83/55/41/97 |
|  | Tridecane, 7-hexyl- | 13.16 | 268.3132 | 268 | C19H40 | 43/57/29/71/41 |
|  | Trihexadecyl borate | 15.33 | 734.5708 | 734 | C48H99BO3 | 57/43/83/97/41 |

LR - Landraces are described in Table 1. Rt – Retention time (min).

Supplementary Data 2a: Phytochemical compounds shared among all thirteen landraces.

| Phytochemical compound | Chemical formula | Chemical Group | Landrace | Rt(mins) | RPA (%) |
| --- | --- | --- | --- | --- | --- |
| 1-Dodecene | C12H24 | Monoterpene | BG-24 | 11.11 | 3.02 |
|  |  |  | BG-31 | 9.10 | 1.14 |
|  |  |  | BG-70 | 11.42 | 6.23 |
|  |  |  | BG-100/GC | 10.64 | 4.32 |
|  |  |  | DSI | 11.41 | 3.89 |
|  |  |  | ESC | 9.08 | 1.14 |
|  |  |  | KRI | 10.63 | 3.82 |
|  |  |  | KSC | 11.42 | 3.19 |
|  |  |  | KSP | 11.42 | 5.19 |
|  |  |  | NRC | 11.41 | 5.26 |
|  |  |  | NqSC | 10.83 | 4.74 |
|  |  |  | NSRC | 11.42 | 6.97 |
|  |  |  | NSRP | 11.42 | 4.90 |
| 2,3-Dimethyldodecane | C14H30 | Monoterpene | BG-24 | 10.58 | 3.73 |
|  |  |  | BG-31 | 12.90 | 2.85 |
|  |  |  | BG-70 | 16.29 | 3.56 |
|  |  |  | BG-100/GC | 12.77 | 2.18 |
|  |  |  | DSI | 13.23 | 1.94 |
|  |  |  | ESC | 14.52 | 4.12 |
|  |  |  | KRI | 13.7 | 1.82 |
|  |  |  | KSC | 12.48 | 1.09 |
|  |  |  | KSP | 13.39 | 1.96 |
|  |  |  | NRC | 12.56 | 2.01 |
|  |  |  | NqSC | 13.86 | 3.20 |
|  |  |  | NSRC | 13.69 | 4.00 |
|  |  |  | NSRP | 13.48 | 3.05 |
| E-15-Heptadecenal | C17H32O | Sesquiterpene | BG-24 | 16.52 | 7.49 |
|  |  |  | BG-31 | 15.86 | 6.24 |
|  |  |  | BG-70 | 15.19 | 11.41 |
|  |  |  | BG-100/GC | 15.09 | 8.86 |
|  |  |  | DSI | 17.06 | 4.74 |
|  |  |  | ESC | 15.95 | 6.82 |
|  |  |  | KRI | 15.95 | 6.96 |
|  |  |  | KSC | 16.00 | 4.94 |
|  |  |  | KSP | 15.46 | 7.47 |
|  |  |  | NRC | 13.52 | 10.28 |
|  |  |  | NqSC | 16.36 | 7.36 |
|  |  |  | NSRC | 16.44 | 9.41 |
|  |  |  | NSRP | 16.01 | 7.24 |
| Eicosane | C20H42 | Diterpene | BG-24 | 15.60 | 3.89 |
|  |  |  | BG-31 | 15.65 | 4.13 |
|  |  |  | BG-70 | 14.39 | 3.29 |
|  |  |  | BG-100/GC | 16.19 | 4.01 |
|  |  |  | DSI | 16.49 | 3.75 |
|  |  |  | ESC | 15.77 | 2.47 |
|  |  |  | KRI | 16.93 | 3.24 |
|  |  |  | KSC | 17.20 | 3.06 |
|  |  |  | KSP | 15.92 | 4.53 |
|  |  |  | NRC | 14.99 | 4.49 |
|  |  |  | NqSC | 17.99 | 2.25 |
|  |  |  | NSRC | 18.00 | 2.33 |
|  |  |  | NSRP | 16.99 | 3.02 |
| Tridecane, 6-propyl- | C16H34 | Sesquiterpene | BG-24 | 14.50 | 3.94 |
|  |  |  | BG-31 | 14.17 | 5.15 |
|  |  |  | BG-70 | 13.75 | 4.22 |
|  |  |  | BG-100/GC | 14.08 | 6.45 |
|  |  |  | DSI | 14.56 | 8.70 |
|  |  |  | ESC | 13.25 | 4.36 |
|  |  |  | KRI | 13.89 | 4.55 |
|  |  |  | KSC | 13.77 | 5.64 |
|  |  |  | KSP | 15.02 | 5.30 |
|  |  |  | NRC | 14.94 | 4.26 |
|  |  |  | NqSC | 14.48 | 3.57 |
|  |  |  | NSRC | 14.23 | 3.29 |
|  |  |  | NSRP | 13.42 | 3.66 |

Landraces are described in Table 1. Rt – Retention time (min) and RPA – Relative peak area (%).

Supplementary Data 2b: Phytochemical compounds shared among twelve landraces.

| Phytochemical compound | Chemical formula | Chemical Group | Landrace | Rt(mins) | RPA (%) |
| --- | --- | --- | --- | --- | --- |
| Octadecane | C20H42 | Diterpene | BG-24 | 13.09 | 4.89 |
|  |  |  | BG-31 | 14.64 | 4.72 |
|  |  |  | BG-70 | 15.13 | 4.76 |
|  |  |  | BG-100/GC | 15.68 | 3.76 |
|  |  |  | DSI | 15.68 | 3.70 |
|  |  |  | ESC | 14.62 | 4.15 |
|  |  |  | KRI | 15.67 | 4.11 |
|  |  |  | KSC | 14.08 | 3.27 |
|  |  |  | NRC | 15.67 | 4.31 |
|  |  |  | NqSC | 14.93 | 4.69 |
|  |  |  | NSRC | 15.13 | 4.75 |
|  |  |  | NSRP | 15.68 | 4.74 |
| Octadecane, 5-methyl- | C19H40 | Sesquiterpene | BG-24 | 12.98 | 1.16 |
|  |  |  | BG-31 | 15.02 | 0.65 |
|  |  |  | BG-100/GC | 14.74 | 0.93 |
|  |  |  | DSI | 16.13 | 1.62 |
|  |  |  | ESC | 14.37 | 0.65 |
|  |  |  | KRI | 16.33 | 0.36 |
|  |  |  | KSC | 14.40 | 1.30 |
|  |  |  | KSP | 15.74 | 2.38 |
|  |  |  | NRC | 15.36 | 0.61 |
|  |  |  | NqSC | 16.95 | 0.88 |
|  |  |  | NSRC | 18.19 | 1.11 |
|  |  |  | NSRP | 23.68 | 0.58 |
| Tetradecane, 4-methyl- | C15H32 | Sesquiterpene | BG-24 | 12.66 | 2.44 |
|  |  |  | BG-31 | 13.23 | 1.13 |
|  |  |  | BG-70 | 15.12 | 1.57 |
|  |  |  | BG-100/GC | 12.90 | 3.97 |
|  |  |  | DSI | 15.00 | 4.62 |
|  |  |  | ESC | 12.42 | 0.30 |
|  |  |  | KRI | 2.29 | 0.43 |
|  |  |  | KSC | 13.28 | 0.51 |
|  |  |  | NRC | 19.01 | 1.11 |
|  |  |  | NqSC | 10.50 | 0.97 |
|  |  |  | NSRC | 10.51 | 0.75 |
|  |  |  | NSRP | 15.74 | 3.13 |

Supplementary Data 2c: Phytochemical compounds shared among eleven landraces.

| Phytochemical compound | Chemical formula | Chemical Group | Landrace | Rt(mins) | RPA (%) |
| --- | --- | --- | --- | --- | --- |
| Eicosane, 10-methyl- | C21H44 | Diterpene | BG-24 | 14.04 | 1.76 |
|  |  |  | BG-70 | 14.03 | 1.27 |
|  |  |  | BG-100/GC | 12.58 | 2.48 |
|  |  |  | DSI | 13.59 | 7.27 |
|  |  |  | ESC | 14.03 | 1.74 |
|  |  |  | KRI | 16.04 | 1.24 |
|  |  |  | KSC | 13.53 | 1.09 |
|  |  |  | KSP | 12.89 | 4.33 |
|  |  |  | NqSC | 14.03 | 1.81 |
|  |  |  | NSRC | 12.88 | 2.39 |
|  |  |  | NSRP | 13.65 | 1.98 |
| Pentadecane, 2-methyl- | C16H34 | Sesquiterpene | BG-31 | 13.23 | 0.83 |
|  |  |  | BG-70 | 13.22 | 1.03 |
|  |  |  | BG-100/GC | 12.57 | 1.81 |
|  |  |  | ESC | 12.58 | 2.92 |
|  |  |  | KRI | 13.21 | 1.11 |
|  |  |  | KSC | 13.21 | 0.86 |
|  |  |  | KSP | 12.57 | 2.47 |
|  |  |  | NRC | 13.22 | 0.95 |
|  |  |  | NqSC | 12.78 | 1.99 |
|  |  |  | NSRC | 12.57 | 2.73 |
|  |  |  | NSRP | 13.59 | 6.26 |
| Tridecane, 7-propyl- | C16H34 | Sesquiterpene | BG-24 | 13.59 | 8.07 |
|  |  |  | BG-70 | 19.03 | 1.69 |
|  |  |  | BG-100/GC | 13.22 | 1.07 |
|  |  |  | DSI | 12.58 | 2.97 |
|  |  |  | KRI | 13.59 | 2.44 |
|  |  |  | KSC | 13.21 | 0.87 |
|  |  |  | KSP | 13.58 | 7.58 |
|  |  |  | NRC | 13.59 | 6.10 |
|  |  |  | NqSC | 13.22 | 1.22 |
|  |  |  | NSRC | 14.08 | 5.61 |
|  |  |  | NSRP | 12.57 | 2.17 |

Landraces are described in Table 1. Rt – Retention time (min) and RPA – Relative peak area (%)

Supplementary Data 2d: Phytochemical compounds shared among ten landraces.

| Phytochemical compound | Chemical formula | Chemical Group | Landrace | Rt(mins) | RPA (%) |
| --- | --- | --- | --- | --- | --- |
| 1-Heptadecene | C17H34 | Sesquiterpene | BG-24 | 14.57 | 10.85 |
|  |  |  | BG-31 | 16.22 | 2.94 |
|  |  |  | BG-70 | 18.91 | 6.10 |
|  |  |  | BG-100/GC | 13.52 | 12.99 |
|  |  |  | KRI | 13.52 | 12.40 |
|  |  |  | KSC | 15.60 | 6.25 |
|  |  |  | KSP | 25.20 | 0.30 |
|  |  |  | NRC | 15.61 | 8.55 |
|  |  |  | NqSC | 16.21 | 6.73 |
|  |  |  | NSRC | 16.22 | 8.16 |
| Hexadecane, 4-methyl- | C17H36 | Sesquiterpene | BG-31 | 12.86 | 1.08 |
|  |  |  | BG-70 | 12.86 | 0.97 |
|  |  |  | BG-100/GC | 12.86 | 1.37 |
|  |  |  | ESC | 15.17 | 1.16 |
|  |  |  | KRI | 15.15 | 1.33 |
|  |  |  | KSC | 12.84 | 0.76 |
|  |  |  | KSP | 15.15 | 1.16 |
|  |  |  | NRC | 14.13 | 0.68 |
|  |  |  | NqSC | 13.74 | 2.08 |
|  |  |  | NSRP | 14.21 | 1.20 |
| Tetradecane, 5-methyl- | C15H32 | Sesquiterpene | BG-24 | 15.34 | 0.30 |
|  |  |  | BG-31 | 15.62 | 1.46 |
|  |  |  | BG-70 | 12.90 | 3.39 |
|  |  |  | BG-100/GC | 13.31 | 0.63 |
|  |  |  | DSI | 12.90 | 4.21 |
|  |  |  | KSC | 12.41 | 0.55 |
|  |  |  | KSP | 16.33 | 2.42 |
|  |  |  | NRC | 15.19 | 0.99 |
|  |  |  | NqSC | 19.67 | 1.65 |
|  |  |  | NSRP | 19.03 | 1.40 |
| Trichloroacetic acid, hexadecyl ester | C18H33Cl3O2 | Fatty acid | BG-24 | 12.82 | 1.24 |
|  |  |  | BG-31 | 13.79 | 1.38 |
|  |  |  | BG-70 | 13.86 | 1.35 |
|  |  |  | BG-100/GC | 14.13 | 1.38 |
|  |  |  | DSI | 14.84 | 2.47 |
|  |  |  | KRI | 13.77 | 1.14 |
|  |  |  | KSC | 12.72 | 0.50 |
|  |  |  | KSP | 16.87 | 1.59 |
|  |  |  | NRC | 12.85 | 0.85 |
|  |  |  | NqSC | 11.90 | 0.99 |

Supplementary Data 2e: Phytochemical compounds shared among nine landraces.

| Phytochemical compound | Chemical formula | Chemical Group | Landrace | Rt(mins) | RPA (%) |
| --- | --- | --- | --- | --- | --- |
| 1-Undecene, 7-methyl- | C12H24 | Monoterpene | BG-24 | 10.40 | 1.50 |
|  |  |  | BG-31 | 10.35 | 0.83 |
|  |  |  | BG-100/GC | 12.02 | 1.52 |
|  |  |  | ESC | 10.45 | 0.53 |
|  |  |  | KRI | 10.40 | 1.01 |
|  |  |  | KSP | 10.33 | 1.86 |
|  |  |  | NRC | 10.44 | 0.62 |
|  |  |  | NqSC | 12.09 | 0.85 |
|  |  |  | NSRP | 10.34 | 1.02 |
| Decane, 3,7-dimethyl- | C13H28 | Monoterpene | BG-31 | 12.59 | 2.44 |
|  |  |  | BG-70 | 12.08 | 5.67 |
|  |  |  | BG-100/GC | 10.57 | 2.62 |
|  |  |  | DSI | 11.04 | 2.68 |
|  |  |  | ESC | 11.71 | 1.27 |
|  |  |  | KRI | 12.27 | 2.49 |
|  |  |  | KSC | 10.57 | 1.04 |
|  |  |  | KSP | 10.57 | 3.29 |
|  |  |  | NqSC | 11.81 | 2.36 |
| Dodecane, 2,6,11-trimethyl- | C15H32 | Sesquiterpene | BG-24 | 12.58 | 2.92 |
|  |  |  | BG-70 | 10.57 | 2.29 |
|  |  |  | BG-100/GC | 12.86 | 1.11 |
|  |  |  | ESC | 12.14 | 0.46 |
|  |  |  | KRI | 14.53 | 1.99 |
|  |  |  | KSC | 10.50 | 0.41 |
|  |  |  | NqSC | 12.85 | 2.25 |
|  |  |  | NSRC | 10.58 | 2.40 |
|  |  |  | NSRP | 13.75 | 0.58 |
| Heneicosane, 11-(1-ethylpropyl)- | C26H54 | Sesterterpene | BG-70 | 10.02 | 0.92 |
|  |  |  | DSI | 15.74 | 2.31 |
|  |  |  | ESC | 14.01 | 1.93 |
|  |  |  | KRI | 10.01 | 1.05 |
|  |  |  | KSC | 12.77 | 0.26 |
|  |  |  | KSP | 10.01 | 0.86 |
|  |  |  | NRC | 17.98 | 1.69 |
|  |  |  | NqSC | 15.59 | 1.09 |
|  |  |  | NSRC | 12.86 | 1.06 |
| Undecane, 2-methyl- | C12H26 | Monoterpenes | BG-24 | 12.36 | 2.28 |
|  |  |  | BG-70 | 13.22 | 0.69 |
|  |  |  | DSI | 13.22 | 1.22 |
|  |  |  | ESC | 13.40 | 3.80 |
|  |  |  | KRI | 11.49 | 4.26 |
|  |  |  | KSP | 13.40 | 4.66 |
|  |  |  | NRC | 10.56 | 1.46 |
|  |  |  | NqSC | 12.89 | 6.10 |
|  |  |  | NSRP | 13.40 | 3.29 |

Landraces are described in Table 1. Rt – Retention time (min) and RPA – Relative peak area (%).

Supplementary Data 2f: Phytochemical compounds shared among eight landraces.

| Phytochemical compound | Chemical formula | Chemical Group | Landrace | Rt(mins) | RPA (%) |
| --- | --- | --- | --- | --- | --- |
| 11-Methyldodecanol | C13H28O | Monoterpene | BG-24 | 14.85 | 1.04 |
|  |  |  | BG-70 | 10.34 | 1.47 |
|  |  |  | DSI | 10.34 | 0.58 |
|  |  |  | ESC | 16.23 | 0.69 |
|  |  |  | KSP | 10.44 | 1.43 |
|  |  |  | NRC | 10.44 | 0.66 |
|  |  |  | NqSC | 18.78 | 0.85 |
|  |  |  | NSRC | 10.44 | 0.88 |
| Heptadecane, 2,3-dimethyl- | C19H40 | Sesquiterpene | BG-24 | 14.21 | 1.66 |
|  |  |  | BG-31 | 11.30 | 1.27 |
|  |  |  | BG-70 | 14.21 | 0.84 |
|  |  |  | BG-100/GC | 12.89 | 2.39 |
|  |  |  | ESC | 15.25 | 2.13 |
|  |  |  | KRI | 14.76 | 1.38 |
|  |  |  | KSC | 13.93 | 4.30 |
|  |  |  | NRC | 13.54 | 3.02 |
| Heptadecyl heptafluorobutyrate | C21H35F7O2 | Ester | BG-24 | 14.24 | 1.30 |
|  |  |  | BG-31 | 14.85 | 1.36 |
|  |  |  | BG-70 | 14.85 | 0.94 |
|  |  |  | KRI | 13.94 | 0.75 |
|  |  |  | KSP | 14.84 | 0.89 |
|  |  |  | NRC | 14.84 | 1.12 |
|  |  |  | NqSC | 25.21 | 1.28 |
|  |  |  | NSRP | 13.01 | 1.10 |
| Hexadecane, 5-butyl- | C20H42 | Diterpene | BG-24 | 14.22 | 1.20 |
|  |  |  | BG-31 | 12.86 | 1.33 |
|  |  |  | ESC | 15.34 | 1.20 |
|  |  |  | KSC | 15.32 | 1.63 |
|  |  |  | KSP | 15.32 | 1.09 |
|  |  |  | NRC | 15.32 | 1.07 |
|  |  |  | NqSC | 15.32 | 1.10 |
|  |  |  | NSRP | 17.12 | 1.66 |
| Nonane, 5-(2-methylpropyl)- | C13H28 | Monoterpene | BG-24 | 11.44 | 0.79 |
|  |  |  | DSI | 15.74 | 0.70 |
|  |  |  | ESC | 12.86 | 0.58 |
|  |  |  | KRI | 10.72 | 0.36 |
|  |  |  | KSC | 14.77 | 1.19 |
|  |  |  | KSP | 10.01 | 0.76 |
|  |  |  | NSRC | 13.79 | 1.05 |
|  |  |  | NSRP | 10.58 | 2.24 |
| Pentadecane, 3-methyl- | C16H34 | Sesquiterpene | BG-31 | 13.98 | 0.77 |
|  |  |  | BG-100/GC | 13.29 | 0.36 |
|  |  |  | DSI | 13.29 | 0.83 |
|  |  |  | ESC | 13.75 | 0.91 |
|  |  |  | KRI | 13.29 | 0.55 |
|  |  |  | KSC | 13.21 | 0.86 |
|  |  |  | NRC | 13.28 | 0.51 |
|  |  |  | NqSC | 13.29 | 0.57 |
| Tetradecane, 2-methyl- | C15H32 | Sesquiterpene | BG-24 | 12.04 | 0.82 |
|  |  |  | BG-31 | 12.81 | 1.35 |
|  |  |  | BG-100/GC | 10.51 | 0.87 |
|  |  |  | DSI | 13.58 | 7.14 |
|  |  |  | ESC | 10.50 | 0.61 |
|  |  |  | KRI | 10.50 | 0.68 |
|  |  |  | KSC | 10.50 | 0.55 |
|  |  |  | NqSC | 10.50 | 0.85 |

Landraces are described in Table 1. Rt – Retention time (min) and RPA – Relative peak area (%).

Supplementary Data 2g: Phytochemical compounds shared among seven landraces.

| Phytochemical compound | Chemical formula | Chemical Group | Landrace | Rt(mins) | RPA (%) |
| --- | --- | --- | --- | --- | --- |
| 1-Hexanol, 5-methyl-2-(1-methylethyl)- | C10H22O | Monoterpene | BG-31 | 13.65 | 1.66 |
|  |  |  | ESC | 13.01 | 1.25 |
|  |  |  | KSC | 10.02 | 0.39 |
|  |  |  | NRC | 25.19 | 1.16 |
|  |  |  | NqSC | 12.12 | 0.68 |
|  |  |  | NSRC | 15.76 | 1.79 |
|  |  |  | NSRP | 15.75 | 2.78 |
| 1-Pentadecene | C15H30 | Sesquiterpene | BG-24 | 11.43 | 4.99 |
|  |  |  | BG-70 | 11.42 | 7.88 |
|  |  |  | BG-100/GC | 11.42 | 6.72 |
|  |  |  | ESC | 10.92 | 3.73 |
|  |  |  | KRI | 11.40 | 6.22 |
|  |  |  | KSC | 11.42 | 4.30 |
|  |  |  | NqSC | 15.15 | 1.36 |
| 5,5-Diethyltridecane | C17H36 | Sesquiterpene | BG-24 | 12.86 | 1.47 |
|  |  |  | BG-70 | 12.86 | 1.42 |
|  |  |  | DSI | 12.86 | 1.81 |
|  |  |  | KRI | 14.27 | 0.79 |
|  |  |  | KSC | 23.65 | 1.67 |
|  |  |  | NSRC | 15.68 | 2.42 |
|  |  |  | NSRP | 12.86 | 0.86 |
| Hexadecen-1-ol, trans-9- | C16H32O | Fatty alcohol | BG-24 | 16.02 | 2.56 |
|  |  |  | BG-31 | 11.43 | 4.05 |
|  |  |  | BG-70 | 11.42 | 9.29 |
|  |  |  | ESC | 12.59 | 5.64 |
|  |  |  | KSC | 13.51 | 4.38 |
|  |  |  | NqSC | 13.52 | 14.03 |
|  |  |  | NSRC | 13.52 | 15.67 |

Landraces are described in Table 1. Rt – Retention time (min) and RPA – Relative peak area (%).

Supplementary Data 2h: Phytochemical compounds shared among six landraces.

| Phytochemical compound | Chemical formula | Chemical Group | Landrace | Rt(mins) | RPA (%) |
| --- | --- | --- | --- | --- | --- |
| 1-Heptanol, 2-propyl- | C10H22O | Fatty alcohol | BG-70 | 10.01 | 1.39 |
|  |  |  | DSI | 14.22 | 2.12 |
|  |  |  | KRI | 12.18 | 0.93 |
|  |  |  | KSP | 13.10 | 1.12 |
|  |  |  | NSRC | 10.02 | 1.00 |
|  |  |  | NSRP | 10.01 | 0.90 |
| 1-Octadecanesulphonyl chloride | C18H37ClO2S | Sulfonyl halide | BG-31 | 14.60 | 0.77 |
|  |  |  | BG-100/GC | 15.26 | 1.59 |
|  |  |  | ESC | 17.07 | 0.98 |
|  |  |  | KSC | 17.55 | 0.86 |
|  |  |  | NqSC | 15.09 | 0.92 |
|  |  |  | NSRP | 15.10 | 1.23 |
| 2,6-Dimethyldecane | C12H26 | Monoterpene | BG-100/GC | 10.58 | 1.69 |
|  |  |  | KRI | 10.57 | 2.13 |
|  |  |  | KSC | 10.56 | 2.17 |
|  |  |  | KSP | 15.08 | 1.04 |
|  |  |  | NqSC | 10.64 | 1.66 |
|  |  |  | NSRC | 13.22 | 1.13 |
| 2-Isopropyl-5-methyl-1-heptanol | C11H24O | Monoterpene | BG-24 | 10.45 | 1.13 |
|  |  |  | BG-31 | 13.01 | 0.96 |
|  |  |  | BG-70 | 15.17 | 0.72 |
|  |  |  | BG-100/GC | 13.01 | 1.14 |
|  |  |  | KRI | 13.92 | 0.87 |
|  |  |  | NRC | 15.15 | 0.89 |
| Heptadecane, 2-methyl- | C18H38 | Sesquiterpene | BG-24 | 14.28 | 0.63 |
|  |  |  | BG-31 | 12.86 | 1.08 |
|  |  |  | BG-70 | 12.86 | 0.97 |
|  |  |  | ESC | 14.28 | 0.69 |
|  |  |  | NRC | 14.27 | 0.71 |
|  |  |  | NqSC | 12.85 | 1.11 |
| Tetradecane, 4-ethyl- | C16H34 | Sesquiterpene | ESC | 12.35 | 1.83 |
|  |  |  | KRI | 13.59 | 7.98 |
|  |  |  | KSC | 15.67 | 4.22 |
|  |  |  | KSP | 15.34 | 5.17 |
|  |  |  | NRC | 13.81 | 0.96 |
|  |  |  | NqSC | 13.32 | 1.64 |
| Undecane, 4-methyl- | C12H26 | Monoterpene | BG-31 | 10.58 | 2.77 |
|  |  |  | ESC | 10.58 | 3.39 |
|  |  |  | KRI | 15.75 | 1.24 |
|  |  |  | KSP | 12.85 | 0.97 |
|  |  |  | NqSC | 13.15 | 2.57 |
|  |  |  | NSRC | 10.57 | 2.55 |

Landraces are described in Table 1. Rt – Retention time (min) and RPA – Relative peak area (%).

Supplementary Data 2i: Phytochemical compounds shared among five landraces.

| Phytochemical compound | Chemical formula | Chemical Group | Landrace | Rt(mins) | RPA (%) |
| --- | --- | --- | --- | --- | --- |
| 1-Dodecanol, 2-hexyl- | C18H38O | Sesquiterpene | KRI | 14.83 | 1.05 |
|  |  |  | BG-31 | 13.11 | 0.73 |
|  |  |  | BG-100/GC | 11.15 | 1.03 |
|  |  |  | NqSC | 14.84 | 1.28 |
|  |  |  | NSRC | 12.73 | 1.10 |
| 1-Hexacosene | C26H52 | Sesterterpene | BG-31 | 13.01 | 1.11 |
|  |  |  | BG-100/GC | 15.25 | 1.84 |
|  |  |  | ESC | 15.91 | 1.85 |
|  |  |  | NRC | 13.00 | 1.29 |
|  |  |  | NSRC | 11.75 | 0.88 |
| 1-Nonene, 4,6,8-trimethyl- | C12H24 | Monoterpene | ESC | 13.62 | 0.72 |
|  |  |  | KSC | 10.34 | 1.39 |
|  |  |  | NqSC | 10.35 | 1.84 |
|  |  |  | NRC | 10.35 | 1.10 |
|  |  |  | NSRC | 10.35 | 1.68 |
| 2-Hexyl-1-octanol | C14H30O | Monoterpene | BG-24 | 15.25 | 1.11 |
|  |  |  | BG-70 | 12.73 | 0.75 |
|  |  |  | KRI | 13.00 | 0.83 |
|  |  |  | KSC | 15.24 | 1.37 |
|  |  |  | NRC | 13.10 | 0.75 |
| 2-Hexyldecyl acetate | C18H36O2 | Ester | BG-31 | 10.45 | 0.96 |
|  |  |  | BG-70 | 12.73 | 1.01 |
|  |  |  | KRI | 12.86 | 1.08 |
|  |  |  | NqSC | 12.72 | 1.23 |
|  |  |  | NRC | 14.83 | 1.03 |
| 3-Eicosene, (E)- | C20H40 | Diterpene | KRI | 10.44 | 1.16 |
|  |  |  | KSP | 13.00 | 1.18 |
|  |  |  | NqSC | 10.44 | 0.68 |
|  |  |  | NSRC | 18.94 | 3.36 |
|  |  |  | NSRP | 12.72 | 1.01 |
| 3-Tetradecene, (E)- | C14H28 | Monoterpene | BG-24 | 11.43 | 6.06 |
|  |  |  | BG-31 | 11.43 | 2.13 |
|  |  |  | KRI | 9.10 | 1.18 |
|  |  |  | NRC | 9.08 | 0.77 |
|  |  |  | NSRC | 11.42 | 8.62 |
| 4-Isopropyl-1,3-cyclohexanedione | C9H14O2 | Monoterpene | BG-100/GC | 12.82 | 0.37 |
|  |  |  | DSI | 12.72 | 1.15 |
|  |  |  | ESC | 14.84 | 1.22 |
|  |  |  | KRI | 16.57 | 0.54 |
|  |  |  | NSRC | 13.01 | 0.86 |
| Decane, 2-methyl- | C11H24 | Monoterpene | BG-31 | 11.50 | 1.29 |
|  |  |  | ESC | 17.13 | 1.05 |
|  |  |  | KSC | 12.12 | 0.38 |
|  |  |  | NRC | 11.50 | 3.01 |
|  |  |  | NSRC | 11.49 | 4.08 |
| Dodecane, 4-methyl- | C13H28 | Monoterpene | BG-31 | 12.86 | 1.06 |
|  |  |  | BG-70 | 15.69 | 2.69 |
|  |  |  | KSC | 10.72 | 0.25 |
|  |  |  | NqSC | 11.76 | 0.64 |
|  |  |  | NSRP | 12.86 | 0.90 |
| E-14-Hexadecenal | C16H30O | Sesquiterpene | BG-24 | 13.53 | 10.83 |
|  |  |  | BG-31 | 13.53 | 6.13 |
|  |  |  | ESC | 18.93 | 3.63 |
|  |  |  | KSC | 18.91 | 1.50 |
|  |  |  | KSP | 13.52 | 9.21 |
| Eicosane, 2,4-dimethyl- | C22H46 | Diterpene | BG-31 | 23.69 | 1.68 |
|  |  |  | KRI | 23.68 | 1.57 |
|  |  |  | KSC | 14.07 | 0.79 |
|  |  |  | KSP | 10.50 | 0.62 |
|  |  |  | NRC | 23.65 | 1.40 |
| Heptadecane, 4-methyl- | C18H38 | Sesquiterpene | BG-31 | 14.77 | 0.76 |
|  |  |  | ESC | 15.17 | 1.16 |
|  |  |  | KRI | 15.15 | 1.33 |
|  |  |  | KSP | 15.15 | 1.16 |
|  |  |  | NSRC | 12.86 | 0.86 |
| Heptadecane, 9-hexyl- | C23H48 | Diterpene | BG-31 | 15.10 | 1.75 |
|  |  |  | BG-70 | 15.25 | 1.20 |
|  |  |  | KSC | 13.33 | 0.90 |
|  |  |  | NRC | 15.23 | 1.35 |
|  |  |  | NSRC | 14.21 | 0.98 |
| Naphthalene, decahydro-1,4-dimethoxy-, (1.alpha.,4.alpha.,4a.alpha.,8a.beta.)- | C12H22O2 | Monoterpene | BG-31 | 16.73 | 0.86 |
|  |  |  | KRI | 18.98 | 0.43 |
|  |  |  | KSC | 14.87 | 0.47 |
|  |  |  | NRC | 16.33 | 0.17 |
|  |  |  | NSRC | 14.51 | 0.35 |
| Octane, 2,3,3-trimethyl- | C11H24 | Monoterpene | BG-24 | 12.36 | 2.23 |
|  |  |  | DSI | 12.57 | 0.43 |
|  |  |  | KRI | 11.48 | 2.42 |
|  |  |  | KSC | 11.48 | 2.72 |
|  |  |  | KSP | 11.49 | 3.26 |
| Oxalic acid, octadecyl propyl ester | C23H44O4 | Ester | BG-24 | 11.74 | 1.45 |
|  |  |  | ESC | 10.34 | 1.08 |
|  |  |  | KRI | 13.10 | 0.92 |
|  |  |  | KSP | 12.72 | 1.23 |
|  |  |  | NqSC | 12.72 | 0.61 |
| Oxalic acid, propyl tridecyl ester | C18H34O4 | Ester | KRI | 15.25 | 0.80 |
|  |  |  | KSC | 12.85 | 0.99 |
|  |  |  | KSP | 15.09 | 1.46 |
|  |  |  | NSRC | 15.26 | 0.79 |
|  |  |  | NSRP | 13.10 | 0.74 |
| Pentadecane, 2,6,10-trimethyl- | C18H38 | Sesquiterpene | BG-31 | 14.04 | 1.86 |
|  |  |  | DSI | 14.03 | 2.74 |
|  |  |  | ESC | 14.04 | 2.35 |
|  |  |  | KRI | 14.02 | 1.68 |
|  |  |  | NRC | 14.02 | 1.71 |
| Tetradecane | C14H30 | Monoterpene | BG-24 | 11.50 | 2.90 |
|  |  |  | BG-70 | 11.49 | 3.87 |
|  |  |  | DSI | 11.49 | 0.86 |
|  |  |  | ESC | 11.50 | 2.31 |
|  |  |  | KSC | 11.50 | 2.92 |
| Trifluoroacetoxy hexadecane | C18H33F3O2 | Ester (Fluoric acid) | BG-24 | 12.73 | 1.86 |
|  |  |  | BG-70 | 12.72 | 0.79 |
|  |  |  | BG-100/GC | 12.72 | 0.70 |
|  |  |  | ESC | 12.72 | 0.38 |
|  |  |  | KSC | 12.71 | 1.26 |
| Undecane, 2,10-dimethyl- | C13H28 | Monoterpene | BG-100/GC | 11.50 | 4.74 |
|  |  |  | KRI | 11.49 | 2.51 |
|  |  |  | KSC | 15.67 | 2.67 |
|  |  |  | KSP | 11.49 | 2.90 |
|  |  |  | NSRP | 11.49 | 2.79 |

Landraces are described in Table 1. Rt – Retention time (min) and RPA – Relative peak area (%).

Supplementary Data 2j: Phytochemical compounds shared among four landraces.

| Phytochemical compound | Chemical formula | Chemical Group | Landrace | Rt(mins) | RPA (%) |
| --- | --- | --- | --- | --- | --- |
| 1-Decanol, 2-octyl- | C18H38O | Sesquiterpene | ESC | 15.25 | 1.36 |
|  |  |  | NqSC | 13.01 | 2.27 |
|  |  |  | NSRC | 13.64 | 0.95 |
|  |  |  | NSRP | 12.72 | 1.35 |
| 1-Octadecene | C18H36 | Sesquiterpene | BG-31 | 17.77 | 0.58 |
|  |  |  | BG-70 | 18.93 | 4.46 |
|  |  |  | DSI | 15.62 | 4.07 |
|  |  |  | KSP | 18.89 | 4.40 |
| 1-Tricosene | C23H46 | Diterpene | KRI | 13.85 | 0.49 |
|  |  |  | KSP | 25.18 | 1.24 |
|  |  |  | NqSC | 14.84 | 1.15 |
|  |  |  | NSRC | 12.72 | 1.24 |
| 2-Piperidinone, N-[4-bromo-n-butyl]- | C9H16BrNO | Cyclic amide | KRI | 13.32 | 0.15 |
|  |  |  | ESC | 16.54 | 0.61 |
|  |  |  | KSC | 13.79 | 0.70 |
|  |  |  | NqSC | 15.69 | 1.39 |
| 4-Trifluoroacetoxyhexadecane | C18H33F3O2 | Ester (Fluoric acid) | BG-24 | 10.34 | 1.43 |
|  |  |  | KSC | 12.81 | 0.44 |
|  |  |  | KSP | 10.43 | 0.84 |
|  |  |  | NSRC | 10.45 | 1.16 |
| Decane, 4-ethyl- | C16H34 | Sesquiterpene | BG-31 | 10.58 | 2.71 |
|  |  |  | BG-100/GC | 15.68 | 3.24 |
|  |  |  | NRC | 11.70 | 1.22 |
|  |  |  | NSRC | 14.66 | 0.90 |
| Decane, 5-ethyl-5-methyl- | C13H28 | Monoterpene | BG-31 | 12.42 | 0.49 |
|  |  |  | BG-70 | 10.72 | 0.59 |
|  |  |  | BG-100/GC | 10.02 | 0.88 |
|  |  |  | NSRC | 10.51 | 0.75 |
| Dichloroacetic acid, 2-pentadecyl ester | C17H32Cl2O2 | Ester | KSP | 9.15 | 2.91 |
|  |  |  | KSC | 15.52 | 0.69 |
|  |  |  | NqSC | 13.10 | 1.06 |
|  |  |  | NSRC | 9.17 | 2.00 |
| Hexadecane, 3-methyl- | C17H36 | Sesquiterpene | KRI | 14.27 | 0.91 |
|  |  |  | KSP | 14.27 | 0.40 |
|  |  |  | NqSC | 14.27 | 0.69 |
|  |  |  | NSRP | 14.28 | 0.78 |
| Hexadecane, 7,9-dimethyl- | C18H38 | Sesquiterpene | BG-31 | 12.90 | 3.59 |
|  |  |  | BG-70 | 18.00 | 2.01 |
|  |  |  | NqSC | 17.99 | 2.34 |
|  |  |  | NSRP | 12.89 | 2.78 |
| Nonahexacontanoic acid | C69H138O2 | Fatty acid | BG-31 | 12.69 | 0.30 |
|  |  |  | BG-70 | 23.69 | 1.74 |
|  |  |  | NqSC | 11.57 | 0.44 |
|  |  |  | NSRP | 25.24 | 0.60 |
| Nonane, 1-iodo- | C9H19I | Hemiterpene | BG-24 | 10.51 | 1.02 |
|  |  |  | ESC | 10.57 | 1.62 |
|  |  |  | KRI | 12.85 | 0.97 |
|  |  |  | KSP | 10.57 | 3.22 |
| Octadecane, 2-methyl- | C19H40 | Sesquiterpene | ESC | 15.33 | 1.38 |
|  |  |  | KRI | 15.31 | 1.36 |
|  |  |  | NRC | 17.16 | 1.09 |
|  |  |  | NSRP | 17.11 | 2.00 |
| Octadecane, 5,14-dibutyl- | C26H54 | Sesterterpene | BG-31 | 10.51 | 0.55 |
|  |  |  | BG-100/GC | 12.13 | 0.68 |
|  |  |  | ESC | 19.42 | 0.85 |
|  |  |  | NRC | 14.77 | 0.56 |
| Sulfurous acid, 2-propyl tridecyl ester | C16H34O3S | Ester | BG-24 | 14.09 | 0.37 |
|  |  |  | NRC | 12.25 | 0.40 |
|  |  |  | NqSC | 14.20 | 1.17 |
|  |  |  | NSRC | 15.04 | 0.61 |
| Sulfurous acid, octadecyl 2-propyl ester | C21H44O3S | Ester | BG-24 | 13.01 | 1.23 |
|  |  |  | KRI | 16.65 | 0.45 |
|  |  |  | NqSC | 15.75 | 0.69 |
|  |  |  | NSRC | 14.85 | 0.83 |
| Tetradecane, 2,6,10-trimethyl- | C17H36 | Sesquiterpene | BG-31 | 14.29 | 1.07 |
|  |  |  | BG-100/GC | 13.21 | 0.82 |
|  |  |  | KSC | 13.64 | 1.54 |
|  |  |  | NSRP | 23.68 | 2.12 |
| Tetrapentacontane, 1,54-dibromo- | C54H108Br2 | Polyterpene | BG-31 | 18.03 | 0.42 |
|  |  |  | BG-70 | 23.93 | 1.00 |
|  |  |  | NRC | 13.46 | 0.58 |
|  |  |  | NSRC | 13.29 | 0.64 |
| Tridecane, 4,8-dimethyl- | C15H32 | Sesquiterpene | BG-100/GC | 11.00 | 1.64 |
|  |  |  | KSC | 12.57 | 2.19 |
|  |  |  | NqSC | 12.57 | 3.47 |
|  |  |  | NRC | 14.03 | 2.60 |
| 2-methyloctacosane | C29H60 | Sesterterpene | BG-31 | 17.12 | 1.25 |
|  |  |  | ESC | 15.25 | 1.36 |
|  |  |  | KSP | 16.09 | 1.83 |
|  |  |  | NSRP | 15.24 | 1.21 |

Landraces are described in Table 1. Rt – Retention time (min) and RPA – Relative peak area (%).

Supplementary Data 2k: Phytochemical compounds shared among three landraces.

| Phytochemical compound | Chemical formula | Chemical Group | Landrace | Rt(mins) | RPA (%) |
| --- | --- | --- | --- | --- | --- |
| 1,3-Cyclohexanedione, 2-butyl-2-methyl- | C11H18O2 | Cyclic ketone | BG-100/GC | 11.59 | 0.22 |
|  |  |  | ESC | 10.34 | 0.59 |
|  |  |  | NRC | 15.40 | 0.32 |
| 1-Decanol, 2-hexyl- | C16H34O | Sesquiterpene | BG-31 | 13.11 | 0.73 |
|  |  |  | ESC | 12.72 | 0.78 |
|  |  |  | NSRC | 12.73 | 1.10 |
| 1-Oxaspiro[2.5]octan-4-one, 2,2,6-trimethyl-, trans- | C10H16O2 | Monoterpene | BG-100/GC | 16.02 | 0.17 |
|  |  |  | KSC | 14.51 | 1.05 |
|  |  |  | NqSC | 15.39 | 0.44 |
| 1-Oxaspiro[4.4]nonan-4-one, 2-isopropyl- | C11H18O2 | Monoterpene | KRI | 12.68 | 0.45 |
|  |  |  | KSC | 13.43 | 0.10 |
|  |  |  | KSP | 14.93 | 0.48 |
| 1-Undecene, 4-methyl- | C12H24 | Monoterpene | BG-70 | 10.57 | 2.88 |
|  |  |  | KSC | 12.17 | 0.44 |
|  |  |  | NRC | 10.02 | 0.93 |
| 2-(2-Bromoethyl)-3-methyl-oxirane | C5H9BrO | Epoxide | DSI | 10.37 | 0.44 |
|  |  |  | KSC | 14.71 | 1.06 |
|  |  |  | NSRP | 14.08 | 0.57 |
| 2-Bromotetradecane | C14H29Br | Monoterpene | BG-24 | 15.75 | 2.45 |
|  |  |  | KRI | 12.41 | 0.47 |
|  |  |  | KSC | 12.85 | 1.14 |
| 2-Ethyl-1-dodecanol | C14H30O | Monoterpene | DSI | 20.19 | 0.85 |
|  |  |  | NRC | 14.14 | 1.47 |
|  |  |  | NSRP | 15.16 | 1.01 |
| 3,7,11,15-trimethylhexadecanoic acid, 2,2,2- trifluoroethyl ester | C22H41F3O2 | Ester (Fluoric acid) | BG-24 | 15.25 | 1.83 |
|  |  |  | ESC | 16.45 | 0.73 |
|  |  |  | NRC | 14.70 | 0.44 |
| 3-Chloropropionic acid, tetradecyl ester | C17H33ClO2 | Ester | ESC | 10.35 | 0.27 |
|  |  |  | KSC | 13.00 | 0.95 |
|  |  |  | NqSC | 14.15 | 0.57 |
| 3-Trifluoroacetoxy-6-ethyldecane | C14H25F3O2 | Ester (Fluoric acid) | KRI | 12.68 | 0.37 |
|  |  |  | NqSC | 12.68 | 0.96 |
|  |  |  | NSRC | 13.01 | 0.86 |
| 4-Fluoro-1-methyl-5-carboxylic acid, ethyl(ester) | C7H9FN2O2 | Ester | DSI | 15.74 | 0.29 |
|  |  |  | KSC | 15.53 | 0.63 |
|  |  |  | NSRC | 13.86 | 0.86 |
| 4-Methyldocosane | C23H48 | Diterpene | DSI | 11.13 | 0.44 |
|  |  |  | ESC | 14.78 | 0.48 |
|  |  |  | NqSC | 15.09 | 1.27 |
| 6,8a-Epidioxy-4a-methyl-2-oxo-3,4,4a,5,6,7,8,8a-octahydro-2H-1-benzofur | C10H14O4 | Coumarin | BG-31 | 15.94 | 0.37 |
|  |  |  | BG-100/GC | 15.90 | 1.02 |
|  |  |  | ESC | 15.04 | 0.92 |
| 6-Hydroxy-7-methyl-9-oxabicyclo[3.3.1]nonan-2-one | C9H14O3 | Hydroxycoumarin | BG-31 | 14.71 | 0.74 |
|  |  |  | KRI | 12.63 | 0.27 |
|  |  |  | NRC | 14.70 | 0.51 |
| Acetic acid, trifluoro-, undecyl ester | C13H23F3O2 | Ester (Fluoric acid) | BG-31 | 10.44 | 0.78 |
|  |  |  | BG-70 | 10.34 | 1.49 |
|  |  |  | DSI | 14.85 | 3.27 |
| Bacteriochlorophyll-c-stearyl | C52H72MgN4O4 | Tetraterpene | KRI | 14.68 | 0.37 |
|  |  |  | ESC | 16.11 | 0.58 |
|  |  |  | NqSC | 12.82 | 0.25 |
| Bicyclo[2.2.2]octanone, 4-methoxy-1-methyl- | C10H16O2 | Monoterpene | BG-31 | 15.54 | 0.36 |
|  |  |  | ESC | 25.19 | 0.31 |
|  |  |  | NSRP | 13.65 | 2.55 |
| Borane, 2,3-dimethyl-2-butyl- (dimer) | C12H30B2 | Organic metalloid | BG-31 | 10.34 | 0.55 |
|  |  |  | KRI | 25.18 | 1.09 |
|  |  |  | KSC | 15.39 | 0.64 |
| Butyl 9-decenoate | C14H26O2 | Fatty acid ester | BG-31 | 16.26 | 0.17 |
|  |  |  | KSC | 14.10 | 1.44 |
|  |  |  | NqSC | 18.57 | 0.64 |
| Cyclodecane | C10H20 | Monoterpene | BG-24 | 9.09 | 1.85 |
|  |  |  | BG-100/GC | 9.10 | 1.01 |
|  |  |  | NSRC | 9.11 | 2.02 |
| Cyclohexane, (1-butylhexadecyl)- | C26H52 | Sesterterpene | KRI | 14.21 | 0.85 |
|  |  |  | KSC | 15.04 | 0.85 |
|  |  |  | KSP | 14.21 | 1.68 |
| Cyclohexane, 1,2,4,5-tetraethyl- | C14H28 | Monoterpene | BG-100/GC | 15.52 | 0.83 |
|  |  |  | DSI | 25.19 | 0.60 |
|  |  |  | KRI | 15.09 | 0.58 |
| Cyclopentane, 1-(2-decyldodecyl)-2,4-dimethyl | C29H58 | Sesterterpene | BG-70 | 14.85 | 0.70 |
|  |  |  | KSC | 14.84 | 2.12 |
|  |  |  | NRC | 15.03 | 0.67 |
| Cyclopentane, 1-pentyl-2-propyl- | C13H26 | Monoterpene | BG-31 | 12.83 | 0.46 |
|  |  |  | BG-100/GC | 10.34 | 0.75 |
|  |  |  | KRI | 10.33 | 0.72 |
| Decane, 2,3,5,8-tetramethyl- | C14H30 | Monoterpene | BG-100/GC | 10.02 | 1.49 |
|  |  |  | KRI | 10.02 | 1.27 |
|  |  |  | NqSC | 10.01 | 1.14 |
| Dodecane, 2-methyl-6-propyl- | C16H34 | Sesquiterpene | BG-24 | 13.60 | 8.19 |
|  |  |  | BG-100/GC | 15.68 | 3.47 |
|  |  |  | DSI | 16.40 | 1.62 |
| Dodecane, 2-methyl-8-propyl- | C16H34 | Sesquiterpene | BG-31 | 13.22 | 1.14 |
|  |  |  | BG-70 | 14.68 | 1.27 |
|  |  |  | KRI | 15.03 | 0.76 |
| Eicosane, 2-methyl- | C21H44 | Diterpene | KRI | 14.20 | 1.43 |
|  |  |  | KSP | 14.02 | 1.72 |
|  |  |  | NRC | 13.21 | 1.01 |
| erythro-7,8-Bromochlorodisparlure | C19H38BrCl | Organochlorides | BG-100/GC | 14.93 | 0.72 |
|  |  |  | NRC | 25.22 | 0.72 |
|  |  |  | NSRC | 23.71 | 0.77 |
| Heptadecane, 8-methyl- | C18H38 | Sesquiterpene | BG-24 | 14.28 | 0.63 |
|  |  |  | BG-31 | 12.86 | 1.08 |
|  |  |  | BG-100/GC | 15.76 | 1.30 |
| Heptadecane, 9-octyl- | C25H52 | Sesterterpene | ESC | 23.70 | 0.73 |
|  |  |  | KRI | 15.06 | 1.26 |
|  |  |  | NRC | 13.10 | 1.02 |
| Phytane | C20H42 | Diterpene | ESC | 15.75 | 2.68 |
|  |  |  | KSP | 14.02 | 2.01 |
|  |  |  | NqSC | 11.20 | 0.47 |
| Hexadecane, 2,6,11,15-tetramethyl- | C20H42 | Diterpene | KSC | 12.54 | 1.79 |
|  |  |  | NSRC | 15.75 | 2.28 |
|  |  |  | NSRP | 19.03 | 1.04 |
| n-Hexadecanoic acid | C17H34O2 | Fatty acid | DSI | 18.49 | 1.13 |
|  |  |  | KSC | 18.50 | 0.45 |
|  |  |  | NqSC | 18.47 | 2.30 |
| Nonadecane, 2-methyl- | C20H42 | Diterpene | BG-24 | 14.04 | 1.67 |
|  |  |  | KRI | 14.57 | 4.26 |
|  |  |  | KSP | 16.93 | 4.31 |
| Nonane, 2-methyl- | C10H22 | Monoterpene | KRI | 13.04 | 0.47 |
|  |  |  | KSC | 14.13 | 0.57 |
|  |  |  | NSRP | 10.51 | 0.40 |
| Octadecane, 2,6-dimethyl- | C20H42 | Diterpene | BG-24 | 14.04 | 1.64 |
|  |  |  | DSI | 15.68 | 0.89 |
|  |  |  | KSC | 13.09 | 0.78 |
| Pentadecane, 5-methyl- | C16H34 | Sesquiterpene | BG-70 | 13.10 | 0.69 |
|  |  |  | KRI | 13.09 | 0.75 |
|  |  |  | KSC | 12.97 | 1.04 |
| Silane, trichlorodocosyl- | C22H45Cl3Si | Organosilicon | BG-70 | 14.22 | 0.95 |
|  |  |  | NRC | 15.74 | 2.06 |
|  |  |  | NSRC | 15.26 | 0.85 |
| Sulfurous acid, 2-propyl tetradecyl ester | C17H36O3S | Ester (Sulfurous acid) | BG-100/GC | 14.21 | 1.13 |
|  |  |  | KSC | 14.20 | 1.68 |
|  |  |  | NqSC | 14.20 | 1.17 |
| Sulfurous acid, dodecyl hexyl ester | C18H38O3S | Ester (Sulfurous acid) | BG-100/GC | 10.51 | 0.47 |
|  |  |  | ESC | 13.10 | 0.82 |
|  |  |  | NqSC | 10.50 | 0.47 |
| Sulfurous acid, hexyl pentadecyl ester | C21H44O3S | Ester (Sulfurous acid) | BG-31 | 12.12 | 1.11 |
|  |  |  | BG-70 | 13.01 | 1.15 |
|  |  |  | NSRC | 12.43 | 0.68 |
| Tetradecane, 6,9-dimethyl- | C16H34 | Sesquiterpene | BG-31 | 14.03 | 1.51 |
|  |  |  | BG-100/GC | 19.04 | 0.75 |
|  |  |  | NSRC | 14.04 | 2.19 |
| Tetratetracontane | C44H90 | Polyterpene | BG-70 | 21.70 | 0.76 |
|  |  |  | KRI | 14.40 | 0.52 |
|  |  |  | NRC | 14.27 | 0.65 |
| Tridecane, 5-propyl- | C16H34 | Sesquiterpene | BG-100/GC | 14.03 | 1.19 |
|  |  |  | KSP | 14.03 | 1.51 |
|  |  |  | NSRC | 14.03 | 1.42 |
| Undecane, 6-cyclohexyl- | C17H34 | Sesquiterpene | KRI | 14.93 | 0.72 |
|  |  |  | KSC | 13.91 | 0.30 |
|  |  |  | NRC | 25.19 | 1.02 |

Landraces are described in Table 1. Rt – Retention time (min) and RPA – Relative peak area (%).

Supplementary Data 2l: Phytochemical compounds shared among two landraces.

| Phytochemical compound | Chemical formula | Chemical Group | Landrace | Rt(mins) | RPA (%) |
| --- | --- | --- | --- | --- | --- |
| 1-(5-Ethyl-tetrahydrofuran-2-yl)-3,3-dimethyl-butan-2-one | C12H22O2 | Cyclic ether | BG-100/GC | 14.70 | 0.35 |
|  |  |  | NSRC | 14.14 | 1.32 |
| 1,1,1-Trifluoroheptadecen-2-one | C17H31F3O | Ketone | BG-31 | 12.69 | 0.34 |
|  |  |  | NRC | 16.65 | 0.29 |
| 1,1-Cyclopropanedicarboxamide | C5H8N2O2 | Cyclic amide | KSC | 15.49 | 0.11 |
|  |  |  | NRC | 23.47 | 0.58 |
| 1,3-Di(cyclohexyl)but-1-ene | C16H28 | Sesquiterpene | ESC | 23.68 | 1.01 |
|  |  |  | KSC | 16.15 | 0.56 |
| 1,4-Cyclododecanedione | C12H20O2 | Cyclic ketone | ESC | 25.27 | 0.51 |
|  |  |  | NqSC | 25.15 | 0.74 |
| 1,4-Diaza-9-oxaspiro[5.5]undecane, 8-ethyl-8-methyl- | C11H22N2O | Heterocyclic | KSC | 13.96 | 0.24 |
|  |  |  | NSRC | 14.70 | 0.80 |
| 10-Heneicosene (c,t) | C21H42 | Diterpene | BG-31 | 18.92 | 1.99 |
|  |  |  | BG-100/GC | 18.91 | 5.17 |
| 10-Methyldodecan-5-olide | C13H24O2 | Fatty ester | ESC | 25.24 | 0.27 |
|  |  |  | NSRC | 15.34 | 0.61 |
| 10-Methylnonadecane | C20H42 | Diterpene | BG-31 | 15.76 | 2.51 |
|  |  |  | NSRP | 10.02 | 0.74 |
| 13-Tetradecen-1-ol acetate | C16H30O2 | Ester | BG-70 | 25.24 | 1.82 |
|  |  |  | BG-100/GC | 25.23 | 1.15 |
| 1-Chloro-1-n-decyloxy-1-silacyclopentane | C14H29ClOSi | Organosilicon | KSC | 13.70 | 0.35 |
|  |  |  | NqSC | 25.21 | 1.18 |
| 1-Decanol, 2-ethyl- | C12H26O | Monoterpene | BG-31 | 15.17 | 1.58 |
|  |  |  | NRC | 15.15 | 1.00 |
| 1-Decanol, 2-methyl- | C11H24O | Monoterpene | DSI | 10.00 | 0.54 |
|  |  |  | KSC | 15.24 | 1.95 |
| 1-Dodecanol, 2-octyl- | C20H42O | Diterpene | BG-70 | 25.48 | 0.78 |
|  |  |  | NSRC | 15.17 | 0.77 |
| 1-Octanol, 2-methyl- | C9H20O | Isoprene unit | BG-100/GC | 10.37 | 0.38 |
|  |  |  | NqSC | 18.61 | 0.55 |
| 1-Octanone, 1-(2-octylcyclopropyl)- | C19H36O | Sesquiterpene | ESC | 14.68 | 0.42 |
|  |  |  | KSP | 14.68 | 1.03 |
| 1-Tetradecene, 2-decyl- | C24H48 | Diterpene | BG-31 | 15.05 | 1.27 |
|  |  |  | ESC | 16.25 | 0.56 |
| 1-Undecene, 8-methyl- | C12H24 | Monoterpene | BG-100/GC | 10.45 | 1.07 |
|  |  |  | KSC | 10.43 | 0.71 |
| 2,2,4,5,5-Pentamethyl-3-imidazoline-1-oxyl | C8H15N2O | Heterocyclic | BG-31 | 10.35 | 0.95 |
|  |  |  | ESC | 25.24 | 1.01 |
| 2,4,6-Tris(cyclohexyl)hept-1-ene | C25H44 | Sesterterpene | KSC | 15.89 | 0.70 |
|  |  |  | NRC | 15.83 | 0.37 |
| 2,6,6,10-Tetramethyl-undeca-8,10-diene-3,7-dione | C15H24O2 | Sesquiterpene | BG-24 | 15.13 | 0.14 |
|  |  |  | BG-100/GC | 16.66 | 0.27 |
| 2-Allyl-3-hydroxy-2-methylsuccinic acid, 1-ethyl ester | C10H16O5 | Carboxylic acid | BG-70 | 13.68 | 1.10 |
|  |  |  | BG-100/GC | 14.68 | 0.45 |
| 2-Bromo dodecane | C12H25Br | Monoterpene | BG-31 | 10.51 | 0.85 |
|  |  |  | KRI | 10.51 | 0.90 |
| 2H-Pyran-2-carboxylic acid, 3,6-dihydro-6-propoxy-, butyl ester | C13H22O4 | Phenolic acid | BG-100/GC | 14.77 | 0.69 |
|  |  |  | ESC | 14.44 | 0.78 |
| 3,3,3-Trifluoro-N-(2-fluorophenyl)-2-(trifluoromethyl)propionamide | C10H6F7NO | Cyclic amide | DSI | 10.87 | 0.66 |
|  |  |  | KSP | 15.85 | 0.31 |
| 3,3-Diethylheptadecane | C21H44 | Diterpene | KSC | 13.67 | 0.51 |
|  |  |  | NSRC | 13.65 | 1.78 |
| 3,4-Nonanedione, 5-ethyl-2-methyl-, 4-oxime | C12H23NO2 | Ketoxime | BG-100/GC | 15.74 | 0.92 |
|  |  |  | NqSC | 13.32 | 0.39 |
| 3-Ethyl-6-trifluoroacetoxyoctane | C12H21F3O2 | Ester | BG-31 | 15.47 | 0.14 |
|  |  |  | ESC | 14.88 | 0.43 |
| 3-Methyl-4-(phenylthio)-2-prop-2-enyl-2,5-dihydrothiophene 1,1-dioxide | C14H16O2S2 | Furan | DSI | 5.31 | 3.30 |
|  |  |  | KRI | 16.17 | 0.66 |
| 4(1H)-Isobenzofuranone, hexahydro-3a,7a-dimethyl-, cis-(.+/-.)- | C10H16O2 | Benzofuran | BG-70 | 23.37 | 0.64 |
|  |  |  | BG-100/GC | 16.05 | 0.78 |
| 4-Ethyl-3-methyl-1-octen-5-one | C11H20O | Monoterpene | KSC | 13.85 | 0.28 |
|  |  |  | NqSC | 13.16 | 0.39 |
| 4-Hydroxy-1,3-dimethyl-piperidine-4-carbonitrile | C8H14N2O | Heterocyclic amine | BG-31 | 15.95 | 0.64 |
|  |  |  | NSRP | 14.31 | 0.21 |
| 5,5-Diethylheptadecane | C21H44 | Diterpene | ESC | 15.75 | 2.92 |
|  |  |  | NSRC | 15.68 | 2.42 |
| 7-Oxabicyclo[2.2.1]heptane, 1-methyl-4-(1-methylethyl)- | C10H18O | Monoterpene | KSP | 10.34 | 0.87 |
|  |  |  | NqSC | 13.72 | 0.26 |
| 8-Azabicyclo[5.1.0]octane | C7H13N | Cyclic amine | BG-31 | 16.27 | 0.14 |
|  |  |  | NSRP | 13.78 | 0.73 |
| Aspidospermidin-17-ol, 1-acetyl-19,21-epoxy-15,16-dimethoxy- | C23H30N2O5 | Alkaloid | DSI | 15.37 | 0.36 |
|  |  |  | NSRP | 14.78 | 0.57 |
| Behenyl chloride | C22H45Cl | Chlorinated diterpene | KSC | 14.27 | 1.64 |
|  |  |  | NSRC | 23.69 | 1.58 |
| Benzene, 1-(dodecyloxy)-2-nitro- | C18H29NO3 | Cyclic ether | KSC | 13.91 | 0.43 |
|  |  |  | KSP | 13.29 | 0.36 |
| Bicyclo[2.2.2]octane-1,4-diol | C8H14O2 | Cyclic alcohol | KRI | 14.08 | 0.22 |
|  |  |  | KSC | 16.28 | 0.60 |
| Bromoacetic acid, octyl ester | C10H19BrO2 | Ester | BG-100/GC | 14.71 | 1.40 |
|  |  |  | NqSC | 15.53 | 0.90 |
| Butanoic acid, 3-methyl-, 2-hexenyl ester, (E)- | C11H20O2 | Ester | BG-31 | 14.92 | 0.41 |
|  |  |  | BG-100/GC | 13.70 | 0.14 |
| Cycloheptanone imine, 2,2,7,7-tetramethyl- | C11H21N | Cyclic amine | BG-70 | 15.10 | 1.39 |
|  |  |  | NqSC | 14.11 | 0.33 |
| Cyclohexane, (1-octylnonyl)- | C23H46 | Diterpene | DSI | 14.41 | 1.16 |
|  |  |  | ESC | 14.71 | 0.75 |
| Cyclohexanol, 1R-4-trans-acetamido-2,3-trans-epoxy- | C8H13NO3 | Hemiterpene | ESC | 15.37 | 0.42 |
|  |  |  | KRI | 13.72 | 0.46 |
| Cyclohexanol, 5-methyl-2-(1-methylethyl)-, (1.alpha.,2.beta.,5.alpha.)-(.+/-.)- | C10H20O | Monoterpene | BG-31 | 17.86 | 0.36 |
|  |  |  | NRC | 13.71 | 0.14 |
| Cyclohexanone, 2-(1-methyl-2-nitroethyl)- | C9H15NO3 | Hemiterpene | BG-31 | 14.82 | 0.93 |
|  |  |  | BG-100/GC | 16.79 | 0.51 |
| Cyclopropane, 1-(1-hydroxy-1-heptyl)-2-methylene-3-pentyl- | C16H30O | Sesquiterpene | KRI | 12.64 | 0.28 |
|  |  |  | KSC | 18.61 | 0.37 |
| Cyclopropanecarboxylic acid, 1-hydroxy-, (2,6-di-t-butyl-4-methylphenyl) ester | C19H28O3 | Sesquiterpene | DSI | 19.57 | 2.50 |
|  |  |  | KSC | 12.68 | 0.79 |
| Cyclopropanecarboxylic acid, 3-formyl-2,2-dimethyl-, ethyl ester | C9H14O3 | Hemiterpene | BG-31 | 14.44 | 0.15 |
|  |  |  | BG-100/GC | 16.52 | 0.69 |
| Decahydroquinolin-10-ol, N-acetyl- | C11H19NO2 | Alkaloid | KRI | 15.35 | 0.23 |
|  |  |  | KSC | 13.05 | 0.42 |
| Decane, 1-(ethenyloxy)- | C12H24O | Monoterpene | BG-70 | 15.17 | 2.17 |
|  |  |  | NqSC | 15.25 | 0.57 |
| Decane, 1,1'-oxybis- | C20H42O | Diterpene | BG-31 | 15.10 | 1.64 |
|  |  |  | NqSC | 14.08 | 0.38 |
| Decane, 1-iodo- | C10H21I | Iodinated Monoterpene | BG-31 | 14.14 | 0.73 |
|  |  |  | ESC | 10.51 | 0.89 |
| Decane, 2,4,6-trimethyl- | C13H28 | Monoterpene | BG-31 | 10.02 | 0.51 |
|  |  |  | NSRP | 15.33 | 0.98 |
| Dichloroacetic acid, 4-tridecyl ester | C15H28Cl2O2 | Ester | KSC | 15.15 | 1.56 |
|  |  |  | NSRP | 14.84 | 0.92 |
| Dodecanal | C12H24O | Monoterpene | DSI | 16.16 | 0.25 |
|  |  |  | KRI | 25.21 | 0.30 |
| Dodecane, 1-iodo- | C12H25I | Iodinated Monoterpene | KSP | 10.51 | 1.59 |
|  |  |  | NSRC | 10.51 | 1.37 |
| Dodecane, 5,8-diethyl- | C16H34 | Sesquiterpene | KRI | 10.35 | 0.63 |
|  |  |  | NqSC | 13.04 | 0.46 |
| E-11,13-Tetradecadien-1-ol | C14H26O | Monoterpene | BG-70 | 9.10 | 0.85 |
|  |  |  | NSRC | 9.09 | 0.99 |
| Eicosane, 3-cyclohexyl- | C26H52 | Sesterterpene | BG-100/GC | 15.32 | 1.14 |
|  |  |  | KRI | 15.32 | 0.57 |
| Ethanone, 1-(1,2,2,3-tetramethylcyclopentyl)-, (1R-cis)- | C11H20O | Monoterpene | KSP | 10.34 | 1.40 |
|  |  |  | NSRC | 10.35 | 1.01 |
| Geranyl ethyl ether 1 | C12H22O | Monoterpene | BG-100/GC | 16.17 | 0.32 |
|  |  |  | KSP | 15.15 | 0.98 |
| Geranyl isovalerate | C15H26O2 | Sesquiterpene | BG-70 | 25.23 | 1.47 |
|  |  |  | NSRP | 16.06 | 1.98 |
| Heptacosane, 1-chloro- | C27H55Cl | Chlorinated Sesterterpene | BG-70 | 25.22 | 0.93 |
|  |  |  | NSRC | 14.28 | 0.56 |
| Heptadecane, 9-(2-cyclohexylethyl)- | C25H50 | Sesterterpene | ESC | 15.26 | 0.44 |
|  |  |  | KSC | 14.68 | 1.38 |
| Heptafluorobutyric acid, n-tetradecyl ester | C18H29F7O2 | Ester | KSC | 13.64 | 0.98 |
|  |  |  | NSRC | 14.85 | 1.04 |
| Hexadecane, 1,1-bis(dodecyloxy)- | C40H82O2 | Aliphatic ether | ESC | 9.07 | 0.41 |
|  |  |  | KSP | 14.21 | 1.14 |
| Hexadecane, 2-methyl- | C17H36 | Sesquiterpene | BG-24 | 14.28 | 0.63 |
|  |  |  | NRC | 14.20 | 1.45 |
| Hexane, 2,4,4-trimethyl- | C9H20 | Aliphatic hydrocarbon | DSI | 13.47 | 0.42 |
|  |  |  | NSRP | 14.28 | 0.63 |
| Cyclotetradecane, 1,7,11-trimethyl-4-(1-methylethyl)- | C20H40 | Diterpene | BG-100/GC | 14.84 | 1.33 |
|  |  |  | KSC | 14.33 | 0.32 |
| N-[3-[N-Aziridyl]propylidene]tetrahydrofurfurylamine | C10H18N2O | Heterocyclic amine | BG-100/GC | 13.68 | 0.24 |
|  |  |  | KRI | 13.64 | 1.68 |
| N-[3-Hexylaminopropyl]aziridine | C11H24N2 | Amine | NqSC | 13.16 | 0.59 |
|  |  |  | NRC | 7.84 | 0.48 |
| N-Guanylproline | C6H11N3O2 | Non-essential amino acid | BG-70 | 15.06 | 1.04 |
|  |  |  | NqSC | 25.24 | 1.74 |
| Nonadecane, 2,3-dimethyl- | C21H44 | Diterpene | ESC | 15.75 | 2.69 |
|  |  |  | NRC | 23.65 | 1.59 |
| Octacosyl trifluoroacetate | C30H57F3O2 | Ester | BG-31 | 12.72 | 0.99 |
|  |  |  | DSI | 10.44 | 0.70 |
| Octadecane, 1-bromo- | C18H37Br | Sesquiterpene | BG-100/GC | 16.45 | 0.55 |
|  |  |  | KSC | 15.23 | 1.80 |
| Octane, 2,3,6,7-tetramethyl- | C12H26 | Monoterpene | BG-24 | 10.57 | 1.76 |
|  |  |  | KSP | 10.57 | 2.74 |
| Oxalic acid, 6-ethyloct-3-yl ethyl ester | C14H26O4 | Ester | BG-24 | 15.10 | 1.19 |
|  |  |  | NSRC | 13.11 | 0.74 |
| Oxalic acid, allyl tetradecyl ester | C19H34O4 | Ester | BG-24 | 15.17 | 1.19 |
|  |  |  | NRC | 10.34 | 1.02 |
| Oxalic acid, dodecyl isohexyl ester | C20H38O4 | Ester | KSC | 15.08 | 1.79 |
|  |  |  | NqSC | 13.09 | 0.71 |
| Oxalic acid, hexyl tetradecyl ester | C22H42O4 | Ester | BG-100/GC | 13.10 | 0.81 |
|  |  |  | KSC | 13.09 | 0.55 |
| Oxalic acid, propyl tetradecyl ester | C19H36O4 | Ester | KRI | 13.09 | 0.68 |
|  |  |  | KSP | 13.00 | 1.45 |
| Pent-4-enoic acid, 2-(2-hydroxy-3-isobutoxypropyl)-, hydrazide | C12H24N2O3 | Monoterpene | ESC | 15.29 | 0.17 |
|  |  |  | NqSC | 13.68 | 0.60 |
| Pentadecane, 7-methyl- | C16H34 | Sesquiterpene | BG-100/GC | 14.03 | 1.58 |
|  |  |  | DSI | 14.03 | 2.97 |
| Pentadecane, 8-hexyl- | C21H44 | Diterpene | BG-70 | 15.75 | 2.47 |
|  |  |  | ESC | 15.24 | 0.82 |
| Pentafluoropropionic acid, heptadecyl ester | C20H35F5O2 | Fatty ester | BG-24 | 25.25 | 2.33 |
|  |  |  | KSC | 14.83 | 1.27 |
| Pentatriacontane, 13-docosenylidene- | C47H94 | Polyterpene | ESC | 14.94 | 0.95 |
|  |  |  | NSRP | 15.16 | 1.47 |
| Piperazine, 1-(1-methyl-4-piperidyl)- | C10H21N3 | Cyclic amine | ESC | 14.92 | 0.52 |
|  |  |  | KRI | 13.75 | 0.22 |
| Pregan-20-one, 2-hydroxy-5,6-epoxy-15-methyl- | C22H34O3 | Diterpene | ESC | 14.51 | 0.84 |
|  |  |  | NSRP | 15.88 | 0.74 |
| Sulfone, [2-isobutyl-5-(4-morpholyl)oxazol-4-yl](phenyl)- | C17H22N2O4S | Sesquiterpene | KSC | 15.82 | 0.43 |
|  |  |  | NSRP | 10.37 | 0.67 |
| Sulfurous acid, dodecyl 2-propyl ester | C15H32O3S | Ester | BG-31 | 15.25 | 1.78 |
|  |  |  | KSC | 13.00 | 0.59 |
| Sulfurous acid, hexyl tridecyl ester | C19H40O3S | Ester | BG-31 | 13.10 | 0.79 |
|  |  |  | ESC | 14.09 | 0.54 |
| Sulfurous acid, pentadecyl 2-propyl ester | C18H38O3S | Ester | KRI | 15.23 | 1.58 |
|  |  |  | KSP | 15.24 | 1.05 |
| Tetracosane, 1-bromo- | C24H49Br | Hydrocarbon | BG-70 | 15.75 | 2.43 |
|  |  |  | KSC | 15.15 | 1.95 |
| Tetracosyl trifluoroacetate | C26H49F3O2 | Fatty ester | BG-70 | 13.01 | 1.01 |
|  |  |  | BG-100/GC | 15.16 | 1.00 |
| Tetradecane, 4,11-dimethyl- | C16H34 | Sesquiterpene | KRI | 13.15 | 0.40 |
|  |  |  | KSC | 13.14 | 0.49 |
| Tetrahydroionyl acetate | C15H28O2 | Sesquiterpene | BG-31 | 15.14 | 0.22 |
|  |  |  | KRI | 16.15 | 0.66 |
| trans-4,5-Epoxydecane | C10H20O | Cyclic ether | ESC | 14.28 | 1.00 |
|  |  |  | NSRP | 15.47 | 1.95 |
| Trichloroacetic acid, pentadecyl ester | C17H31Cl3O2 | Ester | DSI | 18.91 | 2.65 |
|  |  |  | NRC | 12.85 | 1.13 |
| Tridecane, 2-methyl- | C14H30 | Monoterpene | BG-100/GC | 10.51 | 0.87 |
|  |  |  | KSC | 10.50 | 0.55 |
| Tridecane, 3-ethyl- | C15H32 | Sesquiterpene | BG-24 | 13.16 | 0.45 |
|  |  |  | ESC | 13.22 | 1.01 |
| Tridecanenitrile | C13H25N | Fatty nitrile | BG-100/GC | 15.85 | 0.76 |
|  |  |  | KSC | 13.70 | 0.50 |
| Undecane, 2,3-dimethyl- | C13H28 | Monoterpene | DSI | 14.56 | 1.27 |
|  |  |  | KSC | 13.58 | 5.32 |
| Undecane, 2,4-dimethyl- | C13H28 | Monoterpene | BG-24 | 15.69 | 2.91 |
|  |  |  | ESC | 9.16 | 0.36 |
| Undecane, 3,7-dimethyl- | C13H28 | Monoterpene | NRC | 11.50 | 2.41 |
|  |  |  | NSRC | 11.50 | 3.13 |
| Undecane, 4-ethyl- | C13H28 | Monoterpene | BG-31 | 11.50 | 1.93 |
|  |  |  | NSRP | 15.24 | 1.82 |
| Undecane, 5-ethyl-5-propyl- | C16H34 | Sesquiterpene | KSP | 10.50 | 0.88 |
|  |  |  | KSC | 15.74 | 2.05 |
| Z-5-Methyl-6-heneicosen-11-one | C22H42O | Ketone | KRI | 23.64 | 0.66 |
|  |  |  | NqSC | 13.03 | 0.11 |
| Cyclopentane, (2-hexyloctyl)- | C19H38 | Sesquiterpene | DSI | 14.21 | 0.40 |
|  |  |  | KSP | 15.15 | 0.88 |
| Pyridine-2,4-diol, 3,5,6-trimethyl- | C8H11NO2 | Pyrazole | BG-100/GC | 14.51 | 0.98 |
|  |  |  | NSRP | 14.76 | 0.33 |
| 2-Undecene, 2,5-dimethyl- | C13H26 | Monoterpene | BG-24 | 15.17 | 1.47 |
|  |  |  | BG-31 | 15.17 | 1.50 |

Landraces are described in Table 1. Rt – Retention time (min) and RPA – Relative peak area (%)
